# Diversity and taxonomic revision of methanogens and other archaea in the intestinal tract of terrestrial arthropods

**DOI:** 10.3389/fmicb.2023.1281628

**Published:** 2023-11-15

**Authors:** Evgenii Protasov, James O. Nonoh, Joana M. Kästle Silva, Undine S. Mies, Vincent Hervé, Carsten Dietrich, Kristina Lang, Lena Mikulski, Katja Platt, Anja Poehlein, Tim Köhler-Ramm, Edouard Miambi, Hamadi I. Boga, Christopher Feldewert, David K. Ngugi, Rudy Plarre, David Sillam-Dussès, Jan Šobotník, Rolf Daniel, Andreas Brune

**Affiliations:** ^1^Research Group Insect Gut Microbiology and Symbiosis, Max Planck Institute for Terrestrial Microbiology, Marburg, Germany; ^2^Genomic and Applied Microbiology and Göttingen Genomics Laboratory, Institute of Microbiology and Genetics, Georg August University of Göttingen, Göttingen, Germany; ^3^Evolutionary Ecology Department, Institute of Ecology and Environmental Sciences of Paris (iEES-Paris), University of Paris-Est Créteil (UPEC), Créteil, France; ^4^Bundesanstalt für Materialforschung und -prüfung, Berlin, Germany; ^5^Laboratory of Experimental and Comparative Ethology (LEEC), UR 4443, Université Sorbonne Paris Nord, Villetaneuse, France; ^6^Faculty of Tropical AgriSciences, Czech University of Life Sciences, Prague, Czechia

**Keywords:** archaea, methanogens, gut microbiota, termites, cockroaches, millipedes, Bathyarchaeia, Nitrososphaerales

## Abstract

Methane emission by terrestrial invertebrates is restricted to millipedes, termites, cockroaches, and scarab beetles. The arthropod-associated archaea known to date belong to the orders *Methanobacteriales, Methanomassiliicoccales, Methanomicrobiales*, and *Methanosarcinales*, and in a few cases also to non-methanogenic *Nitrososphaerales* and *Bathyarchaeales*. However, all major host groups are severely undersampled, and the taxonomy of existing lineages is not well developed. Full-length 16S rRNA gene sequences and genomes of arthropod-associated archaea are scarce, reference databases lack resolution, and the names of many taxa are either not validly published or under-classified and require revision. Here, we investigated the diversity of archaea in a wide range of methane-emitting arthropods, combining phylogenomic analysis of isolates and metagenome-assembled genomes (MAGs) with amplicon sequencing of full-length 16S rRNA genes. Our results allowed us to describe numerous new species in hitherto undescribed taxa among the orders *Methanobacteriales* (*Methanacia*, *Methanarmilla*, *Methanobaculum*, *Methanobinarius*, *Methanocatella*, *Methanoflexus*, *Methanorudis*, and *Methanovirga*, all gen. nova), *Methanomicrobiales* (*Methanofilum* and *Methanorbis*, both gen. nova), *Methanosarcinales* (*Methanofrustulum* and *Methanolapillus*, both gen. nova), *Methanomassiliicoccales* (*Methanomethylophilaceae* fam. nov., *Methanarcanum*, *Methanogranum*, *Methanomethylophilus*, *Methanomicula*, *Methanoplasma*, *Methanoprimaticola*, all gen. nova), and the new family *Bathycorpusculaceae* (*Bathycorpusculum* gen. nov.). Reclassification of amplicon libraries from this and previous studies using this new taxonomic framework revealed that arthropods harbor only CO_2_ and methyl-reducing hydrogenotrophic methanogens. Numerous genus-level lineages appear to be present exclusively in arthropods, suggesting long evolutionary trajectories with their termite, cockroach, and millipede hosts, and a radiation into various microhabitats and ecological niches provided by their digestive tracts (e.g., hindgut compartments, gut wall, or anaerobic protists). The distribution patterns among the different host groups are often complex, indicating a mixed mode of transmission and a parallel evolution of invertebrate and vertebrate-associated lineages.

## Introduction

Methanogenic archaea play an important role in the fermentative breakdown of organic matter (Müller et al., 2018). They are common constituents of the intestinal microbiota of both invertebrate and vertebrate animals, where they thrive on the products of bacterial fermentations, namely molecular hydrogen, formate, methanol, and methylamines (Brune, 2019; Chibani et al., 2022).

Methane emission by termites was documented half a century ago by the seminal work of Breznak and coworkers (Brune, 2019). Although the phenomenon attracted attention because of its implications for the global methane budget, methane emissions from termites are dwarfed by those from ruminants and wetlands. Subsequent surveys of other invertebrates revealed that methanogenesis is restricted to only a few distinct groups of terrestrial arthropods, namely millipedes, termites and cockroaches (Blattodea), and scarab beetles (Hackstein and Stumm, 1994; Hackstein and van Alen, 2018; Brune, 2019).

Methanogens in arthropod guts are typically restricted to specific hindgut compartments, where they are localized on the cuticular lining, attached to filamentous bacteria on the hindgut wall, and associated with anaerobic protists (ciliates in cockroaches and millipedes; flagellates in all termite families except Termitidae or higher termites) (e.g., Leadbetter and Breznak, 1996; Sprenger et al., 2000). They consist almost exclusively of uncultured representatives, which have been identified in 16S rRNA-based surveys as members of the orders *Methanobacteriales* (phylum *Methanobacteriota*), *Methanomicrobiales* and *Methanosarcinales* (both phylum “*Halobacteriota*”), and *Methanomassiliicoccales* (phylum “*Thermoplasmatota*”); only a few species of the genera *Methanobrevibacter* and *Methanimicrococcus* have been isolated in pure culture (see reviews by Brune, 2018, 2019; Hackstein and van Alen, 2018). Some studies also identified non-methanogenic *Bathyarchaeales* and *Nitrososphaerales* (both phylum *Thermoproteota*) (e.g., Friedrich et al., 2001; Loh et al., 2021).

Despite these efforts, all major host groups are severely undersampled, and the diversity of methanogens in arthropods remains poorly resolved. The reference databases lack resolution because both full-length 16S rRNA gene sequences and genomes of arthropod-associated archaea are scarce. Also, the taxonomy of existing lineages is not well developed, and the names of many taxa are provisional and not validly published, while other taxa are under-classified and require revision (Rinke et al., 2021).

To address these issues, we conducted a phylogenomic analysis of all archaeal genomes from arthropods using the taxonomic framework of the Genome Taxonomy Database (Parks et al., 2021), including a large number of metagenome-assembled genomes (MAGs) from termite guts (85 MAGs from 34 termite species) and diverse, so far undescribed, isolates obtained in our laboratory from cockroaches and millipedes. In parallel, we prepared full-length 16S rRNA gene libraries from more than 70 species of methane-emitting arthropods and incorporated them into the alignment of the SILVA database (version 138), together with full-length sequences from the termite gut metagenomes and unpublished clone libraries from our laboratory. Based on this comprehensive reference database, we reconstructed phylogenetic trees for all archaeal lineages in arthropod guts. In order to revise the taxonomy of the respective lineages, including a number of provisional *Candidatus* taxa from the literature, we then linked the lineages in the respective phylogenies via the 16S rRNA genes in the genomes, which allowed us to describe new species and higher taxa under the *Code of Nomenclature of Prokaryotes Described from Sequence Data* (SeqCode) (Hedlund et al., 2022; Whitman et al., 2022). Finally, we reclassified the archaeal 16S rRNA-gene libraries from arthropods guts from this study and selected datasets from the literature to provide an overview of the distribution of archaeal lineages across all host groups at the genus level.

## Results

High-throughput sequencing of long-read amplicon libraries of archaeal 16S rRNA genes from the intestinal tract of cockroaches, termites, and millipedes (47 species, Supplementary Tables 1, 4) and hitherto unpublished clone libraries of archaeal 16S rRNA genes from termites and millipedes (15 species, Supplementary Tables 1, 5) substantially expanded literature information on archaeal diversity in methane-emitting arthropods (18 species, Supplementary Table 5). Phylogenetic analysis revealed that the archaeal communities consist mostly of methanogenic archaea of the orders *Methanobacteriales, Methanomicrobiales, Methanosarcinales*, and *Methanomassiliicoccales*. In a few species of soil-feeding termites and litter-feeding millipedes, the archaeal communities comprised also non-methanogenic *Bathyarchaeales* and *Nitrososphaerales*.

**FIGURE 1:**
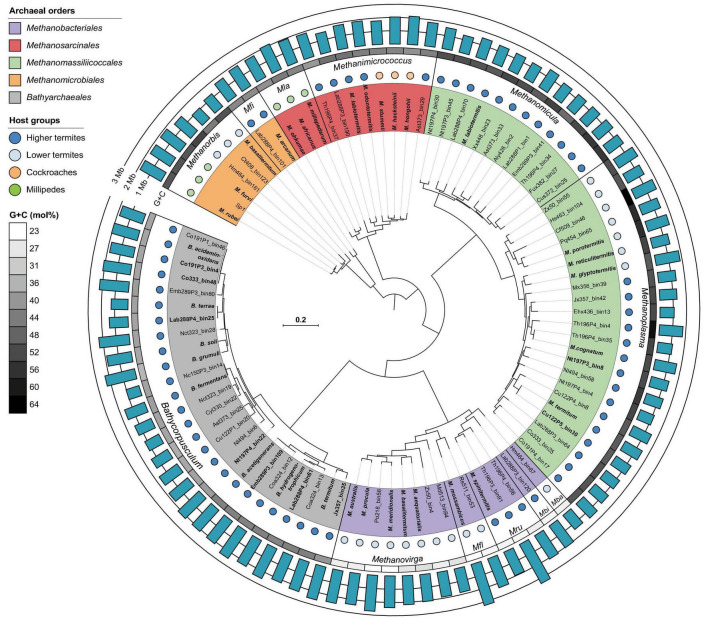
Phylogenomic analysis of archaeal genomes obtained from lower and higher termites, cockroaches, and millipedes. The tree is based on a concatenated alignment of 53 markers and was reconstructed with IQ-TREE under the LG + F + I + G4 model of evolution. High-quality genomes are in bold. For genome accession numbers and other details, see Supplementary Table 2. Mfi, *Methanofilum*; Mla, *Methanolapillus*; Mba, *Methanobaculum*; Mbi, *Methanobinarius*; Mru, *Methanorudis*; Mfl, *Methanoflexus*.

**FIGURE 2:**
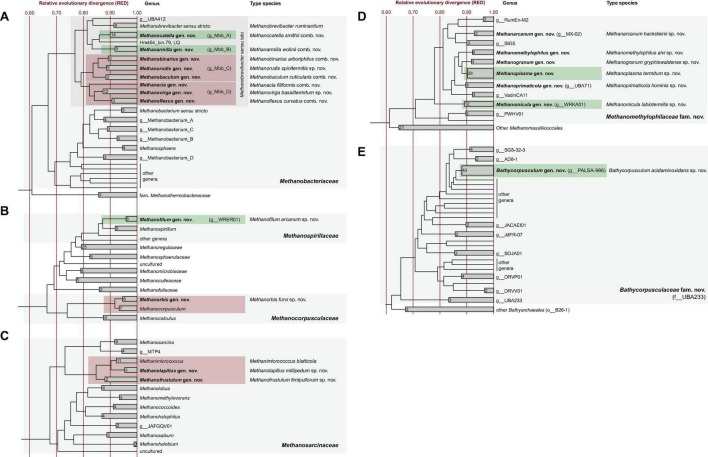
Rank-normalized phylogenies of the archaeal families that harbor isolates or MAGs from arthropod guts **(A–E)**. Taxa with representatives from arthropod guts are highlighted in color; green shading indicates genera that are recognized also in GTDB, red shading indicates those that are expanded in the present study. Newly proposed genera are in bold; the corresponding type species are given. Provisional names in the GTDB taxonomy are in parentheses. The maximum-likelihood tree is based on an alignment of 53 marker genes and was normalized using relative evolutionary divergence (RED) values determined with *PhyloRank*. The number of genomes in the collapsed clades is indicated. For an expanded version of the original tree, including genome accession numbers, see Supplementary Figure 1.

Phylogenomic analysis of 85 archaeal MAGs from gut metagenomes of 34 termite species and 9 genomes of methanogens isolated from 5 millipede and 3 cockroach species revealed that almost all genus-level lineages occurring in arthropods were represented by one or more high-quality genomes (Figure 1 and Supplementary Table 2). Since the relative evolutionary divergence (RED) of several genera considerably exceeded the average values of other genus-level lineages in the respective phyla (Parks et al., 2018; Rinke et al., 2021), we harmonized the taxonomic ranks of the respective lineages by introducing additional genus-level taxa (Figure 2).

**FIGURE 3:**
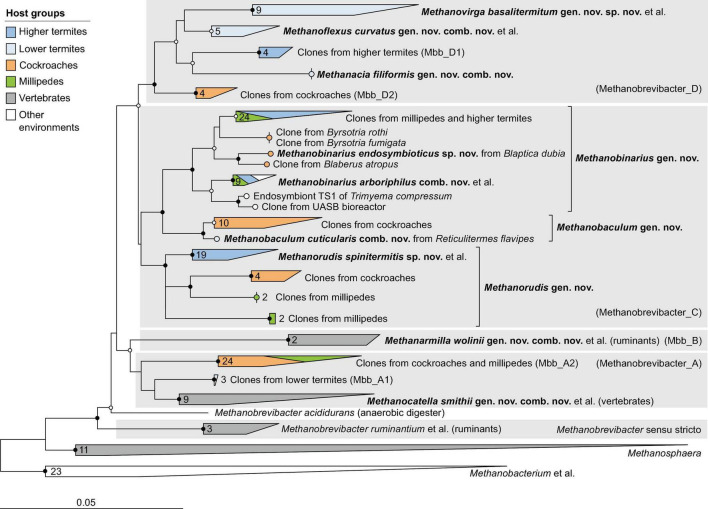
Phylogenetic tree of *Methanobacteriaceae*, illustrating the position of the sequences from arthropods obtained in this and previous studies. Other Methanobacteriales were used as outgroup. The maximum-likelihood tree is based on a curated alignment of near-full-length 16S rRNA genes (>1,400 sites) and was generated using IQ-TREE under the GTR + I + G4 model of evolution. Bullets on internal nodes indicate SH-aLRT/ultrafast bootstrap support (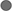, both ≥95/99%; ○, both ≥80/95%; 1,000 replicates each). The scale bar indicates the number of substitutions per site. Color coding indicates host groups. Newly described taxa and their type species are in bold; provisional names in the GTDB taxonomy in parentheses. The number of sequences in the collapsed clades is indicated. A fully expanded version of the tree, including accession numbers, is included in Supplementary Figure 3.

**FIGURE 4:**
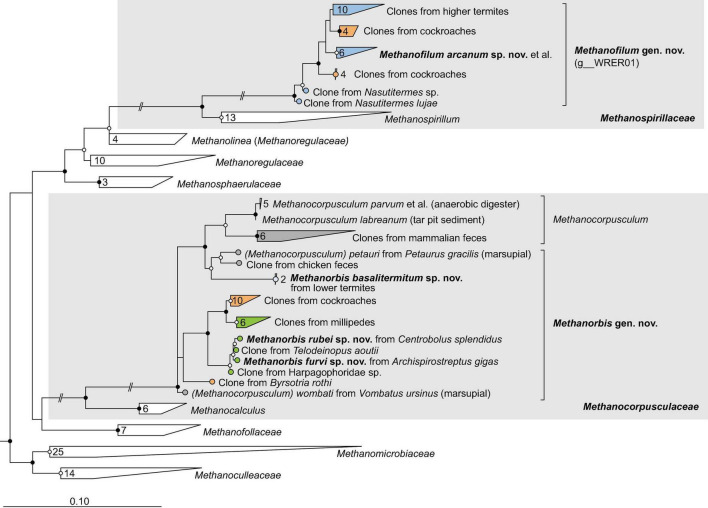
Phylogenetic tree of *Methanomicrobiales*, illustrating the position of the sequences from arthropods obtained in this and previous studies. *Methanocellales* were used as outgroup. Color coding and other details are the same as in Figure 1. A fully expanded version of the tree, including accession numbers, is included in Supplementary Figure 4.

**FIGURE 5:**
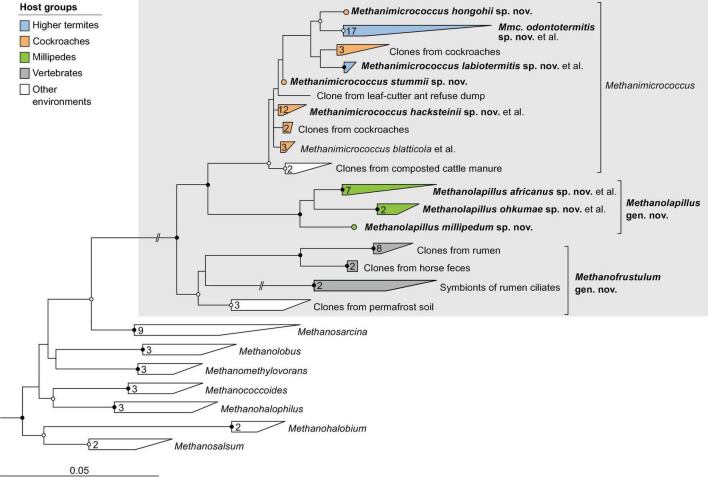
Phylogenetic tree of *Methanosarcinaceae* illustrating the position of the sequences from arthropods obtained in this and previous studies. Other *Methanosarcinales* were used as outgroup. Color coding and other details are the same as in Figure 1. A fully expanded version of the tree, including accession numbers, is included in Supplementary Figure 5.

**FIGURE 6:**
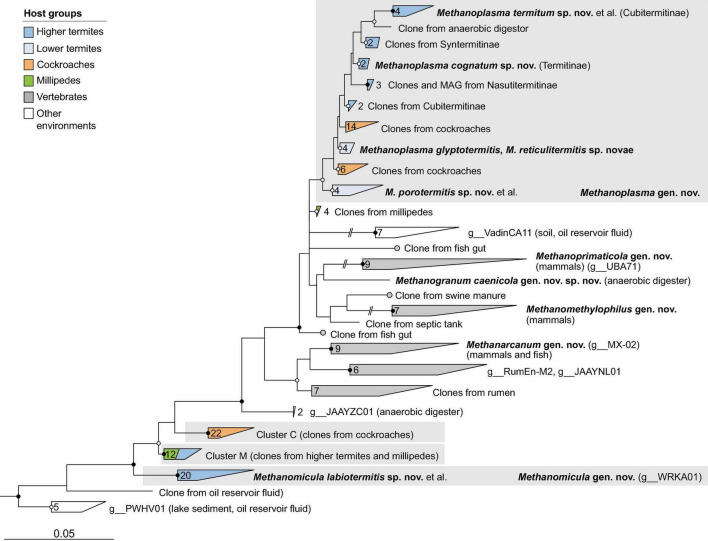
Phylogenetic tree of *Methanomethylophilaceae*, illustrating the position of the sequences from arthropods obtained in this and previous studies. Other *Methanomassiliicoccales* were used as outgroup. Color coding and other details are the same as in Figure 1. A fully expanded version of the tree, including accession numbers, is included in Supplementary Figure 6.

**FIGURE 7:**
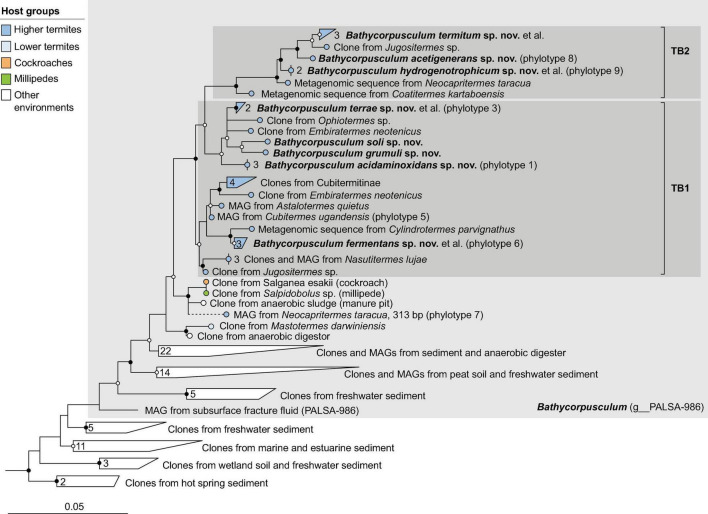
Phylogenetic tree of *Bathycorpusculaceae*, illustrating the position of the sequences from arthropods obtained in this and previous studies. Other *Bathyarchaeales* were used as an outgroup. Color coding and other details are the same as in Figure 1. A fully expanded version of the tree, including accession numbers, is included in Supplementary Figure 7.

Using the 16S rRNA genes from the genomic datasets, it was possible to link most clades in the 16S rRNA-based trees to this new taxonomic framework (Figures 3–7). In each order except Nitrososphaerales, the sequences from arthropod guts typically formed one or more lineages that comprised only members of a particular host group, often without cultured representatives. Most lineages had representatives with sequenced genomes of sufficient quality to serve as nomenclatural type for the description of new species under SeqCode (see section “Taxonomy”).

### Methanobacteriales

Members of the order *Methanobacteriales* are the most common archaeal lineage in the intestinal tract of arthropods. All phylotypes fall within the radiation of the genus *Methanobrevibacter* sensu lato (Figure 3). Based on the RED values among members of this genus (Rinke et al., 2021), the current Genome Taxonomy Database (GTDB) distinguishes between *Methanobrevibacter* sensu stricto (which contains the type species, *Methanobrevibacter ruminantium*) and four additional genus-level lineages, Methanobrevibacter_A to Methanobrevibacter_D (hereafter Mbb_A–D). All sequences from arthropod guts fall into the radiation of Mbb_A, Mbb_C, and Mbb_D; the lineages *Methanobrevibacter* sensu stricto and Mbb_B are not represented in arthropods.

In the phylogenomic analysis, the new MAGs from termite guts further expanded the evolutionary divergence within the radiation of Mbb_C and Mbb_D, resulting in RED values for the internal nodes that require the introduction of additional genus-level taxa (Figure 2A). In accordance with the taxonomic ranks suggested by Rinke et al. (2021), we propose that the *Methanobrevibacter* species that do not fall within the radiation of *Methanobrevibacter* sensu stricto be placed in the new genera *Methanocatella* (Mbb_A), *Methanarmilla* (Mbb_B), *Methanobaculum, Methanobinarius*, and *Methanorudis* (Mbb_C), and *Methanacia, Methanoflexus*, and *Methanovirga* (Mbb_D), using the genomes of previously described species and uncultured archaea as nomenclatural type (see section “Taxonomy”).

The genera *Methanocatella* and *Methanarmilla* consist exclusively of isolates or uncultured archaea from the intestinal tract of mammals (Figure 3). Mbb_A comprises a large clade of 16S rRNA sequences from cockroaches and a few millipedes (Mbb_A2) and a smaller clade (Mbb_A1) of sequences from lower termites (*Reticulitermes flavipes* and *Hodotermopsis sjoestedti*), which are well separated from the genus *Methanocatella* but lack representatives with high- or medium-quality genomes. In the phylogenomic analysis, we identified a single low-quality genome (Hm464_bin.79) from the lower termite *Hodotermes mossambicus* that occupies a sister position to the genus *Methanocatella*, suggesting that the Mbb_A clade comprises additional genus-level taxa from arthropod guts (Figure 2B).

The remaining genera consist almost exclusively of representatives from the guts of termites, cockroaches, and millipedes (Figure 3). While the genera *Methanacia, Methanobaculum, Methanobinarius*, and *Methanoflexus* have cultured representatives, the genera *Methanorudis* and *Methanovirga* consist exclusively of uncultured archaea. Two clades in the radiation of Mbb_D that consist exclusively of clones from cockroaches (Mbb_D2) and higher termites (Mbb_D1) lack representatives with sequenced genomes. A few clones from the genus *Methanobinarius* were not obtained from arthropods guts but were recovered from a sapropelic ciliate or anaerobic bioreactors.

### Methanomicrobiales

Representatives of the order *Methanomicrobiales* form several arthropod-specific clusters in the families *Methanospirillaceae* and *Methanocorpusculaceae* (Figure 4). The clones that fall into the radiation of *Methanospirillaceae* form a genus-level lineage that is sister to the genus *Methanospirillum* (Figure 4). The clade consists exclusively of uncultured methanogens from the intestinal tract of higher termites and several cockroaches. Since the MAGs from termite guts form a well-separated genus-level clade (WRER01 in GTDB) also in the phylogenomic tree (Figure 2B), we propose to classify them in the new genus *Methanofilum* (see section “Taxonomy”).

The clones that fall into the radiation of *Methanocorpusculaceae* form several lineages that occupy basal positions to the genus *Methanocorpusculum*. One lineage consists exclusively of sequences from millipedes, including three isolates from our laboratory (Protasov and Brune, unpublished results). It is loosely affiliated with additional lineages of uncultured representatives from cockroaches, millipedes, and termites. Members of the genus *Methanocorpusculum* form a well-supported cluster with a lineage of uncultured archaea from mammalian feces. In the phylogenomic analysis, however, only the genomes from mammalian feces fall into the genus *Methanocorpusculum*, whereas the MAGs from termites and the genomes of millipede isolates form a separate genus-level clade that also includes MAGs from wombat and chicken feces (Figure 2B). We propose to classify the members of this clade in the new genus *Methanorbis* (see section “Taxonomy”).

### Methanosarcinales

In the order *Methanosarcinales*, most sequences from arthropods guts fall into two genus-level clusters in the family *Methanosarcinaceae* (Figure 5). One of the clusters contains all representatives from termites and cockroaches, including *Methanimicrococcus blatticola* isolated from the cockroach *Periplaneta americana* (Sprenger et al., 2000; Figure 5). The cluster comprises the 16S rRNA genes of several MAGs from higher termites and three isolates from cockroaches that were obtained in our laboratory (Protasov and Brune, unpublished results); we propose to classify them as new species in the genus *Methanimicrococcus* (see section “Taxonomy”).

The second cluster consists exclusively of representatives from millipede guts, again including three isolates obtained in our laboratory (Protasov and Brune, unpublished results). Since the clade is well separated from the genus *Methanimicrococcus* also in the phylogenomic analysis (Figure 2C), we propose to classify the isolates as new species in the new genus *Methanolapillus* (see section “Taxonomy”).

The two clusters are sister to a clade of uncultured archaea from the rumen or feces of mammals, including endosymbionts of rumen ciliates. In the phylogenomic analysis (Figure 2C), the clade is represented by several genomes from ruminants and anaerobic digesters (Campanaro et al., 2020; Xie et al., 2021); we propose to classify these lineages in the new genus *Methanofrustulum* (see section “Taxonomy”).

### Methanomassiliicoccales

With a few exceptions, the *Methanomassiliicoccales* from arthropod guts are representatives of the so-called intestinal clade, a family-level cluster that comprises several highly enriched cultures but no isolates. Phylogenetic analysis revealed the presence of several arthropod-specific lineages that are well separated from lineages found in the mammalian guts or anaerobic digesters (Figure 6). One of these lineages comprises numerous representatives from the guts of termites, cockroaches, and millipedes, including the previously characterized *Candidatus* Methanoplasma termitum (Lang et al., 2015). Based on several genomes from lower and higher termites that form a well-separated clade in the phylogenomic tree (Figure 2D), we propose to place the members of this lineage in the new genus *Methanoplasma* and the new family *Methanomethylophilaceae* (see section “Taxonomy”).

Three other genus-level clusters that split off at basal nodes in the *Methanomethylophilaceae* tree consist exclusively of uncultured methanogens from arthropods (Figure 6). One cluster consists exclusively of representatives from cockroaches (cluster C), and another is a mixed cluster comprising sequences from millipedes and higher termites (cluster M). The third cluster consists exclusively of representatives from higher termites, including the 16S rRNA genes of several MAGs. Members of this cluster form a genus-level clade also in the phylogenomic analysis (Figure 2D) and are assigned to the new genus *Methanomicula* (see section “Taxonomy”).

### Bathyarchaeales

Members of the class *Bathyarchaeia* were represented exclusively in higher termites. In the 16S rRNA-based analysis, the clones fall within the radiation of two termite-specific clades previously described as *Ca.* Termiticorpusculum (TB1) and *Ca.* Termitimicrobium (TB2) in the recently described *Bathyarchaeales* (Loh et al., 2021; Khomyakova et al., 2023). Based on the 16S rRNA gene phylogeny, the phylotypes from termite guts represent a monophyletic group among various lineages of uncultured archaea from marine sediments, salt marshes, and anaerobic digesters (Figure 7). Phylogenomic analysis revealed that TB1 and TB2 are polyphyletic and separated by MAGs from hot spring sediments, anaerobic digesters, and permafrost soil (g__PALSA_986 in GTDB; Figure 2E). We propose to place members of the genus PALSA_986 in the new genus *Bathycorpusculum*, with *Bathycorpusculum acidaminoxidans* as type species (see section “Taxonomy”).

### Nitrososphaerales

**FIGURE 8:**
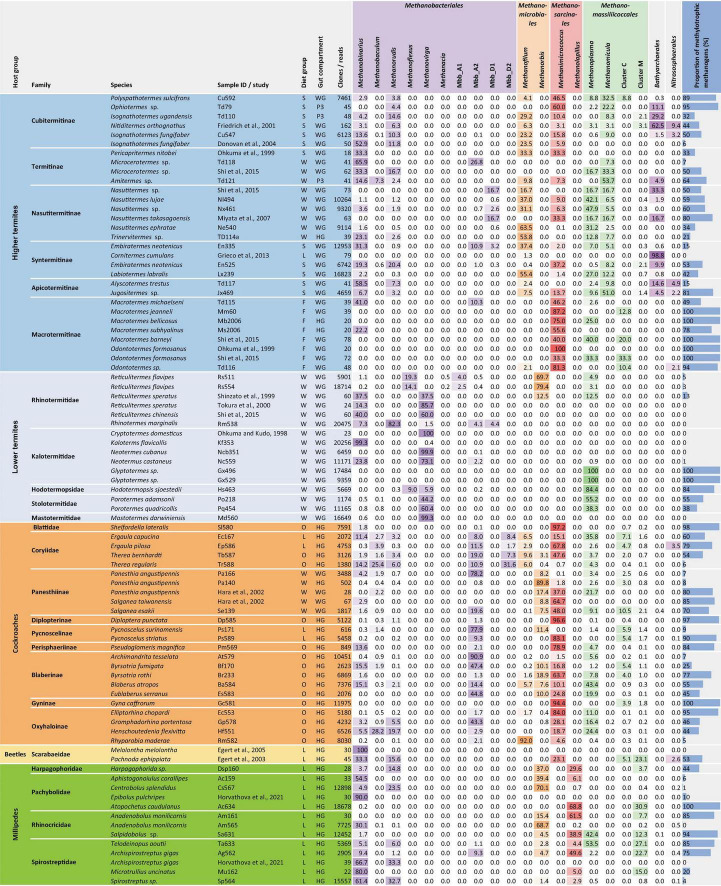
Relative abundance (%) of archaeal taxa in 16S rRNA gene libraries of various arthropods and the proportion of methylotrophs in the methanogenic community. The samples are sorted by host family and represent either whole guts (WG), entire hindguts (HG), or the largest of the proctodeal compartments (P3). Diet groups follow the classification of Arora et al. (2022). The number of clones/reads in the respective library is indicated. For more information and additional samples, see Supplementary Tables 3–5.

A small number of sequences from arthropod guts fall within the radiation of *Nitrososphaerales*, where they cluster with uncultured archaea in the genera *Candidatus* Nitrosocosmicus and g__UBA10452 (*Nitrosophaeraceae*) (Supplementary Figure 8). They were absent in most gut samples but were present in low abundance in several humivorous termites, millipedes, and the larva of the scarab beetle *Pachnoda ephippiata* (Figure 8 and Supplementary Tables 4, 5). In cases where individual compartments were sampled (soil-feeding termites of the genera *Amitermes*, *Isognathotermes*, *Polyspathotermes*, and *Ophiotermes*, and the humivorous larva of *P. ephippiata*), the same phylotypes dominated the clone libraries of food soil, nest material, and often also the anterior gut regions (Supplementary Table 3), suggesting that they are transient microbiota and originated from the environment.

### Distribution of archaeal groups across host lineages

We assessed archaeal community structure in methane-emitting arthropods by classifying the 16S rRNA gene libraries obtained in this and previous studies using the phylogenetic framework of our curated reference database (Supplementary Tables 4, 5). A comparison of representative samples from all host groups revealed that the distribution of methanogenic taxa among arthropods is complex (Figure 8).

Members of *Methanobacteriales* are present in almost all arthropod species investigated but are unevenly distributed among host groups (Figure 8). While the genera *Methanobaculum*, *Methanobinarius*, and *Methanorudis* are present in all host groups, *Methanovirga* and *Methanoflexus* are present only in lower termites, and *Methanacia* only in *Reticulitermes* spp. The cockroach cluster (Mbb_A2), which is related to the genus *Methanocatella*, also contains representatives from several termites and millipedes. Although *Methanobacteriales* dominate the archaeal community in numerous representatives of each host group, they are frequently outnumbered by members of other orders even in closely related hosts. Most striking are the large differences in the occurrence of certain genera between independent samples of the same host species (e.g., *Isognathotermes fungifaber*, *Embiratermes neotenicus*, *Panesthia angustipennis* and *Anadenobolus monilicornis*), which corroborates that the specificity of both hosts and symbionts for their respective partners is not always strict. The complete absence of *Methanobacteriales* from the amplicon libraries of the cockroach *Gyna caffrorum* and two *Glyptotermes* species is noteworthy (Supplementary Table 4), whereas their absence from several clone libraries should be interpreted with caution because of insufficient sampling depth (Supplementary Table 5).

Representatives of *Methanomicrobiales* are common in higher termites and millipedes and of lower abundance in cockroaches (Figure 8). Members of the genus *Methanorbis* occur in millipedes and cockroaches, where they often dominate the archaeal community, but are absent in termites, with the notable exception of several *Reticulitermes* species. The genus *Methanofilum*, which occurs in all higher termites and, although in lower abundance, also in many cockroaches, is not encountered in lower termites and millipedes.

The order *Methanosarcinales* is represented in all host groups except lower termites (Figure 8). Members of the genus *Methanimicrococcus* are restricted to cockroaches and higher termites, where they often dominate the archaeal community, and the humivorous larva of the scarab beetle *P. ephippiata*. The genus *Methanolapillus* occurs exclusively in millipedes, where it frequently represents the predominant lineage of methanogens.

Members of the *Methanomassiliicoccales* occur in all host groups and can dominate the archaeal community in certain host species (Figure 8). Members of the genus *Methanoplasma* are found in termites, cockroaches, and millipedes, whereas the genus *Methanomicula* occurs exclusively in higher termites, typically in high relative abundance. Members of cluster C, which occur at low abundance in most cockroaches, are also found in some Macrotermitinae (a subfamily of fungus-cultivating higher termites), whereas members of cluster M occur in millipedes, cockroaches, and soil-feeding higher termites. Members of both clusters are present in the humivorous larva of the scarab beetle *P. ephippiata*.

## Discussion

Our comprehensive analysis of the archaeal diversity in the intestinal tract of terrestrial arthropods known to emit methane reveals distinct clades of methanogens from the orders *Methanobacteriales, Methanomassiliicoccales, Methanomicrobiales*, and *Methanosarcinales*. Almost all lineages exhibit a high specificity for a particular host group (i.e., termites, cockroaches, or millipedes) and occupy sister positions to lineages from vertebrates, indicating a common evolutionary origin of host-associated methanogens. Linking the 16S rRNA-based diversity data to a phylogenomic analysis of more than 80 archaeal MAGs from termite guts and the genomes of 9 isolates from cockroaches and millipedes allowed the description of novel genera for each order and a taxonomic revision of methanogens and other archaea in arthropod guts.

### Taxonomic revision of the genus *Methanobrevibacter*

The first methanogens isolated from arthropod guts were members of the genus *Methanobrevibacter* (Leadbetter and Breznak, 1996; Leadbetter et al., 1998). Together with the 16S rRNA gene sequences of numerous uncultured representatives recovered from the intestinal tract of arthropods (reviewed in Brune, 2018), they represent lineages distinct from those that colonize the intestinal tract of vertebrates (Figure 3). Earlier studies using 16S rRNA and multi-locus gene sequence analyses had already suggested that the genus *Methanobrevibacter* is severely underclassified, comprising multiple genus-level clades that apparently coevolved with different host groups (i.e., ruminants, humans, and termites) (Dighe et al., 2004; Poehlein et al., 2018). This notion was then corroborated by a phylogenomic analysis that expanded the taxonomy of archaea to include genomes from uncultured lineages in the phylogenetic framework of GTDB and suggested that the high levels of RED within the genus *Methanobrevibacter* require the introduction of additional genus-level taxa (Rinke et al., 2021).

Our significantly expanded datasets of 16S rRNA gene sequences and MAGs from arthropod guts underscore the need for taxonomic revision. Based on the RED values of the internal nodes in the radiation of the genus *Methanobrevibacter* sensu lato, we propose to reclassify all species that do not fall into the *M. ruminantium* clade (comprising the type species of the genus *Methanobrevibacter*) into eight new genera: *Methanocatella* (Mbb_A), *Methanarmilla* (Mbb_B), *Methanobaculum*, *Methanobinarius*, and *Methanorudis* (Mbb_C), and *Methanacia*, *Methanoflexus*, and *Methanovirga* (Mbb_D) (Figure 2A). The presence of additional arthropod-specific clusters in the radiation of Mbb_A and Mbb_D (Figure 3), which lack representatives with sequenced genomes, suggests the presence of additional genus-level lineages that are candidates for future taxonomic revision. The same is true for the species *Methanobrevibacter acididurans*, which has no close relatives in public databases and whose genome remains to be sequenced.

Notably, each of the new genera is specific for a particular host group. While members of *Methanobrevibacter* sensu stricto, *Methanocatella*, and *Methanarmilla* are associated with the intestinal tract of ruminants and other vertebrates, all other genera are associated with arthropods and often include subclades restricted to either termites, cockroaches, or millipedes. The genera *Methanacia*, *Methanoflexus*, and *Methanovirga* currently consist exclusively of representatives from termites (Figure 3). The presence of a clade of unclassified phylotypes from higher termites (Mbb_D1) within the radiation of Mbb_D and another clade from cockroaches (Mbb_D2) in sister position to all clades from termites is in agreement with the evolutionary origin of termites among cockroaches (Inward et al., 2007) and a coevolutionary history of the members of the Mbb_D clade with their dictyopteran hosts.

The genera *Methanobaculum*, *Methanobinarius*, and *Methanorudis* also consist of lineages that are specific to particular arthropod host groups. In the genus *Methanobinarius*, the close relatedness among representatives from distantly related host lineages, i.e., termites (class Insecta) and millipedes (class Myriapoda), suggests an environmental transfer of methanogens between these soil-dwelling arthropods. This is underscored by the presence of *Methanobinarius* clones in anaerobic bioreactors (Carrillo-Reyes et al., 2014) and in the free-living, anaerobic ciliate *Trimyema compressum* (Shinzato et al., 2007).

Representatives of the genus *Methanosphaera*, which are typical for the intestinal tract of mammals (Hoedt et al., 2018; Thomas et al., 2022), were not detected in any arthropod host. Members of the genus *Methanobacterium* were only rarely encountered, indicating that the strains of *Methanobacterium bryantii* isolated from several higher termites (Deevong et al., 2004) are not common members of the archaeal microbiota of arthropods.

### New arthropod-specific genera in *Methanomicrobiales*

The order *Methanomicrobiales* comprises two so far unclassified arthropod-specific clades in the families *Methanocorpusculaceae* and *Methanospirillaceae*. In the phylogenomic analysis, the genomes from termites and millipedes are sister to the genus *Methanocorpusculum* (Figure 2B). A recent phylogenomic analysis revealed that the host-associated members of the genus *Methanocorpusculum* form two major clades (Volmer et al., 2023). Host clade 2, which is sister to the environmental clade comprising all described species of the genus *Methanocorpusculum*, consists entirely of uncultured archaea from mammalian feces. Host clade 1 consists of genomes from the feces of chickens and marsupials, including *Ca.* Methanocorpusculum faecipullorum and two isolates, *Methanocorpusculum petauri* and *Methanocorpusculum wombati* (Gilroy et al., 2021; Volmer et al., 2023). Since all members of host clade 1 fall within the radiation of genomes from millipedes and termites and are well separated from the remaining members of the genus *Methanocorpusculum* (Supplementary Figure 2), we propose to place them in the new genus *Methanorbis* (see section “Taxonomy”).

Amplicon sequencing of archaea in the intestinal tract of vertebrates has suggested that ancestral members of the genus *Methanocorpusculum* were present in the last common ancestor of ungulates (Thomas et al., 2022). Our results support a common ancestry of both arthropod-associated and vertebrate-associated *Methanocorpusculaceae*. Although the genera *Methanorbis* and *Methanocorpusculum* are not fully resolved in 16S rRNA-based analyses (Figure 4), phylogenomic analysis places all genomes from arthropods, including all MAGs from termite guts, into the genus *Methanorbis* (Supplementary Figure 2). The two sister clades of uncultured *Methanocorpusculaceae* from cockroaches and millipedes that lack representatives with sequenced genomes most likely belong also to the genus *Methanorbis*. The widespread distribution of *Methanorbis* in cockroaches and millipedes suggests that the representatives associated with chicken and marsupial feces were acquired by consuming arthropods. So far, it remains open whether the genus *Methanocorpusculum*, including the environmental clade, originated from a free-living or an arthropod-associated ancestor.

Members of the new genus *Methanofilum* occur exclusively in cockroaches and higher termites (Figures 4, 8). The absence of *Methanospirillaceae* in the intestinal tract of all other animals suggests that the genus arose within the order Blattodea, descending from free-living ancestors that occurred in their soil environment. This would mirror the situation with the genus *Methanosphaera*, which occurs exclusively in vertebrates and presumably evolved from an ancestral lineage of *Methanobacteriaceae* (Hoedt et al., 2018). The genus *Methanomicrobium*, which is common in the gut of ruminants (Janssen and Kirs, 2008; Henderson et al., 2015), is not represented in arthropods.

### Novel lineages of host-associated *Methanosarcinales*

Arthropod-associated members of *Methanosarcinales*, which were first detected in archaeal clone libraries of higher termites (Ohkuma et al., 1999; Friedrich et al., 2001), cockroaches (Sprenger et al., 2000), and scarab beetle larvae (Egert et al., 2003), are abundant in all host groups except lower termites (Figure 8). The genus *Methanimicrococcus* comprises all representatives from cockroaches and higher termites, whereas the new genus *Methanolapillus* harbors those associated with millipedes (Figure 4), suggesting that the lineages in Insecta and Diplopoda evolved independently from each other.

Unlike the sister genus *Methanosarcina*, whose members have the widest substrate range among methanogens, all isolates and genomes in the host-associated genera *Methanimicrococcus* and *Methanolapillus* examined to date are obligately hydrogen-dependent methylotrophs. Genomic analysis of the uncultivated representatives of the mammal-associated genus *Methanofrustulum* is pending, but all arthropod-associated lineages have lost the methyl branch of the Wood–Ljungdahl pathway and use methanol and methylamines as substrates only in the presence of hydrogen (Sprenger et al., 2007; Thomas et al., 2021; Protasov and Brune, unpublished results).

The arthropod-specific genera occupy a sister position to the new genus *Methanofrustulum*, whose representatives were first detected in horses but are found also in ruminants and other ungulates (Lwin and Matsui, 2014; Huang et al., 2016), where they have been identified as endosymbionts of rumen ciliates (Regensbogenova et al., 2004). The wide distribution of *Methanimicrococcus*-related archaea (most likely representing *Methanofrustulum*) in short-read amplicon libraries of vertebrates (Thomas et al., 2022) suggests parallel evolution of gut-associated *Methanosarcinaceae* in vertebrates and arthropods.

### *Methanomassiliicoccales* – Taxonomic update of the intestinal clade

The order *Methanomassiliicoccales* consists exclusively of obligately hydrogen-dependent methylotrophs (Zinke et al., 2021). It comprises two major clades that occur in contrasting environments. While members of the so-called environmental clade, represented by the family *Methanomassiliicoccaceae*, occur predominantly in anoxic soils, sediments, wetlands, and subsurface habitats, members of the so-called intestinal clade are found mostly in the guts of animals (Paul et al., 2012; Söllinger et al., 2016). The family name “*Methanomethylophilaceae*,” which was proposed based on a highly enriched culture from the human gut (Borrel et al., 2012; Gaci et al., 2014), has only candidate status so far because it contains no members with validly published names. Hence, we formally propose the new genus *Methanomethylophilus* as the type genus for the new family *Methanomethylophilaceae*, with the new species *Methanomethylophilus alvi* as the type species (see section “Taxonomy”). We also propose to include the new genera *Methanarcanum*, *Methanogranum*, *Methanoplasma*, and *Methanoprimaticola* in this family to accommodate other highly enriched cultures with sequenced genomes that so far have only *Candidatus* status (Iino et al., 2013; Lang et al., 2015; Weil et al., 2021; Chibani et al., 2022).

The genus *Methanoplasma*, which occurs exclusively in arthropod guts, belongs to an apical clade of the family *Methanomethylophilaceae*, with the genera *Methanogranum*, *Methanomethylophilus*, and *Methanoprimaticola* as its closest relatives (Figure 2). The same clade is also represented in the 16S rRNA-based analysis, although the branching order of its members is not fully resolved (Figure 6). Within the genus *Methanoplasma*, representatives from cockroaches and the phylogenetically older termite families branch more deeply than those from the phylogenetically younger higher termites, suggesting co-evolution between *Methanoplasma* and its blattodean hosts. An exception is the presence of an apical lineage in millipedes, which was most likely acquired by an environmental transfer from soil-feeding Cubitermitinae.

By contrast, the genus *Methanomicula*, which occurs exclusively in higher termites, occupies a basal position in the phylogeny of *Methanomethylophilaceae* (Figure 2). It contains no genomes from cockroaches and millipedes, but both host groups are represented in the 16S-based analyses (Figure 6). As in the case of the genus *Methanimicrococcus*, *Methanomicula* is also consistently absent in lower termites (Figure 8). While some older clone libraries described in the literature have been undersampled and suffer from primer bias against Methanomassiliicoccales (see discussion in Paul et al., 2012), the high abundance of *Methanoplasma* in amplicon libraries of lower termites suggests that the absence of *Methanomicula* is not an artifact.

### The genus *Bathycorpusculum* and description of the family *Bathycorpusculaceae*

Members of the “*Bathyarchaeota*,” a name coined by Meng et al. (2014) for the Miscellaneous Crenarchaeotal Group (MCG), are presently considered a class-level lineage in the phylum *Thermoproteota*. The class *Bathyarchaeia* was formally described only recently, based on its first cultured representative, *Bathyarchaeum tardum* isolated from the anaerobic sediment of a coastal lake (Khomyakova et al., 2023). The type genus, *Bathyarchaeum*, and its family, *Bathyarchaeceae* (formerly BA1), belong to the order *Bathyarchaeales* (formerly B26-1).

Members of the order *Bathyarchaeales*, at the time referred to as the “freshwater cluster” of the “*Crenarchaeota*,” were first detected in arthropod guts in archaeal clone libraries of soil-feeding termites (Friedrich et al., 2001). A genome-centric analysis of these termite-specific lineages identified them as members of the family UBA233 (also referred to as subgroup MCG-6 or Bathy-6) and tentatively assigned them to the candidate taxa “Termiticorpusculum” (TB1) and “Termitimicrobium” (TB2) (Loh et al., 2021). In the taxonomic framework of GTDB, they belong to a single genus-level lineage, described here as the new genus *Bathycorpusculum* (formerly PALSA-986), which also includes MAGs from peat soils, sediments, and anaerobic digesters, in the new family *Bathycorpusculaceae* (Figure 2E and Supplementary Figure 7).

Comparative genome analysis of *Bathycorpusculum* MAGs revealed a purely fermentative metabolism based on amino acids with the potential for reductive acetogenesis from H_2_ and CO_2_ (TB1) or possibly methylated compounds (TB2) (Loh et al., 2021) but ruled out the capacity for methanogenesis or alkane oxidation, which had been reported for other lineages of *Bathyarchaeia* (Evans et al., 2019). Until recently, host-associated *Bathyarchaeia* had been detected only in higher termites (Friedrich et al., 2001; Grieco et al., 2013; Shi et al., 2015). However, short-read amplicon libraries of archaea in the intestinal tract of animals documented the presence of *Bathyarchaeia* also in various species of vertebrates (Youngblut et al., 2021; Thomas et al., 2022). Thomas et al. (2022) demonstrated that the short reads from diverse vertebrates cluster in a sister position to representatives of the genus *Bathycorpusculum*. In contrast to the situation in higher termites, where members of the genus *Bathycorpusculum* appear to be part of the autochthonous microbiota (Loh et al., 2021), the vertebrate hosts are only distantly related, which supports the hypothesis that they were acquired independently from either host environment or diet (Youngblut et al., 2021; Thomas et al., 2022). Remarkably, amplicon libraries of cockroaches and millipedes also contain rare phylotypes that fall outside the radiation of TB1 and TB2 from termites in a lineage that also includes a clone from a manure pit (Figure 7 and Supplementary Figure 7).

### *Nitrososphaerales* and other transient microbiota

Members of the order *Nitrososphaerales* were detected in archaeal clone libraries of soil-feeding termites more than 20 years ago (Friedrich et al., 2001). At the time referred to as the “Terrestrial Cluster” of “*Crenarchaeota*,” they were subsequently placed in the candidate phylum “*Thaumarchaeota*” (Brochier-Armanet et al., 2008), which was recently described as *Nitrososphaerota* (Oren and Garrity, 2021). The GTDB taxonomy classifies the class *Nitrososphaeria* in the phylum *Thermoproteota*.

Members of the order *Nitrosophaerales* are aerobic, ammonia-oxidizing archaea and occur in a wide range of marine and terrestrial ecosystems (Pester et al., 2011). In arthropod guts, they were detected in only a few soil or litter-feeding species of all host groups (Figure 8). The phylotypes do not form arthropod-specific lineages but fall into the radiation of uncultured representatives from soils and sediments (Supplementary Figure 8), suggesting that they are part of a transient microbiota taken up from the environment. This is supported by their prevalence in the food soil and midgut of the humivorous larva of *P. ephippiata* or in the food soil and/or nest material of soil-feeding termites (e.g., *Isognathotermes* spp., *Ophiotermes* sp., and *Nitiditermes orthognatus*), where they are abundant also in the anterior gut compartments but not in the hindgut (P1–P4; Supplementary Table 3). This is consistent with the consumption of nest material by soil-feeding termites (Nalepa et al., 2001).

Using the *amoA* gene as a functional marker, ammonia-oxidizing archaea have been detected particularly in the guts of soil-feeding termites and humivorous scarab beetle larvae (Majeed et al., 2014; Miambi et al., 2022). Considering the high ammonia concentrations in the guts of these host groups (Ji and Brune, 2006; Ngugi et al., 2011) and the considerable influx of oxygen into the peripheral regions of all gut compartments (Brune et al., 1995), *Nitrososphaerales* may be metabolically active during gut passage and contribute to the emissions of the greenhouse gas N_2_O by termites and scarab beetle larvae (Ngugi and Brune, 2012; Brauman et al., 2015).

Methanogens of the genera *Methanocella* and *Methanosarcina* were not detected in gut samples of arthropods but in the food soil of *Cubitermes fungifaber* and *P. ephippiata*. The latter were also absent in most gut samples of millipedes but highly abundant in their excreta (Šustr et al., 2014). A representative of the genus *Methanothrix* was detected in the gut of a single sample of *I. fungifaber* (Cu547). Members of these genera are typical for soil and sediment habitats but do not belong to the autochthonous archaeal microbiota in arthropod guts.

### Hydrogenotrophic vs. methylotrophic lineages

Methanogens colonizing the gut of arthropods reduce either CO_2_ or methyl groups to methane using hydrogen as electron donor. The former are hydrogenotrophic methanogens from the orders *Methanobacteriales* and *Methanomicrobiales*; the latter are obligately hydrogen-dependent methyl reducers from the orders *Methanosarcinales* and *Methanomassiliicoccales*. Obligately methyl-reducing Methanosarcinales, represented exclusively by the genera *Methanimicrococcus* and *Methanolapillus* (Thomas et al., 2021; Protasov and Brune, unpublished results), and all members of the order *Methanomassiliicoccales* lack the methyl branch of the Wood–Ljungdahl pathway and are therefore restricted to methylated substrates such as methanol or methylamines (e.g., Borrel et al., 2014; Lang et al., 2015; Söllinger et al., 2016). Members of the genus *Methanosphaera*, which are obligately methyl-reducing methanogens in the intestinal tract of mammals (Miller and Wolin, 1985; Fricke et al., 2006), are absent in arthropods.

The proportion of methylotrophic lineages in the methanogenic communities of arthropod guts differs substantially among host species (Figure 8). While each host family (and subfamily of higher termites) comprises species that harbor only hydrogenotrophic methanogens, all major host groups comprise representatives with a high abundance of methylotrophs. Even among lower termites, which were previously thought to harbor mainly hydrogenotrophic *Methanobacteriales* (reviewed by Brune, 2019), several species are heavily colonized by methylotrophs. Since methyl-reducing methanogens will always outcompete CO_2_-reducing hydrogenotrophs for H_2_, the abundance of methylotrophs in intestinal tracts is most likely regulated by the availability of methyl groups (Feldewert et al., 2020).

Methyl-disproportioning and aceticlastic methanogens are absent in the intestinal tract of arthropods. Although many members of the order *Methanosarcinales* can dismutate methyl groups to methane and CO_2_, their independence from external hydrogen is of little advantage in the intestinal tract of animals, where they are outcompeted by methyl-reducing methanogens owing to their low affinity for methanol and other methylated compounds (Sprenger et al., 2007; Feldewert et al., 2020). The absence of aceticlastic methanogens, however, is more puzzling, since acetate concentrations are much higher in intestinal tracts than in sediments. Although it is generally assumed that the rather slow-growing members of this guild cannot cope with the short residence times of the intestinal contents, it remains enigmatic why they do not avoid washout by attaching to intestinal surfaces or protists (see Brune, 2019).

### Microhabitats in arthropod guts

The intestinal tracts of arthropods are characterized by steep radial gradients of oxygen and hydrogen between gut wall and lumen and strong axial dynamics of these and other physicochemical parameters (Brune, 2014, 2019). Hence, it is not surprising that their archaeal communities are diverse and differ between gut compartments (Friedrich et al., 2001). Hydrogenotrophic *Methanobacteriaceae* of the genera *Methanacia*, *Methanobaculum*, and *Methanoflexus* have been localized on the chitinous lining of the hindgut wall of termites (Leadbetter and Breznak, 1996; Leadbetter et al., 1998). The methyl-reducing *M. blatticola* colonizes the same microhabitat in the hindgut of cockroaches (Sprenger et al., 2000). Although attachment to the hindgut wall is thought to prevent washout, it comes at a cost. Hydrogenotrophs attached to the gut wall are not only severely hydrogen-limited but also exposed to the constant influx of oxygen into this microhabitat (Tholen and Brune, 2000). Both *Methanobaculum cuticularis* and *M. blatticola* actively remove oxygen from their environment (Leadbetter and Breznak, 1996; Sprenger et al., 2007). While *Methanobinarius arboriphilus* and *M. cuticularis* reduce oxygen using H_2_ via a F_420_-dependent oxidase (Seedorf et al., 2004; Tholen et al., 2007), the mechanism employed by *M. blatticola* is unclear.

### Associations with protists

Association with protists prevents washout and allows methanogens to position themselves in the anoxic lumen of the hindgut, where hydrogen supply is also better than at the gut wall (Brune, 2019). Moreover, most anaerobic protists in the hindgut of arthropods possess hydrogenosomes and provide a stable substrate source for their hydrogenotrophic symbionts. Associations between methanogens and protists are also common in sediments and are regarded as mutualistic because of the cross-feeding of H_2_ (Fenchel and Finlay, 2018; Treitli et al., 2023).

Ciliates of the genus *Nyctotherus*, which are found in the gut of cockroaches and millipedes, are commonly colonized by methanogens of the genera *Methanobaculum* and *Methanobinarius*, including *Methanobinarius endosymbioticus* from *Nyctotherus ovalis* (Gijzen et al., 1991; van Hoek et al., 2000; Lind et al., 2018; Supplementary Figure 3). Free-living relatives of these ciliates in sediments, however, are associated with hydrogenotrophic methanogens of the genera *Methanoregula*, *Methanocorpusculum*, *Methanoplanus*, or *Methanobacterium* (Fenchel and Finlay, 2018), suggesting that the endosymbionts are not host-specific but were acquired independently from the pool of methanogens present in the respective environment. This is also the case for rumen ciliates, which are associated with a close relative of *M. ruminantium* (Tokura et al., 1999).

Parabasalid flagellates of lower termites are also frequently associated with methanogens (Odelson and Breznak, 1985; Lee et al., 1987). They were identified as members of the genera *Methanovirga* and *Methanoflexus* (Ohkuma et al., 1995; Tokura et al., 2000; Hara et al., 2004; Inoue et al., 2008; Supplementary Figure 3). Both genera occur exclusively in the gut of lower termites, corroborating that flagellate-associated methanogens have also evolved multiple times from free-living lineages in their respective environment.

### Host specificity and mode of transmission

Although methanogens from arthropods typically form clusters specific for a particular host group, evidence of a co-cladogenesis is not always conclusive. In addition, there are numerous examples of host switching within a given clade (Figure 8). This suggests that the archaeal microbiota in arthropods exhibits a mixed mode of transmission, including both vertical transfer from parents to offspring and environmental exchange (e.g., through predation or co-habitation), as suggested already for the bacterial microbiota of termites (Bourguignon et al., 2018). Environmental transfer would also explain why millipede-associated archaea frequently cluster with those of termites, as millipedes are frequently found in termite nests (Mwabvu, 2005; Choosai et al., 2009).

Many lineages of methanogens present in millipedes, cockroaches, and higher termites are also found in the larva of *P. ephippiata* (Egert et al., 2003). With the exception of the genus *Methanobinarius*, all lineages found in the humivorous larva of *P. ephippiata* are absent in the root-feeding larva of the closely related *Melolontha melolontha* (Egert et al., 2005). An exchange of methanogens between arthropods that occur in the same habitat (e.g., via the feces) seems likely, especially given the close relatedness of the respective phylotypes (Supplementary Figures 3–7).

## Conclusion

Arthropods harbor unique lineages of methanogens from several orders. They comprise both CO_2_-reducing and methyl-reducing hydrogenotrophs. Some lineages (*Methanimicrococcus*, *Methanolapillus*, *Methanorbis*, *Methanomicula*, and *Methanoplasma*) are sister groups of lineages from the intestinal tract of vertebrates, indicating a common evolutionary origin from non-intestinal ancestors, whereas other lineages must have arisen only in arthropods (*Methanofilum*). The deep-branching phylogenies of each host-associated clade (at least at the genus level) indicate that they have coevolved with their intestinal niches over a long period of time since acquisition from the environment. The occurrence of the same lineages in unrelated host groups suggests the presence of similar ecological niches in the gut of methane-emitting arthropods. However, the reason for the absence of methanogens in all other arthropod groups remains unclear.

## Taxonomy

Most archaea from the arthropod guts belong to genus-level lineages that are either unclassified or require reclassification. The presence of both high-quality genomes and 16S rRNA gene sequences for most lineages allow the proposal of new taxa under the *Code of Nomenclature of Prokaryotes Described from Sequence Data* (SeqCode) (Hedlund et al., 2022; Whitman et al., 2022). The new names and new combinations are listed in Table 1, along with the designated nomenclatural type. The authors of previously proposed *Candidatus* names are assigned as descriptors for the corresponding new taxa. The protologues including etymologies and the full descriptions are given in Supplementary Data File 1). The new isolates will be described also under ICNP in separate publications once genome analysis and phenotypic characterization are completed.

**TABLE 1:** New taxa and new combinations of archaea proposed under SeqCode and the designated nomenclatural type.

Taxon and descriptor of new taxa (type genus or species)	New species or combination (type genome)
***Methanobacteriales***	
*Methanocatella* gen. nov. Protasov and Brune (*Methanocatella smithii* comb. nov.)	*Methanocatella smithii* comb. nov. (GCF_000016525)
	*Methanocatella gottschalkii* comb. nov. (GCF_003814835)
	*Methanocatella millerae* comb. nov. (GCF_900103415)
	*Methanocatella oralis* comb. nov. (GCF_001639275)
	*Methanocatella thaueri* comb. nov. (GCF_003111625)
	*Methanocatella woesei* comb. nov. (GCF_003111605)
*Methanarmilla* gen. nov. Protasov and Brune (*Methanarmilla wolinii* comb. nov.)	*Methanarmilla wolinii* comb. nov. (GCF_000621965)
	*Methanarmilla boviskoreani* comb. nov. (GCF_000320505)
*Methanobinarius* gen. nov. Protasov and Brune (*Methanobinarius arboriphilus* comb. nov.*)*	*Methanobinarius arboriphilus* comb. nov. (GCF_002072215)
	*Methanobinarius endosymbioticus* comb. nov. (GCA_003315655)
*Methanobaculum* gen. nov. Protasov and Brune (*Methanobaculum cuticularis* comb. nov.)	*Methanobaculum cuticularis* comb. nov. (GCA_001639285)
*Methanoflexus* gen. nov. Protasov and Brune (*Methanoflexus curvatus* comb. nov.)	*Methanoflexus curvatus* comb. nov. (GCF_001639295)
	*Methanoflexus mossambicus* sp. nov. (GCA_031261915)
*Methanorudis* gen. nov. Protasov and Brune (*Methanorudis spinitermitis* sp. nov.)	*Methanorudis spinitermitis* sp. nov. (GCA_031286225)
*Methanovirga* gen. nov. Protasov and Brune (*Methanovirga basalitermitum* sp. nov.)	*Methanovirga aequatorialis* sp. nov. (GCA_031282205)
	*Methanovirga australis* sp. nov. (GCA_031272765)
	*Methanovirga basalitermitum* sp. nov. (GCA_031284445)
	*Methanovirga meridionalis* sp. nov. (GCA_031289325)
	*Methanovirga procula* sp. nov. (GCA_031280375)
*Methanacia* gen. nov. Protasov and Brune (*Methanacia filiformis* comb. nov.)	*Methanacia filiformis* comb. nov. (GCF_001639265)
***Methanomicrobiales***	
*Methanorbis* gen. nov. Protasov and Brune (*Methanorbis furvi* sp. nov.)	*Methanorbis basalitermitum* sp. nov. (GCA_031287415)
	*Methanorbis furvi* sp. nov. (GCA_032714615)
	*Methanorbis rubei* sp. nov. (GCA_032714495)
*Methanofilum* gen. nov. Protasov and Brune (*Methanofilum arcanum* sp. nov.)	*Methanofilum arcanum* sp. nov. (GCA_031285085)
***Methanosarcinales***	
*Methanimicrococcus* (*Methanimicrococcus blatticola*)	*Methanimicrococcus hacksteinii* sp. nov. (GCA_032714515)
	*Methanimicrococcus hongohii* sp. nov. (GCA_032594095)
	*Methanimicrococcus labiotermitis* sp. nov. (GCA_009784005)
	*Methanimicrococcus odontotermitis* sp. nov. (GCA_031286065)
	*Methanimicrococcus stummii* sp. nov. (GCA_032594435)
*Methanolapillus* gen. nov. Protasov and Brune (*Methanolapillus millepedarum* sp. nov.)	*Methanolapillus africanus* sp. nov. (GCA_032714475)
	*Methanolapillus ohkumae* sp. nov. (GCA_032594355)
	*Methanolapillus millepedarum* sp. nov. (GCA_032594115)
*Methanofrustulum* gen. nov. Protasov and Brune (*Methanofrustulum fimipullorum* sp. nov.)	*Methanofrustulum fimipullorum* sp. nov. (GCA_012518265)
***Methanomassiliicoccales***	
*Methanomethylophilaceae* fam. nov. Gaci et al. (*Methanomethylophilus* gen. nov.)	
*Methanomethylophilus* gen. nov. Borrel et al. (*Methanomethylophilus alvi* sp. nov.)	*Methanomethylophilus alvi* sp. nov. (GCA_000300255)
*Methanarcanum* gen. nov. Chibani et al. (*Methanarcanum hacksteinii* sp. nov.)	*Methanarcanum hacksteinii* sp. nov. (GCA_006954405)
*Methanoprimaticola* gen. nov. Chibani et al. (*Methanoprimaticola hominis* sp. nov.)	*Methanoprimaticola hominis* sp. nov. (GCA_006954465)
*Methanogranum* gen. nov. Iino et al. (*Methanogranum gryphiswaldense* sp. nov.)	*Methanogranum gryphiswaldense* sp. nov. (GCA_019262145)
*Methanoplasma* gen. nov. Lang and Brune (*Methanoplasma termitum* sp. nov.)	*Methanoplasma termitum* sp. nov. (GCF_000800805)
	*Methanoplasma cognatum* sp. nov. (GCA_009777615)
	*Methanoplasma glyptotermitis* (sp. nov. GCA_031267895)
	*Methanoplasma porotermitis* sp. nov. (GCA_031290095)
	*Methanoplasma reticulitermitis* sp. nov. (GCA_031287135)
*Methanomicula* gen. nov. Protasov and Brune (*Methanomicula labiotermitis* sp. nov.)	*Methanomicula labiotermitis* sp. nov. (GCA_009780575)
***Bathyarchaeales***	
*Bathycorpusculaceae* fam. nov. Loh and Brune (*Bathycorpusculum* gen. nov.)	
*Bathycorpusculum* gen. nov. Loh and Brune (*Bathycorpusculum acidaminoxidans* sp. nov.)	*Bathycorpusculum acidaminoxidans* sp. nov. (GCA_009786255)
	*Bathycorpusculum acetigenerans* sp. nov. (GCA_009781675)
	*Bathycorpusculum fermentans* sp. nov. (GCA_009787175)
	*Bathycorpusculum hydrogenotrophicum* sp. nov. (GCA_009783705)
	*Bathycorpusculum grumuli* sp. nov. (GCA_009776805)
	*Bathycorpusculum soli* sp. nov. (GCA_031277345)
	*Bathycorpusculum terrae* sp. nov. (GCA_009784175)
	*Bathycorpusculum termitum* sp. nov. (GCA_031254875)

The protologues including the descriptors of each new taxon, the etymologies of the new taxon names, and the full descriptions of all taxa are given in the Supplementary Data File 1.

## Materials and methods

### Samples and DNA extraction

Termite colonies that were collected in the field were sampled within a week of collection. Samples from termite colonies maintained in other laboratories were processed within a few days after arrival. Species identity was confirmed by comparing their mitochondrial cytochrome oxidase II (COII) gene sequences (Pester and Brune, 2006) with those in public databases. COII gene sequences that were not represented in public databases have been submitted to NCBI GenBank. Cockroaches, beetles, and millipedes were obtained from commercial breeders and dissected within a few days after arrival. Detailed information on all samples is given in Supplementary Table 1.

Specimens were immobilized on ice, decapitated, and dissected with sterile forceps. Whole guts or individual gut sections were pooled and homogenized in phosphate buffer (Köhler et al., 2012; Schauer et al., 2012). The number of specimens included in each sample was adjusted to account for size differences between species (one gut or gut section for millipedes, scarab beetles and cockroaches, 3–10 for termites). DNA for clone libraries was extracted using a bead-beating protocol with subsequent phenol–chloroform purification (Paul et al., 2012). DNA for amplicon libraries was purified using the DNeasy soil kit (Qiagen), which also includes mechanical disruption with zirconium beads, according to the manufacturer’s instructions.

### Clone libraries of 16S rRNA genes

Archaeal 16S rRNA genes were amplified as previously described (Paul et al., 2012), using the archaea-specific forward primer Ar109f (5′-AMDGCTCAGTAACACGT-3′) with either the archaea-specific reverse primer Ar912r (5′-CTCCCCCGCCAATTCCTTTA-3′) or the prokaryote-specific reverse primer 1490R (5′-GGHTACCTTGTTACGACTT-3′). The PCR products were purified using the MinElute PCR Purification Kit (Qiagen) and cloned using the pGEM-T vector kit (Promega). For each library, between 20 and 50 clones with correctly sized inserts were bidirectionally sequenced with M13 primers on a capillary sequencer at GATC-Biotech (Konstanz, Germany).

### Amplicon libraries of 16S rRNA genes for next generation sequencing

Barcoded 16S rRNA amplicons were generated in two rounds of PCR. In the first round, 16S rRNA genes were amplified using primers Ar109F and 1490R (see above) tagged with M13 sequences at the 5′ end (M13F 5′-TGTAAAACGACGGCCAGT-3′; M13R 5′-GGAAACAGCTATGACCATG-3′). A 5′ block (5′-NH_4_-C_6_) was added to each primer to ensure that no untagged amplicons were carried over into the second PCR. In the second round, samples were multiplexed by attaching unique barcodes (16-mers) to each end of the amplicons using bar-coded M13 forward and reverse primers (Pacific Biosciences).

In both rounds, the PCR conditions followed standard PacBio amplicon generation protocols, except that the HiFi Hot Start DNA Polymerase (Roche Life Science) was replaced with Herculase (Agilent). Round 1: initial denaturation step (92°C for 2 min), followed by 35 cycles of denaturation (94°C for 20 s), annealing (52°C for 30 s), and extension (68°C for 45 s), and a final extension step (68°C for 7 min). Round 2: initial denaturation step (95°C for 3 min), followed by 12 cycles of denaturation (95°C for 30 s), annealing (57°C for 30 s), and extension (72°C for 1 min), and a final extension step (72°C for 7 min).

The barcoded amplicons were purified using AMPure PB beads (Beckman Coulter) following the manufacturer’s protocol, pooled at equimolar concentrations, and ligated with SMRTbell adapters following standard PacBio library preparation protocols. The library was sequenced on a Pacific Biosciences Sequel II platform at the Dresden Genome Center (DGC), Dresden, Germany, using one SMRT 8 M cell with the Sequel II Binding Kit 2.1 containing the Sequel Polymerase 2.0 and with a movie length of 600 min. Circular consensus (CCS) reads were generated using the CCS v. 6.4.0 Bioconda package (pbbioconda, Pacific Biosciences) (Grüning et al., 2018).

### Read curation and taxonomic classification PacBio amplicons

Read curation followed the pipeline of Martijn et al. (2017) with adaptations for single 16S rRNA gene sequencing. Briefly, the base-calling confidence of the raw CCS reads was assessed, and sequences with associated read quality scores below 0.99 were removed from the dataset. Sequences were curated using mothur software (Schloss, 2020), first by demultiplexing barcoded amplicons with the trim.seqs command and sorting the samples by host species, followed by removal of primer sequences. Chimerae were removed using the UCHIME package (Edgar et al., 2011) integrated in mothur with our curated reference database (see below). Quality-trimmed reads were clustered into operational taxonomic units (OTUs) at 97% sequence identity level using the VSEARCH tool (Rognes et al., 2016), again using our reference database as a template.

### Phylogenetic analysis of 16S rRNA genes

Sequences were imported into the ARB-SILVA database (v. 138^1^) using the *ARB* software package (v. 7.0^2^), aligned with the SINA Aligner (v1.2.12) (Ludwig et al., 2004; Pruesse et al., 2012; Yilmaz et al., 2014), and placed into the phylogenetic framework of the guide tree using the ARB parsimony tool. The overall alignment was manually improved using the alignment editor integrated in ARB, taking into account secondary structures. Multiple sequence alignments comprising representative sequences of different archaeal orders or classes were exported with appropriate outgroup sequences. Maximum-likelihood trees were inferred using IQ-TREE 2 with the GTR + I + G4 substitution model suggested by the ModelFinder tool (Kalyaanamoorthy et al., 2017; Minh et al., 2020). Node support was assessed using the Shimodaira–Hasegawa approximate-likelihood ratio test (SH-aLRT) and ultrafast bootstrap analysis (Guindon et al., 2010; Hoang et al., 2018).

### The Dictyopteran Gut Microbiota Reference Database (DictDb)

In this study, our in-house 16S rRNA reference database was expanded to include archaeal sequences from both host-associated and environmental samples. The current iteration of the Dictyopteran Gut Microbiota Reference Database (DictDb v. 5.1 Archaea) was built upon the framework of the latest release (v. 138.1) of the Silva 16S rRNA database (Quast et al., 2013) and includes only archaea. An extension of the previously published DictDb v. 3.0 (Mikaelyan et al., 2015), which covers only bacterial sequences, will be introduced in an upcoming publication. The curated database was further enriched with near-full-length 16S rRNA sequences from studies targeting archaeal diversity in arthropod guts, both from our research group and from the literature. These include the 16S rRNA gene sequences of our MAGs and metagenomes from termite guts (Hervé et al., 2020), and representative sequences obtained in the present study. We also included the curated 16S rRNA sequences provided by SBDI Sativa (Lundin and Andersson, 2021) to establish robust links to the GTDB taxonomy and the genome-based phylogenies. The curated taxon-specific trees of DictDb were used as sources for the reference alignment and taxonomy files for the analysis of next generation sequencing data with mothur (see above). The database was further enriched by adding sequences that are not included in the reference trees (mostly shorter sequences from this and previous studies) using the parsimony tool implemented in Arb.

### Genome sequencing

High-molecular-weight DNA of pure cultures was isolated with the DNAEasy Blood & Tissue Kit (Qiagen) following the manufacturer’s protocol. The quality of isolated DNA was first checked by agarose gel electrophoresis and validated using an Agilent Bioanalyzer 2100 and the Agilent DNA 12000 kit as recommended by the manufacturer (Agilent Technologies, Waldbronn, Germany). The concentration and purity of the isolated DNA was first estimated with a Nanodrop ND-1000 instrument (PeqLab Erlangen, Germany), and the exact concentration was determined using the Qubit^®^ dsDNA HS Assay kit as recommended by the manufacturer (Life Technologies GmbH, Darmstadt, Germany). Illumina sequencing libraries were prepared using the Nextera XT DNA Sample Preparation kit. To assess the quality and size of the libraries, samples were run on an Agilent Bioanalyzer 2100 using the Agilent High Sensitivity DNA kit according to the manufacturer’s instructions. DNA concentration of the libraries was determined using the Qubit^®^ dsDNA HS Assay kit (Life Technologies GmbH). The libraries were sequenced using a MiSeq system and the reagent kit v3 with 600 cycles as recommended by the manufacturer (Illumina, San Diego, CA, USA). Quality control and quality-filtering of the generated Illumina reads were performed with FastQC v0.11.5 (Andrews, 2010) and Trimmomatic v0.39 (Bolger et al., 2014) using default parameters, respectively. Genomes were assembled with the SPAdes genome assembler software v3.15.2 with default parameters (Bankevich et al., 2012). The quality of the *de novo* assembly was validated using Qualimap v2.2.1 (García-Alcalde et al., 2012).

The genomes of strains Hf6, Ac7, Am2, and Es2 were additionally sequenced using Nanopore technology. Libraries were prepared with 1.5 μg high-molecular-weight DNA using the Ligation Sequencing lit 1D (SQK-LSK109) and the Native Barcode Expansion kit (EXP-NBD104 and EXP-NBD114) as recommended by the manufacturer (Oxford Nanopore Technologies). Libraries were sequenced for 72 h using a MinION device Mk1B and a SpotON Flow Cell R9.4.1 (Oxford Nanopore Technologies). Basecalling and demultiplexing was done with the MinKNOW software and Guppy in high accuracy mode. The generated reads were quality filtered using fastp v0.23.2 (Chen et al., 2018), and the remaining adapters were removed using porechop v0.2.4.^3^ Hybrids were assembled using Unicycler v0.5.0 with default settings.

Genes were predicted and the assembled genomes were annotated using Prokka v1.14.5 (Seemann, 2014) with default settings.

### Phylogenomic analysis

Genomes were classified using the GTDB toolkit (GTDB-Tk v2.3.0) with GTDB release 214 as reference (Chaumeil et al., 2022). The alignment of 53 archaeal marker genes generated by the GTDB toolkit was used to infer a phylogenomic tree with IQ-TREE 2 under the LG + F + I + G4 model suggested by modelfinder. Branch support was assessed by ultrafast bootstrap approximation (1,000 replicates). For rank normalization, RED values were calculated from the annotated tree according to Parks et al. (2018) using PhyloRank (v. 1.12^4^). The archaeal phylogenomics tree (Figure 1) was rendered using iTOL v. 6.8 and edited in Inkscape v. 1.0.1 (Letunic and Bork, 2021).

## Data availability statement

Newly obtained representative OTU sequences were submitted to NCBI GenBank under the accession numbers OP851801–OP852117; OQ724653–OQ724818; OR354372–OR354382, and OR451225–OR451228. Clone library sequences from this study were submitted under the accession numbers OP713915–OP714075 and OR449907–OR449908. Binned small subunit (SSU) sequences extracted from MAGs were submitted under the accession numbers OQ730111–OQ730154; OR140526–OR140534, and OR359878–OR359882. The accession numbers of the new isolates and MAGs are listed in Supplementary Table 2. The Dictyopteran gut reference database (DictDb v. 5.1 Archaea) as Arb file and the accompanying mothur reference files are available on GitHub: https://github.com/brunelab/databases/.

## Ethics statement

The manuscript presents research on animals that do not require ethical approval for their study.

## Author contributions

EP: Conceptualization, Resources, Investigation, Data curation, Formal analysis, Validation, Visualization, Writing – original draft, Writing – review and editing. JON: Conceptualization, Resources, Investigation, Data curation, Formal analysis, Validation, Visualization, Writing – original draft. JMKS: Conceptualization, Methodology, Investigation, Data curation, Formal analysis, Validation, Visualization, Writing – review and editing. USM: Data curation, Formal analysis, Visualization, Writing – review and editing. VH: Data curation, Formal analysis, Visualization, Writing – review and editing. CD: Data curation, Formal analysis, Visualization, Writing – review and editing. KL: Investigation, Formal analysis, Writing – review and editing. LM: Investigation, Formal analysis, Writing – review and editing. KP: Methodology, Investigation, Formal analysis, Writing – review and editing. AP: Methodology, Data curation, Writing – review and editing. TK-R: Data curation, Formal analysis, Writing – review and editing. EM: Resources, Investigation, Formal analysis, Writing – review and editing. HIB: Resources, Investigation, Formal analysis, Writing – review and editing. CF: Resources, Writing – review and editing. DKN: Resources, Writing – review and editing. RP: Resources, Writing – review and editing. DS-D: Resources, Writing – review and editing. JŠ: Resources, Writing – review and editing. RD: Resources, Methodology, Writing – review and editing. AB: Conceptualization, Funding acquisition, Project administration, Supervision, Resources, Data curation, Formal analysis, Validation, Visualization, Writing – original draft, Writing – review and editing.

## Footnotes

1
http://www.arb-silva.de

2
http://www.arb-home.de

3
https://github.com/rrwick/Porechop

4
https://github.com/dparks1134/PhyloRank
