# Supplementary material for: Diversity and taxonomic revision of methanogens and other archaea in the intestinal tract of terrestrial arthropods
Source: Front Microbiol. 2023 Nov 15;14:1281628. doi: 10.3389/fmicb.2023.1281628 (PMC10684969; doi:10.3389/fmicb.2023.1281628)
Supplement: Supplementary file 1 [file Data_Sheet_1.pdf]

Supplementary Data File 1

Protologues for the new archaeal taxa

Description of new taxa and new combinations of archaea proposed under SeqCode.

Methanobacteriales Balch and Wolfe 1981

Methanobacteriaceae Barker 1956

Methanocatella gen. nov. Protasov and Brune

Etymology: Me.tha.no.ca.tel'la. N.L. neut. n. *methanum*, methane; N.L. pref. *methano-*, pertaining to methane; L. fem. n. *catella*, an ornamental chain; N.L. fem. n. *Methanocatella*, a methane-producing chain, referring to the short chains of cells characteristic of this genus.

Description: Short oval rods or coccobacilli, 0.4–1 µm in width and 0.6–1.5 µm in length. Cells occur singly, in pairs or in chains of 4–6 cells. Gram positive. Nonmotile. Require complex medium with yeast extract, trypticase, rumen fluid, or fecal extract. Optimum temperature is 35–42 °C. Use H_2 + CO_2 as substrates for methanogenesis, some species grow poorly on formate. Members of the genus are defined by phylogenomic analysis as a monophyletic group that shows a relative evolutionary divergence (RED) similar to that of the neighboring genera.

Type species: *Methanocatella smithii* comb. nov.

Methanocatella gottschalkii (Miller and Lin 2002) comb. nov. Protasov and Brune

Etymology: gott.schalk'i.i. N.L. gen. masc. n. *gottschalkii*, of Gottschalk, named in honor of Gerhard Gottschalk for his notable contributions to the understanding of the biochemistry of methanogenesis.

Basonym: *Methanobrevibacter gottschalkii* Miller and Lin 2002

Description: The species description remains the same as in Miller and Lin (2002). Includes all genomes that show ≥95% average nucleotide identity (ANI) to the genome of the type strain. The type strain has a genome size of 1.87 Mbp and a G+C content of 30.0 mol%.

Type strain: HO^{T} (ATCC BAA-1169^{T}; DSM 11977^{T}) isolated from horse feces.

Type genome: GCF_003814835^{TS}; U55238 (16S rRNA).

***Methanocatella millerae* (Rea et al. 2007) comb. nov. Protasov and Brune**

Etymology: mil'ler.ae. N.L. gen. fem. n. *millerae*, of Miller, named after Terry L. Miller for her contributions to the taxonomy of methanogens, in particular the genus *Methanobrevibacter.*

Basonym: *Methanobrevibacter millerae* Rea et al. 2007

Description: The species description remains the same as in Rea et al. (2007). Includes all genomes that show ≥95% average nucleotide identity (ANI) to the genome of the type strain. The type strain has a genome size of 2.72 Mbp and a G+C content of 36.5 mol%.

Type strain: ZA-10^T (DSM 16643^T) isolated from cow rumen.

Type genome: GCF_900103415^{TS}; AY196673 (16S rRNA).

***Methanocatella oralis* (Ferrari et al. 1995) comb. nov. Protasov and Brune**

Etymology: o.ra'lis. L. neut. n. *os* (gen. *oris*), mouth; L. masc./fem. adj. suff. -*alis*, suffix denoting pertaining to; L. fem. adj. *oralis*, pertaining to the mouth.

Basonym: *Methanobrevibacter oralis* Ferrari et al. 1995

Description: The species description remains the same as in Miller (2015). Includes all genomes that show ≥95% average nucleotide identity (ANI) to the genome of the type strain. The type strain has a genome size of 2.14 Mbp and a G+C content of 27.7 mol%.

Type strain: ZR^T (DSM 7256^T; JCM 30027^T) isolated from the human oral cavity.

Type genome: GCF_001639275^{TS}; HE654003 (16S rRNA).

***Methanocatella smithii* (Balch and Wolfe 1981) comb. nov. Protasov and Brune**

Etymology: smith'i.i. N.L. gen. masc. n. *smithii*, of Smith, named after P.H. Smith, who isolated the type strain.

Basonym: *Methanobrevibacter smithii* Balch and Wolfe 1981

Description: The species description remains the same as in Miller (2015). Includes all genomes that show ≥95% average nucleotide identity (ANI) to the genome of the type strain. The type strain has a genome size of 1.85 Mbp and a G+C content of 31.0 mol%.

Type strain: PS^T (DSM 861^T; JCM 30028) isolated from sewage digester.

Type genome: GCF_000016525^{TS}; U55233 (16S rRNA).

***Methanocatella thaueri* (Miller and Lin 2002) comb. nov. Protasov and Brune**

Etymology: thau'er.i. N.L. gen. masc. n. *thaueri*, of Thauer, named in honor of Rudolf K. Thauer for his fundamental contributions to the delineation of the biochemistry of methanogenesis.

Basonym: *Methanobrevibacter thaueri* Miller and Lin 2002

Description: The species description remains the same as in Miller and Lin (2002). Includes all genomes that show ≥95% average nucleotide identity (ANI) to the genome of the type strain. The type strain has a genome size of 2.24 Mbp and a G+C content of 36.9 mol%.

Type strain: CW^T (DSM 11955^T) isolated from cow feces.

Type genome: GCF_003111625^{TS}; U55236 (16S rRNA).

Methanocatella woesei (Miller and Lin 2002) comb. nov. Protasov and Brune

Etymology: woe'se.i. N.L. gen. masc. n. *woesei*, of Woese, named in honor of Carl R. Woese for his pioneering contributions to the understanding of the phylogeny of methanogens and other microorganisms.

Basonym: *Methanobrevibacter woesei* Miller and Lin 2002

Description: The species description remains the same as in Miller and Lin (2002). Includes all genomes that show ≥95% average nucleotide identity (ANI) to the genome of the type strain. The type strain has a genome size of 1.54 Mbp and a G+C content of 29.9 mol%.

Type strain: GS^{T} (DSM 11979^{T}) isolated from goose feces.

Type genome: GCF_003111605^{TS}; U55237 (16S rRNA).

Methanarmilla gen. nov. Protasov and Brune

Etymology: Me.than.ar.mil'la. N.L. neut. n. *methanum*, methane; L. fem. n. *armilla*, bracelet; N.L. fem. n. *Methanarmilla* (no connecting vowel because second stem starts with a vowel), methane-producing bracelet, referring to the short chains of cells formed by the type species.

Description: Short oval rods or coccobacilli, 0.6 µm in width and 1–1.8 µm in length. Cells occur singly, in pairs or in short chains. Gram positive. Nonmotile. Require complex medium with acetate, yeast extract, trypticase, volatile fatty acids, coenzyme M. Optimum temperature is 37–40° C. Use H_2 + CO_2 or formate + CO_2 as substrates for methanogenesis. Members of the genus are defined by phylogenomic analysis as a monophyletic group that shows a relative evolutionary divergence (RED) similar to that of the neighboring genera.

Type species: *Methanarmilla wolinii* comb. nov.

Methanarmilla boviskoreani (Lee et al. 2013) comb. nov. Protasov and Brune

Etymology: bo.vis.ko.re.a'ni. L. masc./fem. n. *bos* (gen. *bovis*), cattle; N.L. adj. *koreanus*, Korean; N.L. gen. masc./fem. n. *boviskoreani*, of Korean cattle.

Basonym: *Methanobrevibacter boviskoreani* Lee et al. 2013

Description: The species description remains the same as in Lee et al. (2013). Includes all genomes that show ≥95% average nucleotide identity (ANI) to the genome of the type strain. The type strain has a genome size of 2.04 Mbp and a G+C content of 28.9 mol%.

Type strain: JH-1^{T} (DSM 25824^{T}; JCM 18376^{T}; KCTC 4102^{T}) isolated from rumen of Korean native cattle (*Bos taurus coreanae*).

Type genome: GCF_000320505^{TS}; KC608769 (16S rRNA).

Methanarmilla wolinii (Miller and Lin 2002) comb. nov. Protasov and Brune

Etymology: wo.lin'i.i. N.L. gen. masc. n. *wolinii*, of Wolin, named in honor of Meyer J. Wolin for his singular contributions to the physiological understanding of the role of methanogens and interspecies hydrogen transfer in anaerobic habitats.

Basonym: *Methanobrevibacter wolinii* Miller and Lin 2002

Description: The species description remains the same as in Miller and Lin (2002). Includes all genomes that show ≥95% average nucleotide identity (ANI) to the genome of the type strain. The type strain has a genome size of 2.04 Mbp and a G+C content of 24.2 mol%.

Type strain: SH^T (ATCC BAA-1170^T; DSM 11976^T) isolated from sheep feces.

Type genome: GCF_000621965^{TS}; U55240 (16S rRNA).

Methanobinarius gen. nov. Protasov and Brune

Etymology: Me.tha.no.bi.na'ri.us. N.L. neut. n. *methanum*, methane; N.L. pref. *methano-*, pertaining to methane; L. masc. adj. *bīnārius*, consisting of two things; N.L. masc. n. *Methanobinarius*, methane-producing (organism) consisting of two things, referring to the pairs of cells formed by the type species.

Description: Short oval rods that occur singly, in pairs or in short chains. Gram positive. Nonmotile. Use H_2 + CO_2 as substrates for methanogenesis, might also grow poorly on formate. Grow poorly on mineral medium with vitamins; yeast extract, casamino acids, and rumen fluid strongly stimulate growth. Optimum temperature is 30–37° C. Members of the genus are defined by phylogenomic analysis as a monophyletic group that shows a relative evolutionary divergence (RED) similar to that of the neighboring genera.

Type species: *Methanobinarius arboriphilus* comb. nov.

Methanobinarius arboriphilus (Zeikus and Henning 1975) comb. nov. Protasov and Brune

Etymology: ar.bo.ri'phi.lus. L. fem. n. *arbor* (gen. *arboris*), tree; N.L. masc. adj. suff. *-philus*, friend, loving; from Gr. masc. adj. *philos*; N.L. masc. adj. *arboriphilus*, tree-loving.

Basonym: *Methanobrevibacter arboriphilus* corrig. (Zeikus and Henning 1975) Balch and Wolfe.

Homotypic synonyms: *Methanobrevibacter arboriphilicus* [sic] Balch and Wolfe 1981, 216; *Methanobacterium arbophilicum* Zeikus and Henning 1975.

Description: The species description remains the same as in Miller (2015). Includes all genomes that show ≥95% average nucleotide identity (ANI) to the genome of the type strain. The type strain has a genome size of 2.44 Mbp and a G+C content of 25.4 mol%.

Type strain: DH1^T (ATCC 33747^T; DSM 1125^T; JCM 13429^T) isolated from the wetwood core of a tree.

Type genome: GCF_002072215^{TS}; AY196665 (16S rRNA).

Methanobinarius endosymbioticus sp. nov. Protasov and Brune

Etymology: en.do.sym.bi.o'ti.cus. Gr. pref. *endo-*, within; N.L. masc. adj. *symbioticus*, from Greek *biōtikos*, living together; N.L. masc. adj. *endosymbioticus*, living together within (another organism).

Description: Species identified by metagenomic analyses. Includes all genomes that show ≥95% average nucleotide identity (ANI) to the type genome. The G+C content of the type genome is 25.2 mol%, and the estimated genome size is 1.91 Mbp.

Type genome: NOE^{TS} = GCA_003315655^{TS} from the ciliate *Nyctotherus ovalis* colonizing the gut of the cockroach *Blaptica dubia*.

Methanobaculum gen. nov. Protasov and Brune

Etymology: Me.tha.no.ba'cu.lum. N.L. neut. n. *methanum*, methane; N.L. pref. *methano-*, pertaining to methane; L. neut. n. *baculum*, small rod; N.L. neut. n. *Methanobaculum*, a small methane-producing rod.

Description: Straight short rods with slightly tapered ends that occur singly, in pairs, or in short chains. Non-motile. Gram-positive. Strict anaerobe. Use H_2 + CO_2 as substrates for methanogenesis, growth on formate is poor. Grow poorly on mineral medium with vitamins. Yeast extract, casamino acids, and rumen fluid strongly stimulate growth. Optimum temperature is 30–37° C. Members of the genus are defined by phylogenomic analysis as a monophyletic group that shows a relative evolutionary divergence (RED) similar to that of the neighboring genera.

Type species: *Methanobaculum cuticularis* comb. nov.

Methanobaculum cuticularis (Leadbetter and Breznak 1997) comb. nov. Protasov and Brune

Etymology: cu.ti.cu.la'ris. L. dim. fem. n. *cuticula*, skin; N.L. masc. adj. *cuticularis*, referring to the cuticular surface of the termite hindgut epithelium, which is colonized by this organism.

Basonym: *Methanobrevibacter cuticularis* Leadbetter and Breznak 1997

Description: The species description remains the same as in Miller (2015). Includes all genomes that show ≥95% average nucleotide identity (ANI) to the type genome. The type strain has a genome size of 2.60 Mbp and a G+C content of 26.7 mol%.

Type strain: RFM-1^T (DSM 11139^T) isolated from the termite *Reticulitermes flavipes*.

Type genome: GCA_001639285^{TS}; U41095 (16S rRNA).

Methanoflexus gen. nov. Protasov and Brune

Etymology: Me.tha.no.fle'xus. N.L. neut. n. *methanum*, methane; N.L. pref. *methano-*, pertaining to methane; L. masc. n. *flexus*, a bending, turning, winding; N.L. masc. n. *Methanoflexus*, methane-producing (organism) with a curved shape.

Description: Curved rods that occur singly, in pairs, or in chains. Gram positive. Nonmotile. Strict anaerobe. Require yeast extract or rumen fluid for growth. Optimum temperature is 30° C. Use H_2 + CO_2 as substrates for methanogenesis. Members of the genus are defined by phylogenomic analysis as a monophyletic group that shows a relative evolutionary divergence (RED) similar to that of the neighboring genera.

Type species: *Methanoflexus curvatus* comb. nov.

Methanoflexus curvatus (Leadbetter and Breznak 1997) comb. nov. Protasov and Brune

Basonym: *Methanobrevibacter curvatus* Leadbetter and Breznak 1997

Etymology: cur.va'tus. L. v. *curvo*, to bend; L. part. adj. *curvatus*, bent, curved; referring to the shape of the cell.

Description: The species description remains the same as in Miller (2015). Includes all genomes that show ≥95% average nucleotide identity (ANI) to the type genome. The type strain has a genome size of 2.41 Mbp and a G+C content of 25.7 mol%.

Type strain: RFM-2^{T} (DSM 11111^{T}) isolated from the termite *Reticulitermes flavipes*.

Type genome: GCF_001639295^{TS}; U62533 (16S rRNA).

Methanoflexus mossambicus **sp. nov. Protasov and Brune**

Etymology: mos.sam.bi'cus. N.L. masc. adj. *mossambicus*, of Mozambique; denoting the origin of the host.

Description: Species identified by metagenomic analyses. Includes all genomes that show ≥95% average nucleotide identity (ANI) to the type genome. The G+C content of the type genome is 24.0 mol%, and the estimated genome size is 3.25 Mbp.

Type genome: Hm464_bin70^{TS} = GCA_031261915^{TS} from the termite *Hodotermes mossambicus*.

Methanorudis **gen. nov. Protasov and Brune**

Etymology: Me.tha.no.ru'dis. N.L. neut. n. *methanum*, methane; N.L. pref. *methano-*, pertaining to methane; L. f. n. *rudis*, a small stick; N.L. fem. n. *Methanorudis*, a small methane-producing stick.

Description: Genus identified by metagenomic analyses. Members of the genus are defined by phylogenomic analysis as a monophyletic group that shows a relative evolutionary divergence (RED) similar to that of the neighboring genera.

Type species: *Methanorudis spinitermitis* sp. nov.

Methanorudis spinitermitis **sp. nov. Protasov and Brune**

Etymology: spi.ni.ter'mi.tis. N.L. masc. n. *Spinitermes*, a genus of termites; N.L. gen. sg. masc. n. *spinitermitis*, of *Spinitermes* (the host genus).

Description: Species identified by metagenomic analyses. Includes all genomes that show ≥95% average nucleotide identity (ANI) to the type genome. The G+C content of the type genome is 25.8 mol%, and the estimated genome size is 1.96 Mbp.

Type genome: Spi319_bin4^{TS} = GCA_031286225^{TS} from the termite *Spinitermes trispinosus*; OQ730145 (16S rRNA).

Methanovirga **gen. nov. Protasov and Brune**

Etymology: Me.tha.no.vir'ga. N.L. neut. n. *methanum*, methane; N.L. pref. *methano-*, pertaining to methane; L. fem. n. *virga*, a rod, twig; N.L. fem. n. *Methanovirga*, a methane-producing rod.

Description: Genus identified by metagenomic analyses. Members of the genus are defined by phylogenomic analysis as a monophyletic group that shows a relative evolutionary divergence (RED) similar to that of the neighboring genera.

Type species: *Methanovirga basalitermitum* sp. nov.

Methanovirga aequatorialis **sp. nov. Protasov and Brune**

Etymology: ae.qua.to.ri.a'lis. M.L. masc./fem. adj. *aequatorialis*, equatorial; denoting the origin of the host.

Description: Species identified by metagenomic analyses. Includes all genomes that show ≥95% average nucleotide identity (ANI) to the type genome. The G+C content of the type genome is 29.3 mol%, and the genome size is 2.05 Mbp.

Type genome: Nm470_bin38^{TS} = GCA_031282205^{TS} from the termite *Neotermes meruensis*; OQ730131 (16S rRNA).

Methanovirga australis **sp. nov. Protasov and Brune**

Etymology: aus.tra'lis. L. masc./fem. adj. *australis*, southern; denoting the origin of the host from the southern hemisphere (Australia).

Description: Species identified by metagenomic analyses. Includes all genomes that show ≥95% average nucleotide identity (ANI) to the type genome. The G+C content of the type genome is 26.8 mol%, and the genome size is 2.50 Mbp.

Type genome: Md513_bin122^{TS} = GCA_031272765^{TS} from the termite *Mastotermes darwinensis*; OR354382 (16S rRNA).

Methanovirga basalitermitum **sp. nov. Protasov and Brune**

Etymology: ba.sa.li.ter'mi.tum. L. adj. *basalis*, basal, L. masc. n. *termes,* a woodworm, a termite; N.L. gen. pl. masc. n. *basalitermitum*, of lower (basal) termites.

Description: Species identified by metagenomic analyses. Includes all genomes that show ≥95% average nucleotide identity (ANI) to the type genome. The G+C content of the type genome is 27.8 mol%, and the genome size is 2.18 Mbp.

Type genome: Ncb351_bin114^{TS} = GCA_031284445^{TS} from the termite *Neotermes cubanus*; OQ730130 (16S rRNA).

Methanovirga meridionalis **sp. nov. Protasov and Brune**

Etymology: me.ri.di.o.na'lis. L. masc./fem. adj. *meridionalis*, southern; denoting the origin of the host from the southern hemisphere (Chile).

Description: Species identified by metagenomic analyses. Includes all genomes that show ≥95% average nucleotide identity (ANI) to the type genome. The G+C content of the type genome is 23.7 mol%, and the genome size is 2.39 Mbp.

Type genome: Pq454_bin35^{TS} = GCA_031289325^{TS} from the termite *Porotermes quadricollis*; OP852022 (16S rRNA).

Methanovirga procula **sp. nov. Protasov and Brune**

Etymology: procula. L. fem. adj. *procula*, from far away; denoting the origin from a remote island (Réunion).

Description: Species identified by metagenomic analyses. Includes all genomes that show ≥95% average nucleotide identity (ANI) to the type genome. The G+C content of the type genome is 26.7 mol%, and the genome size is 2.38 Mbp.

Type genome: Pc512_bin29^{TS} = GCA_031280375^{TS} from the termite *Prorhinotermes canalifrons*.

Methanacia gen. nov. Protasov and Brune

Etymology: Me.than.a'ci.a. N.L. neut. n. *methanum*, methane; L. fem. n. *acia*, thread, yarn; N.L. neut. n. *Methanacia* (no connecting vowel because second stem starts with a vowel), a methane-producing thread.

Description: Filamentous rods with slightly tapered ends. Gram positive. Nonmotile. Strict anaerobe. Require yeast extract for growth. Optimum temperature is 30 °C. Use H_2 + CO_2 as substrates for methanogenesis. Members of the genus are defined by phylogenomic analysis as a monophyletic group that shows a relative evolutionary divergence (RED) similar to that of the neighboring genera.

Type species: *Methanacia filiformis* comb. nov.

Methanacia filiformis (Leadbetter et al. 1998) comb. nov. Protasov and Brune

Basonym: *Methanobrevibacter filiformis* Leadbetter et al. 1998

Etymology: fi.li.for'mis. L. neut. n. *filum*, a thread; L. masc./fem. adj. suff. *-formis*, -like, in the shape of; from L. fem. n. *forma*, figure, shape, appearance; N.L. masc. adj. *filiformis*, thread-shaped.

Description: The species description remains the same as in Miller (2015). Includes all genomes that show ≥95% average nucleotide identity (ANI) to the type genome. The type strain has a genome size of 2.60 Mbp and a G+C content of 26.9 mol%.

Type strain: RFM-3^T (DSM 11501^T) isolated from the termite *Reticulitermes flavipes*.

Type genome: GCF_001639265^{TS}; U82322 (16S rRNA).

Methanomicrobiales Balch and Wolfe 1981

Methanocorpusculaceae Zellner et al. 1989

Methanorbis gen. nov. Protasov and Brune

Etymology: Me.than.or'bis. N.L. neut. n. *methanum*, methane; L. masc. n. *orbis*, a disk; N.L. masc. n. *Methanorbis* (no connecting vowel because second stem starts with a vowel), a methane-producing disk.

Description: Cells are ovoid cocci, 1-3 µm. Cells occur singly. Nonmotile. Require complex medium with yeast extract, peptone, and acetate. Growth occur in temperature interval between 25-42° C. H_2 and CO_2 are the substrates for methanogenesis; formate can substitute H_2. It includes all genomes that were placed into that genus based on relative evolutionary divergence (RED) to the type species.

Type species: *Methanorbis furvi* sp. nov.

Methanorbis basalitermitum sp. nov. Protasov and Brune

Etymology: ba.sa.li.ter'mi.tum. L. neut. adj. *basalis*, basal, L. masc. n. *termes*, a woodworm, a termite; N.L. gen. pl. masc. n. *basalitermitum*, of lower (basal) termites.

Description: Species identified by metagenomic analyses. It includes all genomes that show ≥95% average nucleotide identity (ANI) to the genome of the type strain. The G+C content of the type genome is 50.9 mol%, and the estimated genome size is 1.27 Mbp.

Type genome: Rs511_bin27^{TS} = GCA_031287415^{TS} from the termite *Reticulitermes flavipes*.

Methanorbis furvi sp. nov. Protasov and Brune

Etymology: fur'vi. L. masc. n. *furvus*, the black one; L. gen. n. *furvi*, of the black one, referring to the color of the host.

Description: Ovoid cocci cells 1–3 µm. Non-motile. H_2 and CO_2 are the substrates for methanogenesis; formate can substitute H_2. Acetate and formate required for growth. Optimum temperature is 33° C. It includes all genomes that show ≥95% average nucleotide identity (ANI) to the type genome. The G+C content of the type genome is 50.1 mol%, and the genome size is 1.84 Mbp.

Type strain: Ag1^{T} from the millipede *Archispirostreptus gigas*, DSMZ 115764^{T}, JCM ####^{T} (pending).

Type genome: GCA_032714615^{TS}; OQ442338 (16S rRNA).

Methanorbis rubei sp. nov. Protasov and Brune

Etymology: ru'be.i. L. masc. n. *rubeus*, the red one; L. gen. masc. n. *rubei*, of the red one, referring to the color of the host.

Description: Ovoid cocci cells 1–3 µm. Non-motile. H_2 and CO_2 are the substrates for methanogenesis; formate can substitute H_2. Yeast extract stimulates growth. Optimum temperature is 33° C. It includes all genomes that show ≥95% average nucleotide identity (ANI) to the type genome. The G+C content of the type genome is 50.2 mol%, and the genome size is 1.82 Mbp.

Type strain: Cs1^{T} from the millipede *Centrobolus splendidus*, DSMZ 115765^{T}, JCM ####^{T} (pending).

Type genome: GCA_032714495^{TS}; OQ442335 (16S rRNA).

Methanospirillaceae Boone et al. 2002

Methanofilum gen. nov. Protasov and Brune

Etymology: Me.tha.no.fi'lum. N.L. neut. n. *methanum*, methane; N.L. pref. *methano-*, pertaining to methane; L. neut. n. *filum*, a thread or string; N.L. neut. n. *Methanofilum*, a methane-producing thread.

Description: Genus identified by metagenomic analyses. Members of the genus are defined by phylogenomic analysis as a monophyletic group that shows a relative evolutionary divergence (RED) similar to that of the neighboring genera.

Type species: *Methanofilum arcanum* sp. nov.

Methanofilum arcanum sp. nov. Protasov and Brune

Etymology: ar.ca'num. L. neut. adj. *arcanum*, hidden mysterious, referring to the fact that members of this genus have so far escaped enrichment and isolation.

Description: Species identified by metagenomic analyses. Includes all genomes that show ≥95% average nucleotide identity (ANI) to the type genome. The G+C content of the type genome is 41.0 mol%, and the genome size is 1.83 Mbp.

Type genome: Xx448_bin9^{TS} = GCA_031285085^{TS} from the termite *Dicuspiditermes spinitibialis*; OQ730147 (16S rRNA).

Methanomassiliicoccales Iino et al. 2013

Methanomethylophilaceae fam. nov. Gaci et al.

Synonym: "*Candidatus* Methanomethylophilaceae" Gaci *et al.* 2014 (not validly published)

Etymology: Me.tha.no.me.thy.lo.phi.la'ce.ae. N.L. neutr. n. *Methanomethylophilus*, type genus of the family; L. fem. pl. n. suff. *-aceae*, ending to denote a family; N.L. fem. pl. n. *Methanomethylophilaceae*, the *Methanomethylophilaceae* family of the genus *Methanomethylophilus.*

Description: Family identified by metagenomic analyses. Members of the family are defined by phylogenomic analysis as a monophyletic group that shows a relative evolutionary divergence (RED) similar to that of the neighboring families.

Type genus: *Methanomethylophilus* gen. nov.

Methanomethylophilus gen. nov. Borrel *et al.*

Synonym: "*Candidatus* Methanomethylophilus " Borrel *et al.* 2012 (not validly published)

Etymology: Me.tha.no.me.thy.lo'phi.lus. N.L. neut. n. *methanum*, methane; N.L. pref. *methano-*, pertaining to methane; N.L. neut. n. *methylum*, the methyl group; N.L. pref. *methylo-*, pertaining to the methyl group; N.L. masc. adj. suff. *-philus* (from Gr. masc. adj. *philos*), loving; N.L. masc. n. *Methanomethylophilus*, methane-producing organism loving methyl groups.

Description: Strictly anaerobic and chemoheterotrophic cocci. Utilize methanol and mono, di- and trimethylamines only in combination with H_2 as substrates for methanogenesis. The genus is defined by phylogenomic analysis as a monophyletic group that shows a relative evolutionary divergence (RED) similar to that of the neighboring genera.

Type species: *Methanomethylophilus alvi* sp. nov.

Methanomethylophilus alvi sp. nov. Borrel *et al.*

Etymology: al'vi. L. gen. masc. n. *alvi*, of the bowel

Synonym: "*Candidatus* Methanomethylophilus alvus" Borrel *et al.* 2012 (not validly published)

Description: Utilize methanol only in combination with H_2 as substrates for methanogenesis. Methanol potentially could be substituted by mono-, di-, or trimethylamines. Includes all genomes that fall into a relative evolutionary divergence

(RED) range of the type species of the genus. The G+C content of the type genome is 55.6 mol%, and the genome size is 1.66 Mbp.

Type genome: Mx1201^{TS} = GCA_000300255^{TS} from the human gut; KC412010 (16S rRNA).

Methanarcanum gen. nov. Chibani et al.

Etymology: Me.tha.no.ar'ca.num. N.L. neut. n. *methanum*, methane; L. masc. adj. *arcanus*, silent, secret; N.L. neut. n. *Methanarcanum* (no connecting vowel because second stem starts with a vowel), an archaeon forming methane in a puzzling way.

Synonym: "*Candidatus* Methanarcanum" Chibani *et al.* 2022 (not validly published)

Description: Genus identified by metagenomic analyses. Includes all genomes that were placed into that genus based on relative evolutionary divergence (RED) to the type species.

Type species: *Methanarcanum hacksteinii* sp. nov.

Methanarcanum hacksteinii sp. nov. Chibani et al.

Etymology: hack.stei'ni.i. N.L. gen. n. hacksteinii named after Johannes H. P. Hackstein.

Synonym: "*Candidatus* Methanarcanum hacksteinii" Chibani *et al.* 2022 (not validly published)

Description: Species identified by metagenomic analyses. Includes all genomes that show ≥95% average nucleotide identity (ANI) to the type genome. The G+C content of the type genome is 47.6%, and the estimated genome size is 1.41 Mbp.

Type genome: Mx02^{TS} = GCA_006954405^{TS} from human stool.

Methanoprimaticola gen. nov. Chibani et al.

Etymology: Me.tha.no.pri.ma.ti'co.la. N.L. neut. n. *methanum*, methane; N.L. pref. *methano-*, pertaining to methane; N.L. pl. n. *Primates,* a zoological order; L. suff. *-cola* (from L. masc. or fem. n. *incola*) an inhabitant, dweller; N.L. fem. n. *Methanoprimaticola* a methane-forming dweller of primates.

Synonym: "*Candidatus* Methanoprimaticola" Chibani *et al.* 2022 (not validly published)

Description: Genus identified by metagenomic analyses. Includes all genomes that were placed into that genus based on relative evolutionary divergence (RED) to the type species.

Type species: *Methanoprimaticola hominis* sp. nov.

Methanoprimaticola hominis sp. nov. Chibani et al.

Etymology: ho'mi.nis. L. gen. n. *hominis*, of a human.

Synonym: "*Candidatus* Methanoprimaticola hominis" Chibani *et al.* 2022 (not validly published)

Description: Species identified by metagenomic analyses. Includes all genomes that show ≥95% average nucleotide identity (ANI) to the type genome. The G+C content of the type genome is 58.25%, and the estimated genome size is 1.49 Mbp.

Type genome: Mx06^{TS} = GCA_006954465^{TS} from human stool.

Methanogranum gen. nov. Iino *et al.*

Synonym: "*Candidatus* Methanogranum" Iino *et al.* 2013 (not validly published)

Etymology: Me.tha.no.gra'num. N.L. neut. n. *methanum*, methane; N.L. pref. *methano-*, pertaining to methane; L. neut. n. *granum*, grain, seed; N.L. neut. n. *Methanogranum*, a methane-producing grain-like organism.

Description: Strictly anaerobic and chemoheterotrophic. Cells are cocci occurring singly. Utilize methanol only in combination with H_2 as substrates for methanogenesis. The genus is defined by phylogenomic analysis as a monophyletic group that shows a relative evolutionary divergence (RED) similar to that of the neighboring genera.

Type species: *Methanogranum gryphiswaldense* sp. nov.

Methanogranum gryphiswaldense sp. nov. Weil et al.

Etymology: gry.phis.wald.en'se. N.L. neut. adj. *gryphiswaldense*, pertaining to the town Greifswald.

Description: Includes all genomes that fall into a relative evolutionary divergence (RED) range of the type species of the genus. The G+C content of the type genome is 43.7 mol%, and the genome size is 1.51 Mbp.

Type genome: U3.2.1^{TS} = GCA_019262145^{TS} from peat soil; CP076745: 301404-302874 (16S rRNA).

Methanoplasma gen. nov. Lang and Brune

Etymology: Me.tha.no.plas'ma. N.L. neut. n. *methanum*, methane; N.L. pref. *methano-*, pertaining to methane; Gr. neut. n. *plasma*, something formed or molded, figure, image; N.L. neut. n. *Methanoplasma*, a methane-producing form.

Description: Strictly anaerobic and chemoheterotrophic. Cells are cocci occurring singly. Cells does not have cell wall. No characteristic fluorescence of methanogens under UV light due to lack of F_{420}. Utilize methanol or methylamines only in combination with H_2 as substrates for methanogenesis. The genus is defined by phylogenomic analysis as a monophyletic group that shows a relative evolutionary divergence (RED) similar to that of the neighboring genera.

Type species: *Methanoplasma termitum* sp. nov.

Methanoplasma cognatum sp. nov. Protasov and Brune

Etymology: cog.na'tum. L. neutr. adj. *cognatum*, related by blood, sibling (of *Methanoplasma termitum*).

Description: Species identified by metagenomic analyses. Includes all genomes that show ≥95% average nucleotide identity (ANI) to the type genome. The G+C content of the type genome is 52.2 mol%, and the estimated genome size is 1.61 Mbp.

Type genome: Th196P3_bin2^{TS} = GCA_009777615^{TS} from the termite *Termes hospes*; OQ730120 (16S rRNA).

Methanoplasma glyptotermitis sp. nov. Protasov and Brune

Etymology: gly.pto.ter'mi.tis. N.L. gen. sg. n. *glyptotermitis*, referring to *Glyptotermes*, the host genus.

Description: Species identified by metagenomic analyses. Includes all genomes that show ≥95% average nucleotide identity (ANI) to the type genome. The G+C content of the type genome is 49.7 mol%, and the estimated genome size is 1.84 Mbp.

Type genome: Gsp477_bin30^{TS} = GCA_031267895^{TS} from the termite *Glyptotermes* sp.; OQ730127 (16S rRNA).

Methanoplasma porotermitis sp. nov. Protasov and Brune

Etymology: po.ro.ter'mi.tis. N.L. gen. sg. n. *porotermitis*, referring to *Porotermes*, the host genus.

Description: Species identified by metagenomic analyses. Includes all genomes that show ≥95% average nucleotide identity (ANI) to the type genome. The G+C content of the type genome is 57.3 mol%, and the estimated genome size is 1.69 Mbp.

Type genome: Po218_bin46^{TS} = GCA_031290095^{TS} from the termite *Porotermes adamsoni*; OQ730132 (16S rRNA).

Methanoplasma reticulitermitis sp. nov. Protasov and Brune

Etymology: re.ti.cu.li.ter'mi.tis. N.L. gen. sg. n. *reticulitermitis*, referring to *Reticulitermes*, the host genus.

Description: Species identified by metagenomic analyses. Includes all genomes that show ≥95% average nucleotide identity (ANI) to the type genome. The G+C content of the type genome is 53.4 mol%, and the estimated genome size is 1.34 Mbp.

Type genome: Rs511_bin65^{TS} = GCA_031287135^{TS} from the termite *Reticulitermes flavipes*; OQ730134 (16S rRNA).

Methanoplasma termitum sp. nov. Lang and Brune

Synonym: "*Candidatus* Methanoplasma termitum" Lang et al. 2015 (not validly published)

Etymology: ter'mi.tum. L. masc. n. *termes*, a woodworm, a termite, L. masc. n. gen. pl. *termitum*, of termites.

Description: Cells are cocci with a diameter of 0.5–0.8 µm. No cell wall. No autofluorescence under UV light due to lack of cofactor F_{420}. Utilize only H_2 + methanol or H_2 + monomethylamine as substrates for methanogenesis. Require yeast extract, coenzyme M, and vitamins for growth. Species identified by physiological and genomic analyses of a highly enriched culture. Includes all genomes that show ≥95% average nucleotide identity (ANI) to the type genome. The G+C content of the type genome is 49.2 mol%, and the genome size is 1.48 Mbp.

Type genome: MpT1^{TS} = GCF_000800805^{TS} from the termite *Isognathotermes* (*Cubitermes*) *ugandensis*; CP010070 (16S rRNA).

Methanomicula gen. nov. Protasov and Brune

Etymology: Me.tha.no.mi'cu.la. N.L. neut. n. *methanum*, methane; N.L. pref. *methano-*, pertaining to methane; L. fem. n. *micula*, morsel, crumb (of salt); N.L. fem. n. *Methanomicula*, a methane-producing crumb.

Description: Genus identified by metagenomic analyses. Includes all genomes that were placed into that genus based on relative evolutionary divergence (RED) to the type species.

Type species: *Methanomicula labiotermitis* sp. nov.

Methanomicula labiotermitis sp. nov. Protasov and Brune

Etymology: la.bi.o.ter'mi.tis. N.L. gen. sg. n. *labiotermitis*, referring to *Labiotermes*, the host genus.

Description: Species identified by metagenomic analyses. Includes all genomes that show ≥95% average nucleotide identity (ANI) to the type genome. The G+C content of the type genome is 49.7 mol%, and the estimated genome size is 1.44 Mbp.

Type genome: Lab288P1_bin114^{TS} = GCA_009780575^{TS} from the termite *Labiotermes labralis*; OQ724736 (16S rRNA).

Methanosarcinales Boone et al. 2002

Methanosarcinaceae Balch and Wolfe 1981

Methanimicrococcus corrig. Sprenger et al. 2000

Etymology: Me.tha.ni.mic'ro.co.cus. N.L. neut. n. *methanum*, methane; Gr. masc. adj. *mikros*, small, little; N.L. masc. n. *coccus*, coccus; from Gr. masc. n. *kokkos*, grain, seed; M.L. masc. n. *Methanimicrococcus*, a small methane-forming coccus.

Description: Cells are irregular cocci, 1–2 µm. Cells occur singly or sometimes in aggregates. Nonmotile. Require complex medium with yeast extract, peptone, and casamino acids. Growth occurs in a temperature interval between 25-42° C. H_2 and methanol are the substrates for methanogenesis; methylamines can substitute methanol. It includes all genomes that were placed into that genus based on relative evolutionary divergence (RED) to the type species.

Methanimicrococcus hacksteinii sp. nov. Protasov and Brune

Etymology: hack.stein'i.i. N.L. gen. masc. n. *hacksteinii*, of Hackstein, named in honor of Johannes H.P. Hackstein for his important contributions on the hydrogenosomes of anaerobic protists and methanogenesis in arthropod guts.

Description: Irregular cocci cells 1–2 µm in diameter, non-motile. Use methanol and methylamines as methanogenesis substrate together with hydrogen. Acetate and rumen fluid are highly stimulatory for growth. Optimum temperature is 37° C. It includes all genomes that show ≥95% average nucleotide identity (ANI) to the type genome. The G+C content of the type genome is 42.9 mol%, and the estimated genome size is 2.04 Mbp.

Type strain: At1^{T} from the cockroach *Archimandrita tesselata*, DSMZ 115570^{T}, JCM 39383^{T}; OQ442333 (16S rRNA).

Type genome: GCA_032714515^{TS}; OQ442340 (16S rRNA).

***Methanimicrococcus hongohii* sp. nov. Protasov and Brune**

Etymology: hon.goh'i.i. N.L. gen. masc. n. *hongohii*, of Hongoh, named after Yuichi Hongoh in recognition of his important contributions to arthropod gut microbiology.

Description: Irregular cocci cells 1–2 µm in diameter, non-motile. Use methanol and methylamines as methanogenesis substrate together with hydrogen. Acetate, formate, and rumen fluid highly stimulatory for growth. Optimum temperature is 37° C. It includes all genomes that show ≥95% average nucleotide identity (ANI) to the type genome. The G+C content of the type genome is 41.0 mol%, and the genome size is 2.19 Mbp.

Type strain: Hf6^{T} from the cockroach *Henschoutedenia flexivitta*, DSMZ 114388^{T}, JCM 39385^{T}.

Type genome: GCA_032594095^{TS}; OQ442340 (16S rRNA).

***Methanimicrococcus labiotermitis* sp. nov. Protasov and Brune**

Etymology: la.bi.o.ter'mi.tis. N.L. gen. sg. n. *labiotermitis*, referring to *Labiotermes*, the host genus.

Description: Species identified by metagenomic analyses. Includes all genomes that show ≥95% average nucleotide identity (ANI) to the type genome. The G+C content of the type genome is 45.5 mol%, and the estimated genome size is 1.83 Mbp.

Type genome: Lab288P3_bin112^{TS} = GCA_009784005^{TS} from the termite *Labiotermes labralis*; OQ724779 (16S rRNA).

***Methanimicrococcus odontotermitis* sp. nov. Protasov and Brune**

Etymology: o.don.to.ter'mi.tis. N.L. gen. sg. masc. n. *odontotermitis*, referring to *Odontotermes*, the host genus.

Description: Species identified by metagenomic analyses. Includes all genomes that show ≥95% average nucleotide identity (ANI) to the type genome. The G+C content of the type genome is 43.8 mol%, and the estimated genome size is 1.88 Mbp.

Type genome: TD116_bin10^{TS} = GCA_031286065^{TS} from the termite *Odontotermes* sp.; OR140534 (16S rRNA).

***Methanimicrococcus stummii* sp. nov. Protasov and Brune**

Etymology: stumm'i.i. N.L. gen. masc. n. *stummii*, of Stumm, named in honor of Claudius K. Stumm for his important contributions on the symbiosis of methanogens with anaerobic protists.

Description: Irregular cocci cells 1–2 µm in diameter, non-motile. Use methanol and methylamines as methanogenesis substrate together with hydrogen. Acetate, formate, yeast extract, and rumen fluid are highly stimulatory for growth. Optimum temperature is 37° C. It includes all genomes that show ≥95% average nucleotide identity (ANI) to the type genome. The G+C content of the type genome is 43.1 mol%, and the estimated genome size is 1.82 Mbp.

Type strain: Es2^{T} from the cockroach *Eublaberus serranus*, DSMZ 114387^{T}, JCM 39384^{T}.

Type genome: GCA_032594435^{TS}; OQ442332 (16S rRNA).

Methanolapillus gen. nov. Protasov and Brune

Etymology: Me.tha.no.la'pil.lus. N.L. neut. n. *methanum*, methane; N.L. pref. *methano-*, pertaining to methane; L. masc. n. *lapillus*, a pebble, gem, jewel; N.L. masc. n. *Methanolapillus*, a methane-producing jewel.

Description: Cells are irregular cocci, 1-2 µm. Cells occur singly or sometimes in aggregates. Nonmotile. Require complex medium with yeast extract, peptone, and casamino acids. Grow between 25-42° C. H_2 and methanol are the substrates for methanogenesis; methylamines can substitute methanol. Includes all genomes that were placed into that genus based on relative evolutionary divergence (RED) to the type species.

Type species: *Methanolapillus millepedarum* sp. nov.

Type genome: GCA_032594115^{TS}; OQ442341 (16S rRNA).

Methanolapillus africanus sp. nov. Protasov and Brune

Ethymology: a.fri.ca'nus. L. masc. adj. *africanus*, African.

Description: Irregular coccoid cells, 1–2 µm in diameter, non-motile. Use methanol and methylamines as methanogenesis substrate together with hydrogen. Yeast extract is required for growth; acetate and rumen fluid are highly stimulatory. Optimum temperature is 37° C. It includes all genomes that show ≥95% average nucleotide identity (ANI) to the type genome. The G+C content of the type genome is 44.4 mol%, and the estimated genome size is 2.10 Mbp.

Type strain: Ag5^{T} from the millipede *Archispirostreptus gigas*, DSMZ 115569^{T}, JCM ####^{T} (pending).

Type genome: GCA_032714475^{TS}; OQ442339 (16S rRNA).

Methanolapillus ohkumae sp. nov. Protasov and Brune

Etymology: oh.ku'mae. N.L. gen. masc. n. *ohkumae*, of Ohkuma, named after Moriya Ohkuma in recognition of his important contributions to arthropod gut microbiology.

Description: Irregular cocci cells 1–2 µm in diameter, non-motile. Use methanol and methylamines as methanogenesis substrate together with hydrogen. Formate is required for growth; acetate and rumen fluid are highly stimulatory. Optimum temperature is 37° C. It includes all genomes that show ≥95% average nucleotide identity (ANI) to the type genome. The G+C content of the type genome is 41.0 mol%, and the estimated genome size is 1.89 Mbp.

Type strain: Am2^{T} from the millipede *Anadenobolus monilicornis*, DSMZ 114424^{T}, JCM ####^{T} (pending).

Type genome: GCA_032594355^{TS}; OQ442334 (16S rRNA).

Methanolapillus millepedarum sp. nov. Protasov and Brune

Etymology: (mil.le.pe.da'rum. L. fem. n. *millepeda,* a millipede; gen. pl. n. *millepedarum*, of millipedes).

Description: Irregular cocci cells 1–2 µm in diameter, non-motile. Use methanol and methylamines as methanogenesis substrate together with hydrogen. Acetate, yeast extract, and coenzyme M are required for growth. Optimum temperature is 37° C. It includes all genomes that show ≥95% average nucleotide identity (ANI) to the type genome. The G+C content of the type genome is 42.9 mol%, and the estimated genome size is 1.96 Mbp.

Type strain: Ac7^{T} from the millipede *Atopochetus caudulanus*, DSMZ 114425^{T}, JCM ####^{T} (pending).

Type genome: GCA_032594115^{TS}; OQ442341 (16S rRNA).

Methanofrustulum gen. nov. Protasov and Brune

Etymology: Me.tha.no.frus'tu.lum. N.L. neut. n. *methanum*, methane; N.L. pref. *methano-*, pertaining to methane; L. neutr. n. *frustum*, dim. *frustulum*, morsel, crumb (of food); N.L. neutr. n. *Methanofrustulum*, a methane-producing crumb.

Description: Genus defined by phylogenomic analysis of metagenome-assembled genomes. Members of the genus form a monophyletic group that shows a relative evolutionary divergence (RED) similar to that of the neighboring genera.

Type species: *Methanofrustulum fimipullorum* sp. nov.

Methanofrustulum fimipullorum sp. nov. Protasov and Brune

Etymology: fi.mi.pul.lo'rum. L. neut. n. *fimus*, dung, excrement; L. masc. n. *pullus*, chicken; N.L. gen. pl. masc. n. *fimipullorum*, of chicken dung; denoting the putative origin from chicken manure.

Description: Species defined by metagenome-assembled genomes. Includes all genomes that show ≥95% average nucleotide identity (ANI) to the type genome. The G+C content of the type genome is 42.9 mol%, and the estimated genome size is 1.93 Mbp.

Type genome: AS23ysBPME_4^{TS} = GCA_012518265^{TS} from an anaerobic digester inoculated with chicken manure.

Bathyarchaeales Khomyakova *et al.* 2023

Bathycorpusculaceae fam. nov. Loh and Brune

Etymology: Ba.thy.corp.pus.cu.la'ce.ae. N.L. neutr. n. *Bathycorpusculum*, type genus of the family; L. fem. pl. n. suff. -aceae, ending to denote a family; N.L. fem. pl. n. *Bathycorpusculaceae*, the *Bathycorpusculum* family.

Type genus: *Bathycorpusculum* gen. nov.

Bathycorpusculum gen. nov. Loh and Brune

Etymology: Ba.thy.cor.pus'cu.lum. Gr. neutr. n. *bathos*, depth, profundity; Gr. prefix *bathy-*, deep, especially deep sea; L. neut. n. corpusculum, a little body, a particle; N.L. neut. n. *Bathycorpusculum*, a particle from the deep.

Description: Genus defined by phylogenomic analysis of metagenome-assembled genomes. Putative mixotrophic CO_2-reducing acetogens that use H_2 and amino acids as electron donors. Members of the genus form a monophyletic group that shows a relative evolutionary divergence (RED) similar to that of the neighboring genera.

Type species: *Bathycorpusculum acidaminoxidans* sp. nov.

***Bathycorpusculum acidaminoxidans* sp. nov. Loh and Brune**

Etymology: a.cid.am.in.ox'i.dans. N.L. neut. n. *acidum aminum*, amino acid; N.L. v. *oxido*, make acid, oxidize; N.L. part. adj. *acidaminoxidans*, amino acid-oxidizing.

Description: Species identified by metagenomic analyses. Includes all genomes that show ≥95% average nucleotide identity (ANI) to the type genome. The G+C content of the type genome is 37.9 mol%, and the estimated genome size is 1.99 Mbp.

Type genome: Co191P4_bin18^{TS} = GCA_009786255^{TS} from the litter-feeding termite Cornitermes pugnax; OQ730153 (16S rRNA).

***Bathycorpusculum acetigenerans* sp. nov. Loh and Brune**

Etymology: a.ce.ti.ge'ne.rans. L. neut. n. *acetum*, vinegar; L. part. adj. *generans* (from L. v. *genero*), producing; N.L. neut. n. *acetigenerans*, vinegar-producing.

Description: Species identified by metagenomic analyses. Includes all genomes that show ≥95% average nucleotide identity (ANI) to the type genome. The G+C content of the type genome is 43.4mol%, and the estimated genome size is 2.15Mbp.

Type genome: Emb289P1_bin127^{TS} = GCA_009781675^{TS} from the termite *Embiratermes neotenicus*; OQ724653 (16S rRNA).

***Bathycorpusculum fermentans* sp. nov. Loh and Brune**

Etymology: fer.men'tans. L. part. adj. *fermentans*, fermenting.

Description: Species identified by metagenomic analyses. Includes all genomes that show ≥95% average nucleotide identity (ANI) to the type genome. The G+C content of the type genome is 37.8 mol%, and the estimated genome size is 1.86 Mbp.

Type genome: Nc150P4_bin1^{TS} = GCA_009787175^{TS} from the termite *Nasutitermes corniger*; OQ730148 (16S rRNA).

***Bathycorpusculum grumuli* sp. nov. Loh and Brune**

Etymology: gru'mu.li**.** L. masc. n. *grumulus***,** (dim. of *grumus*)**,** a little heap of earth; L. gen. L. gen. masc. n. *grumuli*, of a little heap of earth; indicates the humivorous nature of its host.

Description: Species identified by metagenomic analyses. Includes all genomes that show ≥95% average nucleotide identity (ANI) to the type genome. The G+C content of the type genome is 35.5 mol%, and the estimated genome size is 2.30 Mbp.

Type genome: Th196P4_bin19^{TS} = GCA_009776805^{TS} from termite *Termes hospes*; OQ730150 (16S rRNA).

***Bathycorpusculum hydrogenotrophicum* sp. nov. Loh and Brune**

Etymology: hyd.ro.ge.no.tro.phi'cum. N.L. neut. n. *hydrogenum*, hydrogen; G. masc. adj. *trophikos*, pertaining to food; N.L. neutr. adj. *hydrogenotrophicum*, feeding on hydrogen.

Description: Species identified by metagenomic analyses. Includes all genomes that show ≥95% average nucleotide identity (ANI) to the type genome. The G+C content of the type genome is 43.2 mol%, and the estimated genome size is 2.24 Mbp.

Type genome: Lab288P3_bin169^{TS} = GCA_009783705^{TS} from the termite *Labiotermes labralis*; OR140533 (16S rRNA).

***Bathycorpusculum soli* sp. nov. Protasov and Brune**

Etymology: so'li. L. neut. n. *solum*, ground, soil; L. gen. neut. n. *soli*, of soil; referring to the habitat of the host.

Description: Species identified by metagenomic analyses. Includes all genomes that show ≥95% average nucleotide identity (ANI) to the type genome. The G+C content of the type genome is 37.5 mol%, and the estimated genome size is 1.88 Mbp.

Type genome: Pmx449_bin19^{TS} = GCA_031277345^{TS} from termite *Promirotermes* sp.; OQ730144 (16S rRNA).

***Bathycorpusculum terrae* sp. nov. Loh and Brune**

Etymology: ter'rae. L. fem. n. *terra*, earth, soil; L. gen. fem. n. *terrae*, of soil; referring to the habitat of the host.

Description: Species identified by metagenomic analyses. Includes all genomes that show ≥95% average nucleotide identity (ANI) to the type genome. The G+C content of the type genome is 38.1 mol%, and the estimated genome size is 1.27 Mbp.

Type genome: Lab288P3_bin115^{TS} = GCA_009784175^{TS} from termite *Labiotermes labralis*; OQ724660 (16S rRNA).

***Bathycorpusculum termitum* sp. nov. Protasov and Brune**

Etymology: ter'mi.tum. L. masc. n. *termes*, a woodworm, a termite; N.L. gen. pl. masc. n. *termitum*, of termites, referring to the host species.

Description: Species identified by metagenomic analyses. Includes all genomes that show ≥95% average nucleotide identity (ANI) to the type genome. The G+C content of the type genome is 43.8 mol%, and the estimated genome size is 2.30 Mbp.

Type genome: Cus372_bin2^{TS} = GCA_031254875^{TS} from the termite *Polyspathotermes* (*Cubitermes*) *sulcifrons*; OQ730139 (16S rRNA).
