# Supplementary material for: Diversity and taxonomic revision of methanogens and other archaea in the intestinal tract of terrestrial arthropods
Source: Front Microbiol. 2023 Nov 15;14:1281628. doi: 10.3389/fmicb.2023.1281628 (PMC10684969; doi:10.3389/fmicb.2023.1281628)
Supplement: Supplementary file 2 [file Data_Sheet_2.pdf]

Supplementary Data File 2

Supplementary Figures

Figure S1. Phylogeny of archaeal genomes, including isolates and MAGs from arthropod guts analyzed in this study (Table S2). The phylogenomic tree is based on an alignment of 53 archaeal marker genes generated using the GTDB toolkit (GTDB-Tk v2.3.0) with GTDB release 214 as reference and was reconstructed with IQ-TREE 2 under the LG+F+I+G4 model of evolution. Branch support was assessed by ultrafast bootstrap approximation (1,000 replicates). A rank-normalized version of this tree is shown in Fig. 2 of the main manuscript. Selected low-quality MAGs (LQ) were included to provide additional context.

Figure S2. Phylogenomic analysis of the family *Methanocorpusculaceae*, including novel genomes of isolates and MAGs from Volmer et al. (2023). Isolates and MAGs from arthopod guts are in bold. For further information, see legend of Fig. S1.

Figure S3. Expanded version of the phylogenetic tree of *Methanobacteriaceae* (Fig. 3 of the main manuscript). Shorter sequences were added using the parsimony tool implemented in Arb. The maximum-likelihood tree is based on a curated alignment of near-full-length 16S rRNA genes (>1,400 sites) and was generated using IQ-TREE with the model GTR+I+G4. Node support was tested with ultrafast bootstrap analysis (● ≥ 95% and ○ ≥ 70%; 1,000 replicates). The scale bar indicates the number of substitutions per site. Sequences from arthropod guts are color-coded (millipedes, green, cockroaches, orange; lower termites, light blue; higher termites, dark blue; beetles; red).

Figure S4. Expanded version of the phylogenetic tree of *Methanomicrobiales* (Fig. 4 of the main manuscript). Shorter sequences were added using the parsimony tool implemented in Arb. For more details, see the legend of Fig. S3.

Figure S5. Expanded version of the phylogenetic tree of *Methanosarcinaceae* (Fig. 5 of the main manuscript). Shorter sequences were added using the parsimony tool implemented in Arb. For more details, see the legend of Fig. S3.

Figure S6. Expanded version of the phylogenetic tree of *Methanomethylophilaceae* (Fig. 6 of the main manuscript). Shorter sequences were added using the parsimony tool implemented in Arb. For more details, see the legend of Fig. S3.

Figure S7. Expanded version of the phylogenetic tree of *Bathycorpusculaceae* (Fig. 7 of the main manuscript). Shorter sequences were added using the parsimony tool implemented in Arb. For more details, see the legend of Fig. S3.

Figure S8. Phylogenetic tree of *Nitrososphaeraceae*, illustrating the position of the sequences from arthropods obtained in this and previous studies. Other *Nitrososphaerales* were used as an outgroup. Shorter sequences were added using the parsimony tool implemented in Arb. For more details, see the legend of Fig. S3.

Figure S1

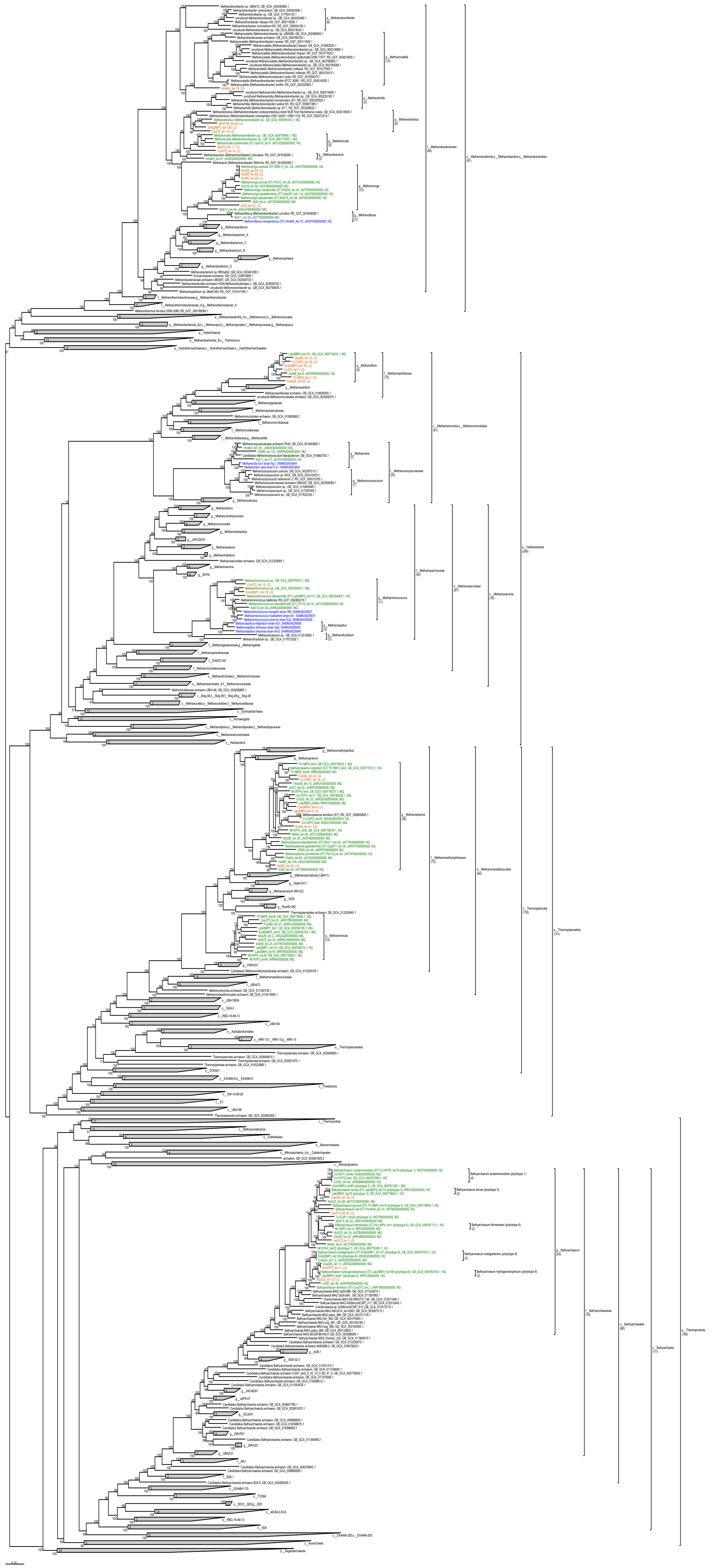

Figure S2

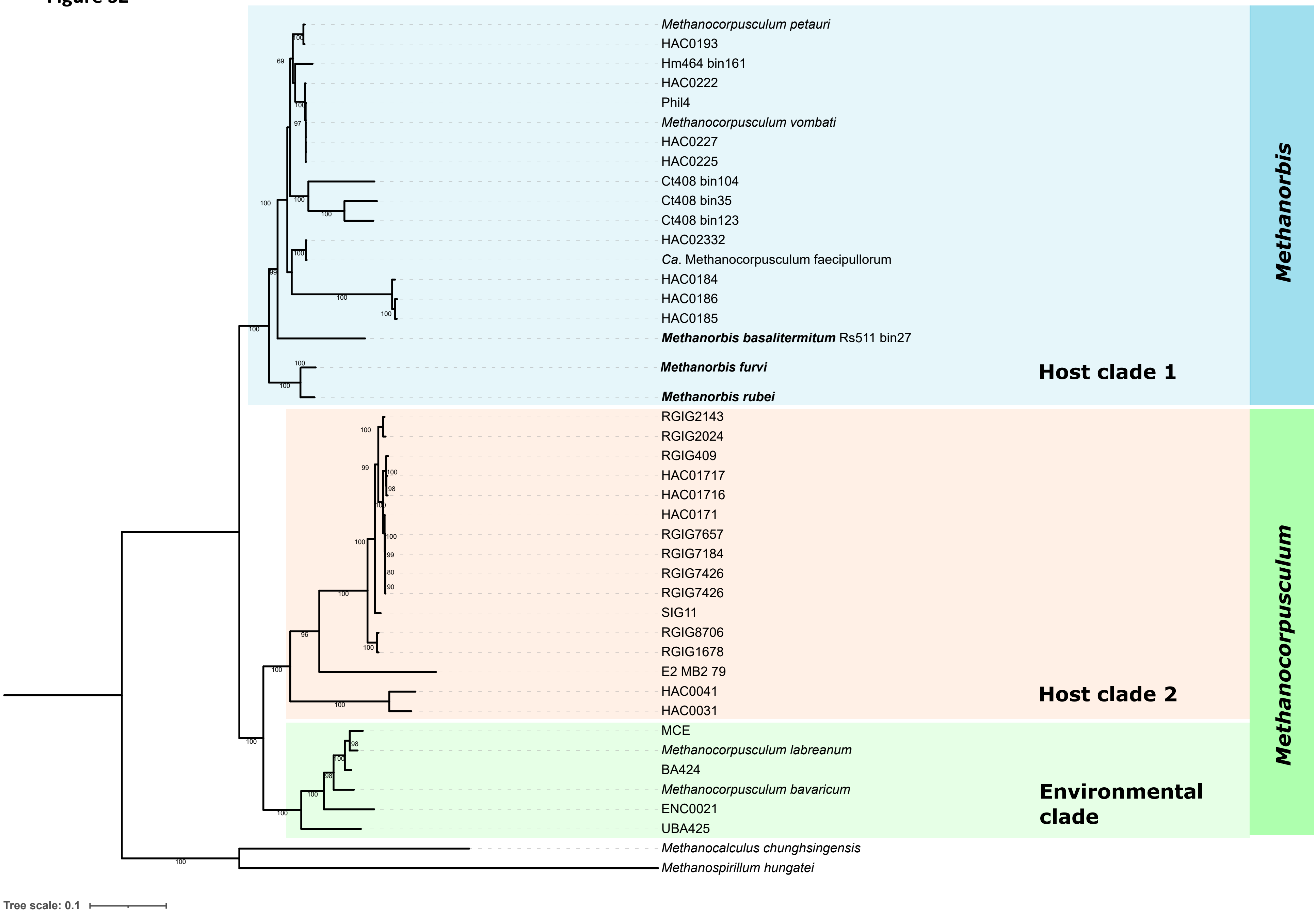
Methanocorpusculum petauri
HAC0193
Hm464 bin161
HAC0222
Phil4
Methanocorpusculum vombati
HAC0227
HAC0225
Ct408 bin104
Ct408 bin35
Ct408 bin123
HAC02332
Ca. Methanocorpusculum faecipullorum
HAC0184
HAC0186
HAC0185
Methanorbis basalitermitum Rs511 bin27
Methanorbis furvi
Methanorbis rubei
Host clade 1
Methanorbis
RGIG2143
RGIG2024
RGIG409
HAC01717
HAC01716
HAC0171
RGIG7657
RGIG7184
RGIG7426
RGIG7426
SIG11
RGIG8706
RGIG1678
E2 MB2 79
HAC0041
HAC0031
Host clade 2
MCE
Methanocorpusculum labreanum
BA424
Methanocorpusculum bavaricum
ENC0021
UBA425
Environmental clade
Methanocorpusculum
Methanocalculus chunghsingensis
Methanospirillum hungatei
Tree scale: 0.1

Figure S3

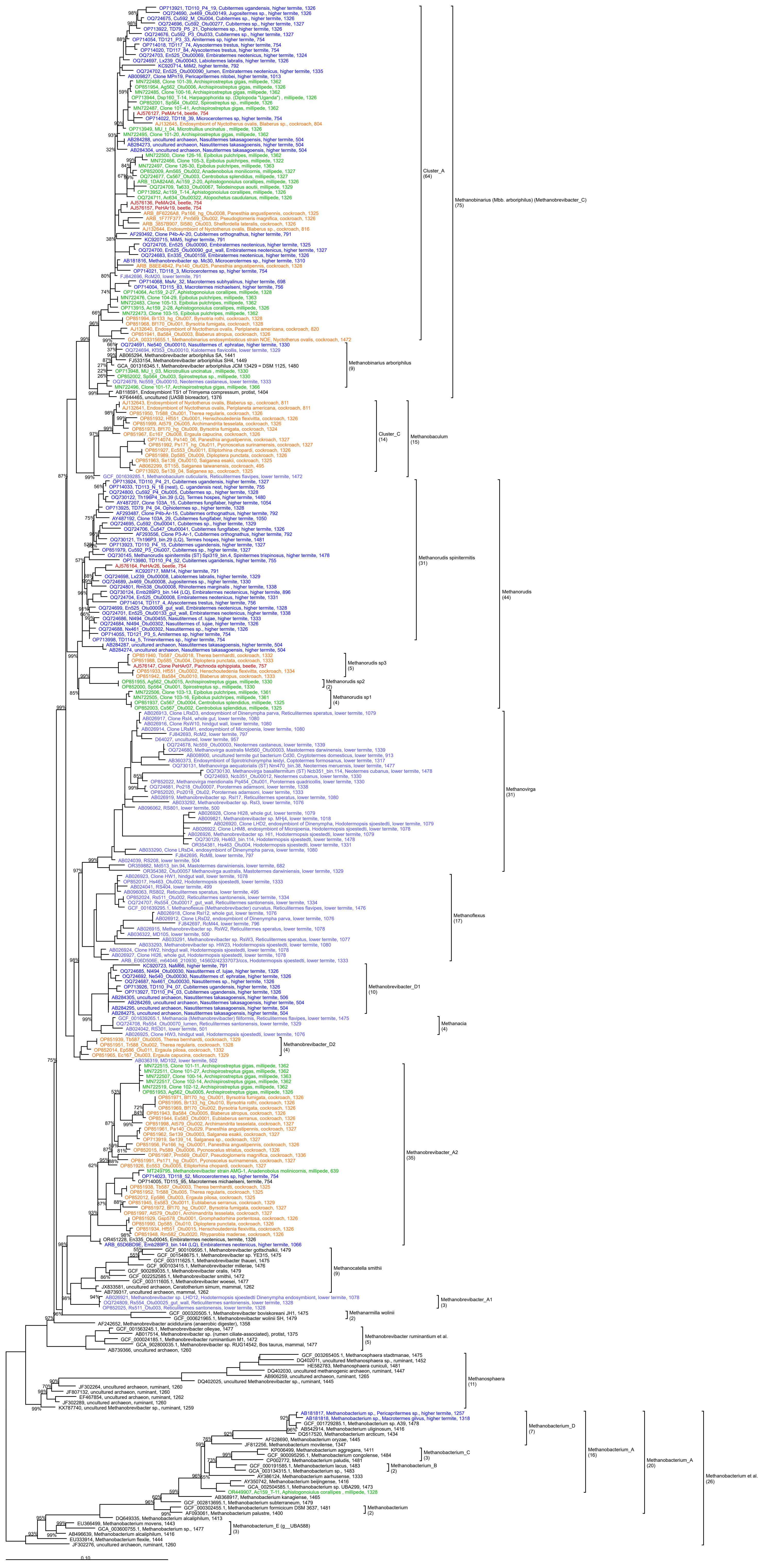
Cluster_A
(64)
Methanobinarius (Mbb. arboriphilus) (Methanobrevibacter_C)
(75)
Methanobinarius arboriphilus
(9)
Cluster_C
(14)
Methanobaculum
(15)
Methanorudis spinitermitis
(31)
Methanorudis
(44)
Methanorudis sp3
(5)
Methanorudis sp2
(2)
Methanorudis sp1
(4)
Methanovirga
(31)
Methanoflexus
(17)
Methanobrevibacter_D1
(10)
Methanacia
(4)
Methanobrevibacter_D2
(4)
Methanobrevibacter_A2
(35)
Methanocatella smithii
(9)
Methanobrevibacter_A1
(3)
Methanarmilla wolinii
(2)
Methanobrevibacter ruminantium et al.
(5)
Methanosphaera
(11)
Methanobacterium_D
(7)
Methanobacterium_A
(16)
Methanobacterium_A
(20)
Methanobacterium et al.
(26)
Methanobacterium_C
(3)
Methanobacterium_B
(2)
Methanobacterium
(2)
Methanobacterium_E (g__UBA588)
(3)
0.10

Figure S4

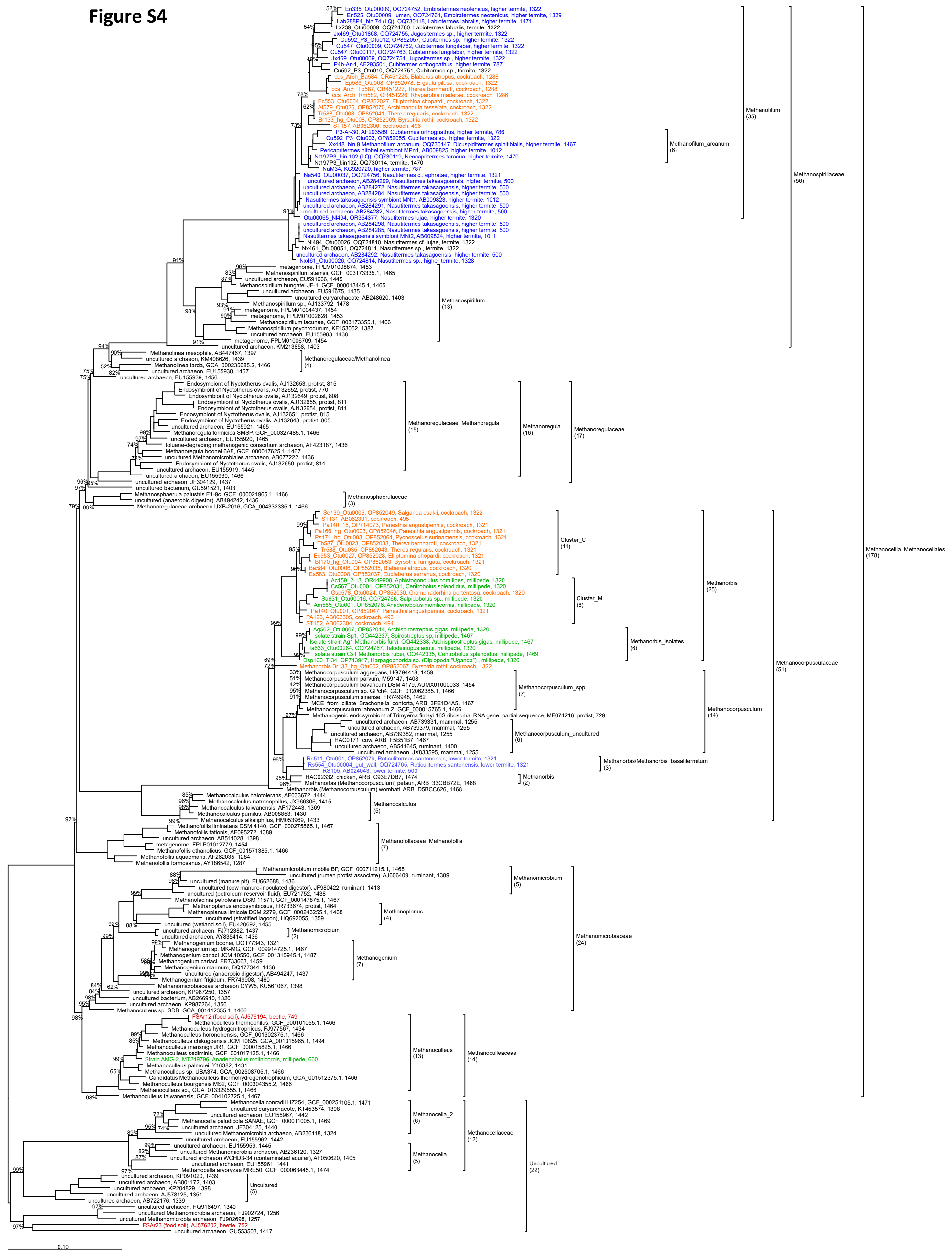

En335_Otu00009, OQ724752, Embiratermes neotenicus, higher termite, 1322
En525_Otu00009_lumen, OQ724761, Embiratermes neotenicus, higher termite, 1329
Lab288P4_bin.74 (LQ), OQ730118, Labiotermes labralis, higher termite, 1471
Lx239_Otu00009, OQ724760, Labiotermes labralis, termite, 1322
Jx469_Otu01868, OQ724755, Jugositermes sp., higher termite, 1322
Cu592_P3_Otu012, OP852057, Cubitermes sp., higher termite, 1322
Cu547_Otu00009, OQ724762, Cubitermes fungifaber, higher termite, 1322
Cu547_Otu00117, OQ724763, Cubitermes fungifaber, higher termite, 1322
Jx469_Otu00009, OQ724754, Jugositermes sp., higher termite, 1322
P4b-Ar-4, AF293501, Cubitermes orthognathus, higher termite, 787
Cu592_P3_Otu010, OQ724751, Cubitermes sp., termite, 1322
ccs_Arch_Ba584, OR451225, Blaberus atropus, cockroach, 1288
Ep586_Otu008, OP852078, Ergaula pilosa, cockroach, 1322
ccs_Arch_Tb587, OR451227, Therea bernhardti, cockroach, 1288
ccs_Arch_Rm582, OR451226, Rhyparobia maderae, cockroach, 1286
Ec553_Otu0004, OP852027, Elliptorhina chopardi, cockroach, 1322
At579_Otu025, OP852070, Archimandrita tesselata, cockroach, 1322
Tr588_Otu008, OP852041, Therea regularis, cockroach, 1322
Br133_hg_Otu008, OP852069, Byrsotria rothi, cockroach, 1322
ST157, AB062300, cockroach, 496
P3-Ar-30, AF293589, Cubitermes orthognathus, higher termite, 786
Cu592_P3_Otu003, OP852055, Cubitermes sp., higher termite, 1322
Xx448_bin.9 Methanofilum arcanum, OQ730147, Dicuspiditermes spinitibialis, higher termite, 1467
Pericapritermes nitobei symbiont MPn1, AB009825, higher termite, 1012
Nt197P3_bin.102 (LQ), OQ730119, Neocapritermes taracua, higher termite, 1470
Nt197P3_bin102, OQ730114, termite, 1470
NaM34, KC920720, higher termite, 787
Ne540_Otu00037, OQ724756, Nasutitermes cf. ephratae, higher termite, 1321
uncultured archaeon, AB284299, Nasutitermes takasagoensis, higher termite, 500
uncultured archaeon, AB284272, Nasutitermes takasagoensis, higher termite, 500
uncultured archaeon, AB284284, Nasutitermes takasagoensis, higher termite, 500
Nasutitermes takasagoensis symbiont MNT1, AB009823, higher termite, 1012
uncultured archaeon, AB284291, Nasutitermes takasagoensis, higher termite, 500
uncultured archaeon, AB284282, Nasutitermes takasagoensis, higher termite, 500
Otu00065_Nl494, OR354377, Nasutitermes lujae, higher termite, 1320
uncultured archaeon, AB284298, Nasutitermes takasagoensis, higher termite, 500
uncultured archaeon, AB284285, Nasutitermes takasagoensis, higher termite, 500
Nasutitermes takasagoensis symbiont MNT2, AB009824, higher termite, 1011
Nl494_Otu00026, OQ724810, Nasutitermes cf. lujae, termite, 1322
Nx461_Otu00051, OQ724811, Nasutitermes sp., termite, 1322
uncultured archaeon, AB284292, Nasutitermes takasagoensis, higher termite, 500
Nx461_Otu00026, OQ724814, Nasutitermes sp., higher termite, 1328
metagenome, FPLM01008874, 1453
Methanospirillum stamsii, GCF_003173335.1, 1465
uncultured archaeon, EU591666, 1445
Methanospirillum hungatei JF-1, GCF_000013445.1, 1465
uncultured archaeon, EU591675, 1435
uncultured euryarchaeote, AB248620, 1403
Methanospirillum sp., AJ133792, 1478
metagenome, FPLM01004437, 1454
metagenome, FPLM01002628, 1453
Methanospirillum lacunae, GCF_003173355.1, 1466
Methanospirillum psychrodurum, KF153052, 1387
uncultured archaeon, EU155983, 1438
metagenome, FPLM01006709, 1454
uncultured archaeon, KM213858, 1403
Methanolinea mesophila, AB447467, 1397
uncultured archaeon, KM408626, 1439
Methanolinea tarda, GCA_000235685.2, 1466
uncultured archaeon, EU155938, 1467
uncultured archaeon, EU155939, 1456
Endosymbiont of Nyctotherus ovalis, AJ132653, protist, 815
Endosymbiont of Nyctotherus ovalis, AJ132652, protist, 770
Endosymbiont of Nyctotherus ovalis, AJ132649, protist, 808
Endosymbiont of Nyctotherus ovalis, AJ132655, protist, 811
Endosymbiont of Nyctotherus ovalis, AJ132654, protist, 811
Endosymbiont of Nyctotherus ovalis, AJ132651, protist, 815
Endosymbiont of Nyctotherus ovalis, AJ132648, protist, 805
uncultured archaeon, EU155921, 1465
Methanoregula formicica SMSP, GCF_000327485.1, 1466
uncultured archaeon, EU155920, 1465
toluene-degrading methanogenic consortium archaeon, AF423187, 1436
Methanoregula boonei 6A8, GCF_000017625.1, 1467
uncultured Methanomicrobiales archaeon, AB077222, 1436
Endosymbiont of Nyctotherus ovalis, AJ132650, protist, 814
uncultured archaeon, EU155919, 1445
uncultured archaeon, EU155930, 1466
uncultured archaeon, JF304129, 1437
uncultured bacterium, GU591521, 1403
Methanosphaerula palustris E1-9c, GCF_000021965.1, 1466
uncultured (anaerobic digestor), AB494242, 1436
Methanoregulaceae archaeon UXB-2016, GCA_004332335.1, 1466
Se139_Otu0006, OP852049, Salganea esakii, cockroach, 1322
ST131, AB062301, cockroach, 495
Pa140_15, OP714073, Panesthia angustipennis, cockroach, 1321
Pa166_hg_Otu0003, OP852046, Panesthia angustipennis, cockroach, 1321
Ps171_hg_Otu003, OP852064, Pycnoscelus surinamensis, cockroach, 1321
Tb587_Otu0023, OP852033, Therea bernhardti, cockroach, 1321
Tr588_Otu035, OP852043, Therea regularis, cockroach, 1321
Ec553_Otu0027, OP852028, Elliptorhina chopardi, cockroach, 1321
Bf170_hg_Otu004, OP852053, Byrsotria fumigata, cockroach, 1321
Ba584_Otu0006, OP852035, Blaberus atropus, cockroach, 1320
Es583_Otu0008, OP852037, Eublaberus serranus, cockroach, 1320
Ac159_2-13, OR449908, Aphistogoniulus corallipes, millipede, 1320
Cs567_Otu0001, OP852031, Centrobolus splendidus, millipede, 1320
Gsp578_Otu0024, OP852030, Gromphadorhina portentosa, cockroach, 1320
Sa631_Otu00016, OQ724766, Salpidobolus sp., millipede, 1320
Am565_Otu001, OP852076, Anadenobolus monilicornis, millipede, 1320
Pa140_Otu001, OP852047, Panesthia angustipennis, cockroach, 1321
PA123, AB062305, cockroach, 493
ST152, AB062304, cockroach, 494
Ag562_Otu0007, OP852044, Archispirostreptus gigas, millipede, 1320
Isolate strain Sp1, OQ442337, Spirostreptus sp, millipede, 1467
Isolate strain Ag1 Methanorbis furvi, OQ442338, Archispirostreptus gigas, millipede, 1467
Ta633_Otu00264, OQ724767, Telodeinopus aoutii, millipede, 1320
Isolate strain Cs1 Methanorbis rubei, OQ442335, Centrobolus splendidus, millipede, 1469
Dsp160_T-34, OP713947, Harpagophorida sp. (Diplopoda "Uganda") , millipede, 1320
Methanorbis Br133_hg_Otu002, OP852067, Byrsotria rothi, cockroach, 1322
Methanocorpusculum aggregans, HG794418, 1459
Methanocorpusculum parvum, M59147, 1408
Methanocorpusculum bavaricum DSM 4179, AUXM01000033, 1454
Methanocorpusculum sp. GPch4, GCF_012062385.1, 1466
Methanocorpusculum sinense, FR749948, 1462
MCE_from_ciliate_Brachonella_contorta, ARB_3FE1D4A5, 1467
Methanocorpusculum labreanum Z, GCF_000015765.1, 1466
Methanogenic endosymbiont of Trimyema finlayi 16S ribosomal RNA gene, partial sequence, MF074216, protist, 729
uncultured archaeon, AB739331, mammal, 1255
uncultured archaeon, AB739379, mammal, 1255
uncultured archaeon, AB739382, mammal, 1255
HAC0171_cow, ARB_F5B51B7, 1467
uncultured archaeon, AB541645, ruminant, 1400
uncultured archaeon, JX833595, mammal, 1255
Rs511_Otu001, OP852079, Reticulitermes santonensis, lower termite, 1321
Rs554_Otu00004_gut_wall, OQ724765, Reticulitermes santonensis, lower termite, 1321
RS105, AB024043, lower termite, 500
HAC02332_chicken, ARB_C93E7DB7, 1474
Methanorbis (Methanocorpusculum) petauri, ARB_33CBB72E, 1468
Methanorbis (Methanocorpusculum) wombati, ARB_D5BCC626, 1468
Methanocalculus halotolerans, AF033672, 1444
Methanocalculus natronophilus, JX966306, 1415
Methanocalculus taiwanensis, AF172443, 1369
Methanocalculus pumilus, AB008853, 1430
Methanocalculus alkaliphilus, HM053969, 1433
Methanofollis liminatans DSM 4140, GCF_000275865.1, 1467
Methanofollis tationis, AF095272, 1389
uncultured archaeon, AB511028, 1398
metagenome, FPLP01012779, 1454
Methanofollis ethanolicus, GCF_001571385.1, 1466
Methanofollis aquaemaris, AF262035, 1284
Methanofollis formosanus, AY186542, 1287
Methanomicrobium mobile BP, GCF_000711215.1, 1468
uncultured (rumen protist associate), AJ606409, ruminant, 1309
uncultured (manure pit), EU662688, 1436
uncultured (cow manure-inoculated digestor), JF980422, ruminant, 1413
uncultured (petroleum reservoir fluid), EU721752, 1438
Methanolacinia petrolearia DSM 11571, GCF_000147875.1, 1467
Methanoplanus endosymbiosus, FR733674, protist, 1464
Methanoplanus limicola DSM 2279, GCF_000243255.1, 1468
uncultured (stratified lagoon), HQ692055, 1359
uncultured (wetland soil), EU420692, 1455
uncultured archaeon, FJ712382, 1437
uncultured archaeon, AY835414, 1436
Methanogenium boonei, DQ177343, 1321
Methanogenium sp. MK-MG, GCF_009914725.1, 1467
Methanoculleus cariaci JCM 10550, GCF_001315945.1, 1487
Methanogenium cariaci, FR733663, 1459
Methanogenium marinum, DQ177344, 1436
uncultured (anaerobic digestor), AB494247, 1437
Methanogenium frigidum, FR749908, 1460
Methanomicrobiaceae archaeon CYW5, KU561067, 1398
uncultured archaeon, KP987250, 1357
uncultured bacterium, AB266910, 1320
uncultured archaeon, KP987264, 1356
Methanoculleus sp. SDB, GCA_001412355.1, 1466
FSAr12 (food soil), AJ576194, beetle, 749
Methanoculleus thermophilus, GCF_900101055.1, 1466
Methanoculleus hydrogenitrophicus, FJ977567, 1434
Methanoculleus horonobensis, GCF_001602375.1, 1466
Methanoculleus chikugoensis JCM 10825, GCA_001315965.1, 1494
Methanoculleus marisnigri JR1, GCF_000015825.1, 1466
Methanoculleus sediminis, GCF_001017125.1, 1466
Strain AMG-2, MT249796, Anadenobolus molinicornis, millipede, 660
Methanoculleus palmolei, Y16382, 1431
Methanoculleus sp. UBA374, GCA_002508705.1, 1466
Candidatus Methanoculleus thermohydrogenotrophicum, GCA_001512375.1, 1466
Methanoculleus bourgensis MS2, GCF_000304355.2, 1466
Methanoculleus sp., GCA_013329555.1, 1466
Methanoculleus taiwanensis, GCF_004102725.1, 1467
Methanocella conradii HZ254, GCF_000251105.1, 1471
uncultured euryarchaeote, KT453574, 1308
uncultured archaeon, EU155967, 1442
Methanocella paludicola SANAE, GCF_000011005.1, 1469
uncultured archaeon, JF304125, 1440
uncultured Methanomicrobia archaeon, AB236118, 1324
uncultured archaeon, EU155962, 1442
uncultured archaeon, EU155959, 1445
uncultured Methanomicrobia archaeon, AB236120, 1327
uncultured archaeon WCHD3-34 (contaminated aquifer), AF050620, 1405
uncultured archaeon, EU155961, 1441
Methanocella arvoryzae MRE50, GCF_000063445.1, 1474
uncultured archaeon, KP091020, 1439
uncultured archaeon, AB801172, 1403
uncultured archaeon, KP204829, 1398
uncultured archaeon, AJ578125, 1351
uncultured archaeon, AB722176, 1339
uncultured archaeon, HQ916497, 1340
uncultured Methanomicrobia archaeon, FJ902724, 1256
uncultured Methanomicrobia archaeon, FJ902698, 1257
FSAr23 (food soil), AJ576202, beetle, 752
uncultured archaeon, GU553503, 1417
Methanofilum_arcanum (6)
Methanofilum (35)
Methanospirillaceae (56)
Methanospirillum (13)
Methanoregulaceae/Methanolinea (4)
Methanoregulaceae_Methanoregula (15)
Methanoregula (16)
Methanoregulaceae (17)
Methanosphaerulaceae (3)
Cluster_C (11)
Cluster_M (8)
Methanorbis_isolates (6)
Methanorbis (25)
Methanocorpusculum_spp (7)
Methanocorpusculum_uncultured (6)
Methanocorpusculum (14)
Methanocorpusculaceae (51)
Methanorbis/Methanorbis_basalitermitum (3)
Methanorbis (2)
Methanocalculus (5)
Methanofollaceae_Methanofollis (7)
Methanomicrobium (5)
Methanoplanus (4)
Methanomicrobium (2)
Methanogenium (7)
Methanomicrobiaceae (24)
Methanoculleus (13)
Methanocullaceae (14)
Methanocella_2 (6)
Methanocella (5)
Methanocellaceae (12)
Uncultured (5)
Uncultured (22)
Methanocellia_Methanocellales (178)
0.10

Figure S5

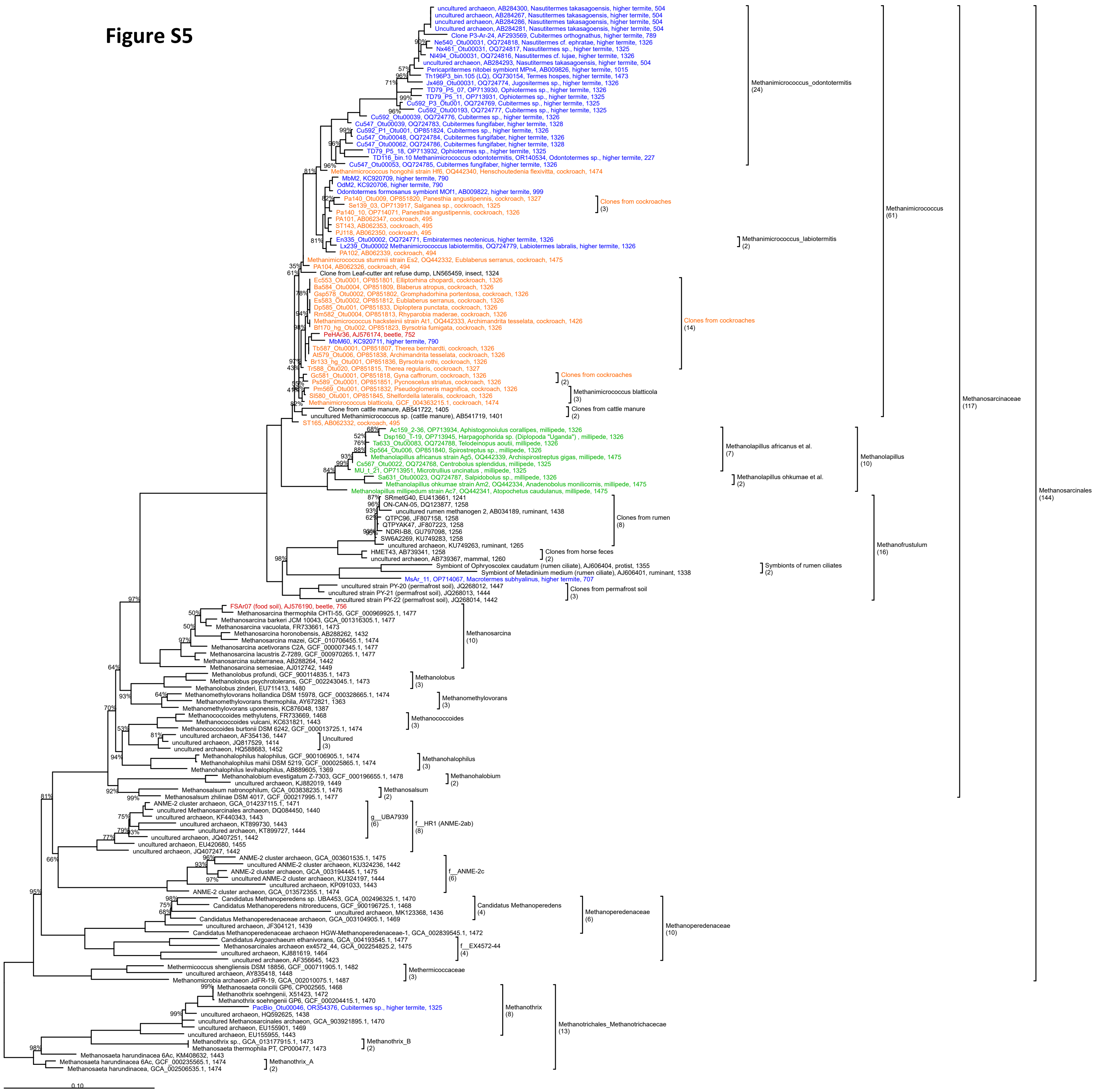

uncultured archaeon, AB284300, Nasutitermes takasagoensis, higher termite, 504
uncultured archaeon, AB284267, Nasutitermes takasagoensis, higher termite, 504
uncultured archaeon, AB284286, Nasutitermes takasagoensis, higher termite, 504
Uncultured archaeon, AB284281, Nasutitermes takasagoensis, higher termite, 504
Clone P3-Ar-24, AF293569, Cubitermes orthognathus, higher termite, 789
Ne540_Otu00031, OQ724818, Nasutitermes cf. ephratae, higher termite, 1326
Nx461_Otu00031, OQ724817, Nasutitermes sp., higher termite, 1325
Nl494_Otu00031, OQ724816, Nasutitermes cf. lujae, higher termite, 1326
uncultured archaeon, AB284293, Nasutitermes takasagoensis, higher termite, 504
Pericapritermes nitobei symbiont MPn4, AB009826, higher termite, 1015
Th196P3_bin.105 (LQ), OQ730154, Termes hospes, higher termite, 1473
Jx469_Otu00031, OQ724774, Jugositermes sp., higher termite, 1326
TD79_P5_07, OP713930, Ophiotermes sp., higher termite, 1326
TD79_P5_11, OP713931, Ophiotermes sp., higher termite, 1325
Cu592_P3_Otu001, OQ724769, Cubitermes sp., higher termite, 1325
Cu592_Otu00193, OQ724777, Cubitermes sp., higher termite, 1325
Cu592_Otu00039, OQ724776, Cubitermes sp., higher termite, 1326
Cu547_Otu00039, OQ724783, Cubitermes fungifaber, higher termite, 1328
Cu592_P1_Otu001, OP851824, Cubitermes sp., higher termite, 1326
Cu547_Otu00048, OQ724784, Cubitermes fungifaber, higher termite, 1326
Cu547_Otu00062, OQ724786, Cubitermes fungifaber, higher termite, 1328
TD79_P5_18, OP713932, Ophiotermes sp., higher termite, 1325
TD116_bin.10 Methanimicrococcus odontotermitis, OR140534, Odontotermes sp., higher termite, 227
Cu547_Otu00053, OQ724785, Cubitermes fungifaber, higher termite, 1326
Methanimicrococcus hongohii strain Hf6, OQ442340, Henschoutedenia flexivitta, cockroach, 1474
MbM2, KC920709, higher termite, 790
OdM2, KC920706, higher termite, 790
Odontotermes formosanus symbiont MOf1, AB009822, higher termite, 999
Pa140_Otu009, OP851820, Panesthia angustipennis, cockroach, 1327
Se139_03, OP713917, Salganea sp., cockroach, 1325
Pa140_10, OP714071, Panesthia angustipennis, cockroach, 1326
PA101, AB062347, cockroach, 495
ST143, AB062353, cockroach, 495
PJ118, AB062350, cockroach, 495
En335_Otu00002, OQ724771, Embiratermes neotenicus, higher termite, 1326
Lx239_Otu00002 Methanimicrococcus labiotermitis, OQ724779, Labiotermes labralis, higher termite, 1326
PA102, AB062339, cockroach, 494
Methanimicrococcus stummii strain Es2, OQ442332, Eublaberus serranus, cockroach, 1475
PA104, AB062326, cockroach, 494
Clone from Leaf-cutter ant refuse dump, LN565459, insect, 1324
Ec553_Otu0001, OP851801, Elliptorhina chopardi, cockroach, 1326
Ba584_Otu0004, OP851809, Blaberus atropus, cockroach, 1326
Gsp578_Otu0002, OP851802, Gromphadorhina portentosa, cockroach, 1326
Es583_Otu0002, OP851812, Eublaberus serranus, cockroach, 1326
Dp585_Otu001, OP851833, Diploptera punctata, cockroach, 1326
Rm582_Otu0004, OP851813, Rhyparobia maderae, cockroach, 1326
Methanimicrococcus hacksteinii strain At1, OQ442333, Archimandrita tesselata, cockroach, 1426
Bf170_hg_Otu002, OP851823, Byrsotria fumigata, cockroach, 1326
PeHAr36, AJ576174, beetle, 752
MbM60, KC920711, higher termite, 790
Tb587_Otu0001, OP851807, Therea bernhardti, cockroach, 1326
At579_Otu006, OP851838, Archimandrita tesselata, cockroach, 1326
Br133_hg_Otu001, OP851836, Byrsotria rothi, cockroach, 1326
Tr588_Otu020, OP851815, Therea regularis, cockroach, 1327
Gc581_Otu0001, OP851818, Gyna caffrorum, cockroach, 1326
Ps589_Otu0001, OP851851, Pycnoscelus striatus, cockroach, 1326
Pm569_Otu001, OP851832, Pseudoglomeris magnifica, cockroach, 1326
Sl580_Otu001, OP851845, Shelfordella lateralis, cockroach, 1326
Methanimicrococcus blatticola, GCF_004363215.1, cockroach, 1474
Clone from cattle manure, AB541722, 1405
uncultured Methanimicrococcus sp. (cattle manure), AB541719, 1401
ST165, AB062332, cockroach, 495
Ac159_2-36, OP713934, Aphistogoniulus corallipes, millipede, 1326
Dsp160_T-19, OP713945, Harpagophorida sp. (Diplopoda "Uganda") , millipede, 1326
Ta633_Otu00083, OQ724788, Telodeinopus aoutii, millipede, 1326
Sp564_Otu006, OP851840, Spirostreptus sp., millipede, 1326
Methanolapillus africanus strain Ag5, OQ442339, Archispirostreptus gigas, millipede, 1475
Cs567_Otu0022, OQ724768, Centrobolus splendidus, millipede, 1325
MU_t_21, OP713951, Microtrullius uncinatus , millipede, 1325
Sa631_Otu00023, OQ724787, Salpidobolus sp., millipede, 1326
Methanolapillus ohkumae strain Am2, OQ442334, Anadenobolus monilicornis, millipede, 1475
Methanolapillus millepedum strain Ac7, OQ442341, Atopochetus caudulanus, millipede, 1475
SRmetG40, EU413661, 1241
ON-CAN-05, DQ123877, 1258
uncultured rumen methanogen 2, AB034189, ruminant, 1438
QTPC96, JF807158, 1258
QTPYAK47, JF807223, 1258
NDRI-B8, GU797098, 1256
SW6A2269, KU749283, 1258
uncultured archaeon, KU749263, ruminant, 1265
HMET43, AB739341, 1258
uncultured archaeon, AB739367, mammal, 1260
Symbiont of Ophryoscolex caudatum (rumen ciliate), AJ606404, protist, 1355
Symbiont of Metadinium medium (rumen ciliate), AJ606401, ruminant, 1338
MsAr_11, OP714067, Macrotermes subhyalinus, higher termite, 707
uncultured strain PY-20 (permafrost soil), JQ268012, 1447
uncultured strain PY-21 (permafrost soil), JQ268013, 1444
uncultured strain PY-22 (permafrost soil), JQ268014, 1442
FSAr07 (food soil), AJ576190, beetle, 756
Methanosarcina thermophila CHTI-55, GCF_000969925.1, 1477
Methanosarcina barkeri JCM 10043, GCA_001316305.1, 1477
Methanosarcina vacuolata, FR733661, 1473
Methanosarcina horonobensis, AB288262, 1432
Methanosarcina mazei, GCF_010706455.1, 1474
Methanosarcina acetivorans C2A, GCF_000007345.1, 1477
Methanosarcina lacustris Z-7289, GCF_000970265.1, 1477
Methanosarcina subterranea, AB288264, 1442
Methanosarcina semesiae, AJ012742, 1449
Methanolobus profundi, GCF_900114835.1, 1473
Methanolobus psychrotolerans, GCF_002243045.1, 1473
Methanolobus zinderi, EU711413, 1480
Methanomethylovorans hollandica DSM 15978, GCF_000328665.1, 1474
Methanomethylovorans thermophila, AY672821, 1363
Methanomethylovorans uponensis, KC876048, 1387
Methanococcoides methylutens, FR733669, 1468
Methanococcoides vulcani, KC631821, 1443
Methanococcoides burtonii DSM 6242, GCF_000013725.1, 1474
uncultured archaeon, AF354136, 1447
uncultured archaeon, JQ817529, 1414
uncultured archaeon, HQ588683, 1452
Methanohalophilus halophilus, GCF_900106905.1, 1474
Methanohalophilus mahii DSM 5219, GCF_000025865.1, 1474
Methanohalophilus levihalophilus, AB889605, 1369
Methanohalobium evestigatum Z-7303, GCF_000196655.1, 1478
uncultured archaeon, KJ882019, 1449
Methanosalsum natronophilum, GCA_003838235.1, 1476
Methanosalsum zhilinae DSM 4017, GCF_000217995.1, 1477
ANME-2 cluster archaeon, GCA_014237115.1, 1471
uncultured Methanosarcinales archaeon, DQ084450, 1440
uncultured archaeon, KF440343, 1443
uncultured archaeon, KT899730, 1443
uncultured archaeon, KT899727, 1444
uncultured archaeon, JQ407251, 1442
uncultured archaeon, EU420680, 1455
uncultured archaeon, JQ407247, 1442
ANME-2 cluster archaeon, GCA_003601535.1, 1475
uncultured ANME-2 cluster archaeon, KU324236, 1442
ANME-2 cluster archaeon, GCA_003194445.1, 1475
uncultured ANME-2 cluster archaeon, KU324197, 1444
uncultured archaeon, KP091033, 1443
ANME-2 cluster archaeon, GCA_013572355.1, 1474
Candidatus Methanoperedens sp. UBA453, GCA_002496325.1, 1470
Candidatus Methanoperedens nitroreducens, GCF_900196725.1, 1468
uncultured archaeon, MK123368, 1436
Candidatus Methanoperedenaceae archaeon, GCA_003104905.1, 1469
uncultured archaeon, JF304121, 1439
Candidatus Methanoperedenaceae archaeon HGW-Methanoperedenaceae-1, GCA_002839545.1, 1472
Candidatus Argoarchaeum ethanivorans, GCA_004193545.1, 1477
Methanosarcinales archaeon ex4572_44, GCA_002254825.2, 1475
uncultured archaeon, KJ881619, 1464
uncultured archaeon, AF356645, 1423
Methermicoccus shengliensis DSM 18856, GCF_000711905.1, 1482
uncultured archaeon, AY835418, 1448
Methanomicrobia archaeon JdFR-19, GCA_002010075.1, 1487
Methanosaeta concilii GP6, CP002565, 1468
Methanothrix soehngenii, X51423, 1472
Methanothrix soehngenii GP6, GCF_000204415.1, 1470
PacBio_Otu00046, OR354376, Cubitermes sp., higher termite, 1325
uncultured archaeon, HQ592625, 1438
uncultured Methanosarcinales archaeon, GCA_903921895.1, 1470
uncultured archaeon, EU155901, 1469
uncultured archaeon, EU155955, 1443
Methanothrix sp., GCA_013177915.1, 1473
Methanosaeta thermophila PT, CP000477, 1473
Methanosaeta harundinacea 6Ac, KM408632, 1443
Methanosaeta harundinacea 6Ac, GCF_000235565.1, 1474
Methanosaeta harundinacea, GCA_002506535.1, 1474
0.10
Methanimicrococcus_odontotermitis (24)
Clones from cockroaches (3)
Methanimicrococcus_labiotermitis (2)
Clones from cockroaches (14)
Clones from cockroaches (2)
Methanimicrococcus blatticola (3)
Clones from cattle manure (2)
Methanimicrococcus (61)
Methanolapillus africanus et al. (7)
Methanolapillus ohkumae et al. (2)
Methanolapillus (10)
Clones from rumen (8)
Clones from horse feces (2)
Symbionts of rumen ciliates (2)
Clones from permafrost soil (3)
Methanofrustulum (16)
Methanosarcinaceae (117)
Methanosarcinales (144)
Methanosarcina (10)
Methanolobus (3)
Methanomethylovorans (3)
Methanococcoides (3)
Uncultured (3)
Methanohalophilus (3)
Methanohalobium (2)
Methanosalsum (2)
g__UBA7939 (6)
f__HR1 (ANME-2ab) (8)
f__ANME-2c (6)
Candidatus Methanoperedens (4)
Methanoperedenaceae (6)
Methanoperedenaceae (10)
f__EX4572-44 (4)
Methermicoccaceae (3)
Methanothrix (8)
Methanothrix_B (2)
Methanothrix_A (2)
Methanotrichales_Methanotrichaceae (13)

Figure S6

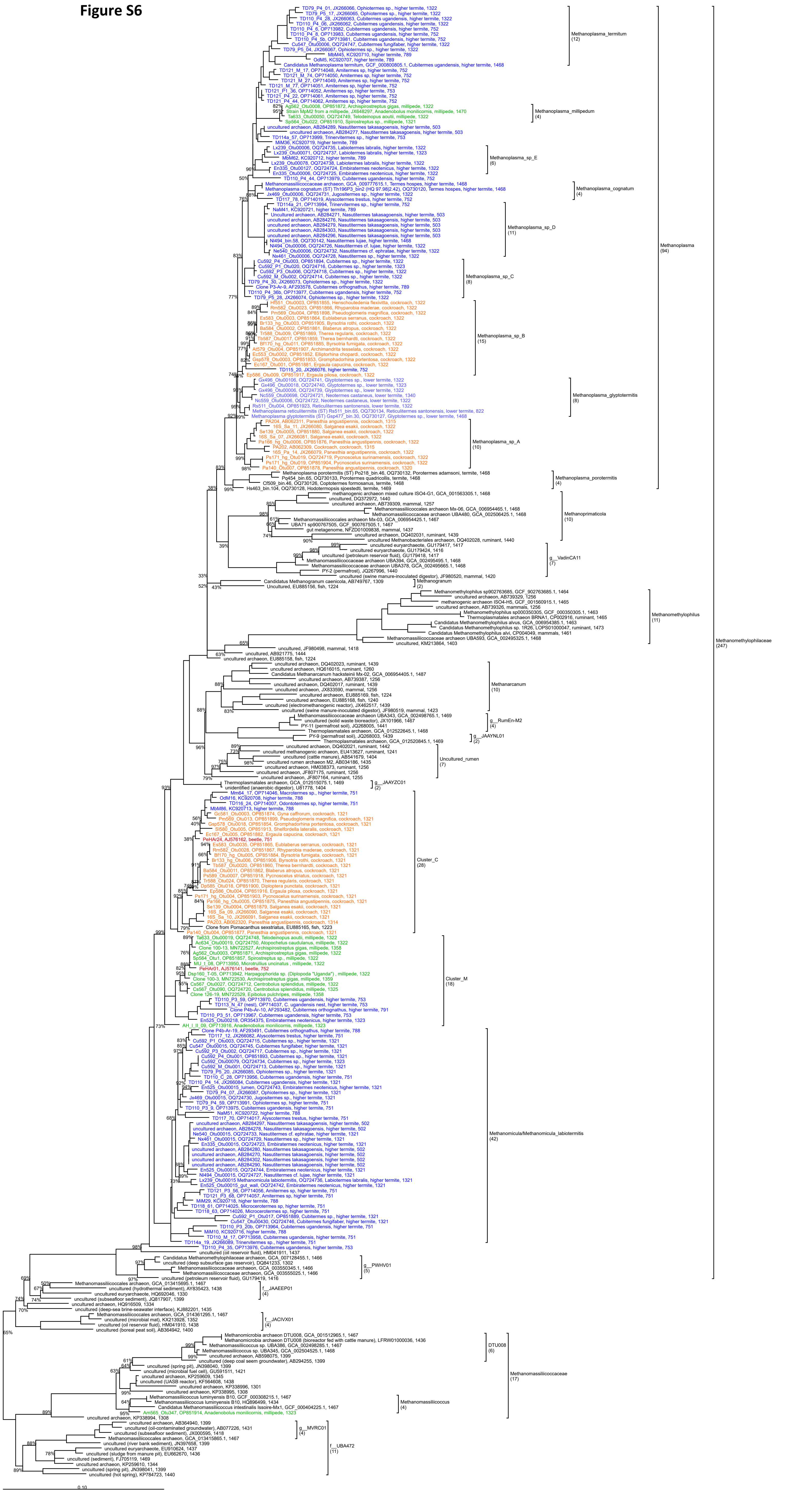

Methanoplasma_termitum
(12)
Methanoplasma_millipedum
(4)
Methanoplasma_sp_E
(6)
Methanoplasma_cognatum
(4)
Methanoplasma_sp_D
(11)
Methanoplasma_sp_C
(8)
Methanoplasma_sp_B
(15)
Methanoplasma_glyptotermitis
(8)
Methanoplasma_sp_A
(10)
Methanoplasma_poretermitis
(4)
Methanoplasma
(94)
Methanoprimaticola
(10)
g__VadinCA11
(7)
Methanogranum
(2)
Methanomethylophilus
(11)
Methanomethylophilaceae
(247)
Methanarcanum
(10)
g__RumEn-M2
(4)
g__JAAYNL01
(2)
Uncultured_rumen
(7)
g__JAAYZC01
(2)
Cluster_C
(28)
Cluster_M
(18)
Methanomicula/Methanomicula_labiotermitis
(42)
g__PWHV01
(5)
f__JAAEEP01
(4)
f__JACIVX01
(4)
DTU008
(6)
Methanomassiliicoccaceae
(17)
Methanomassiliicoccus
(4)
g__MVRC01
(4)
f__UBA472
(11)
0.10

Figure S7

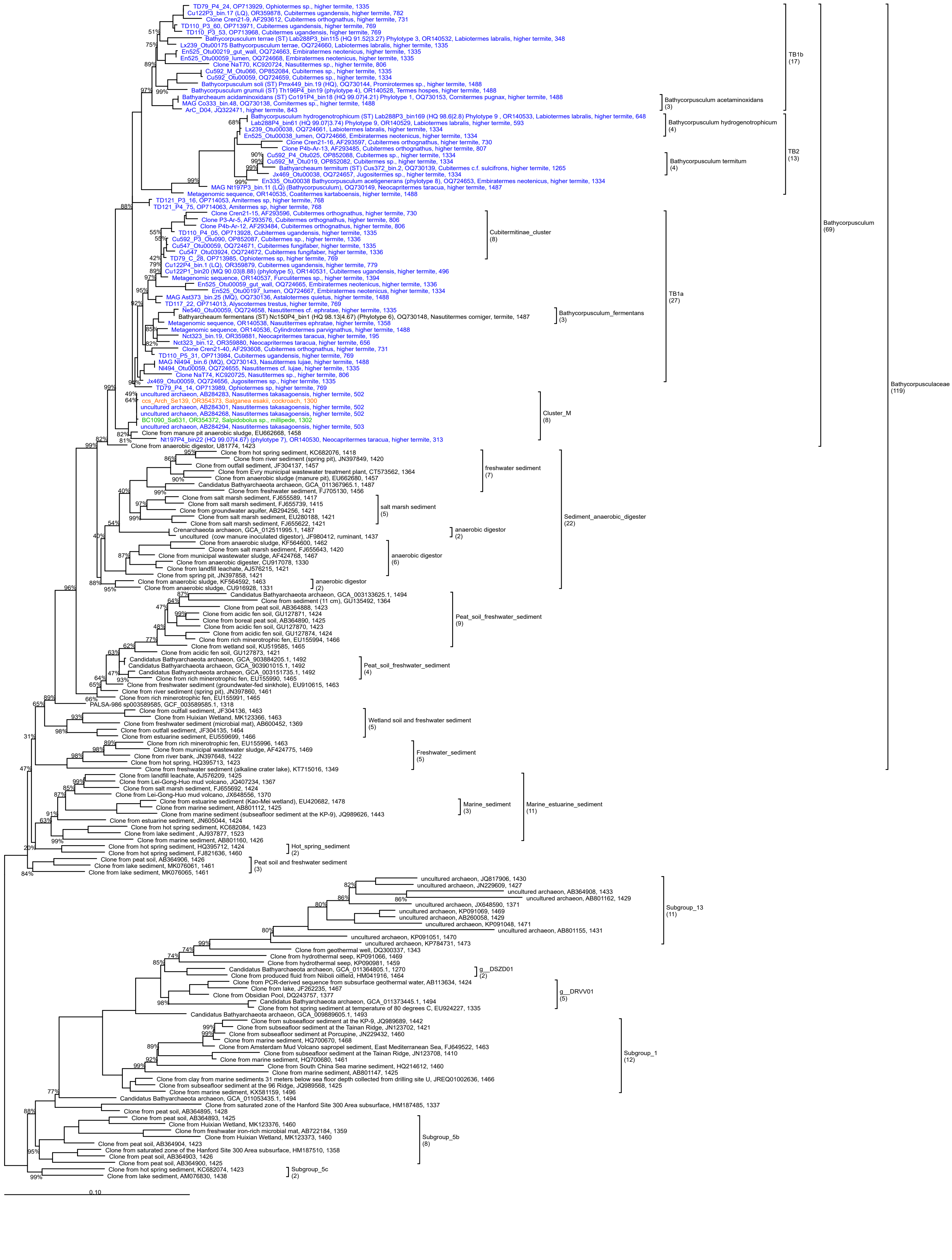

TD79_P4_24, OP713929, Ophiotermes sp., higher termite, 1335
Cu122P3_bin.17 (LQ), OR359878, Cubitermes ugandensis, higher termite, 782
Clone Cren21-9, AF293612, Cubitermes orthognathus, higher termite, 731
TD110_P3_60, OP713971, Cubitermes ugandensis, higher termite, 769
TD110_P3_53, OP713968, Cubitermes ugandensis, higher termite, 769
Bathycorpusculum terrae (ST) Lab288P3_bin115 (HQ 91.52|3.27) Phylotype 3, OR140532, Labiotermes labralis, higher termite, 348
Lx239_Otu00175 Bathycorpusculum terrae, OQ724660, Labiotermes labralis, higher termite, 1335
En525_Otu00219_gut_wall, OQ724663, Embiratermes neotenicus, higher termite, 1335
En525_Otu00059_lumen, OQ724668, Embiratermes neotenicus, higher termite, 1335
Clone NaT70, KC920724, Nasutitermes sp., higher termite, 806
Cu592_M_Otu066, OP852084, Cubitermes sp., higher termite, 1335
Cu592_Otu00059, OQ724659, Cubitermes sp., higher termite, 1334
Bathycorpusculum soli (ST) Pmx449_bin.19 (HQ), OQ730144, Promirotermes sp., higher termite, 1488
Bathycorpusculum grumuli (ST) Th196P4_bin19 (phylotype 4), OR140528, Termes hospes, higher termite, 1488
Bathyarcheaum acidaminoxidans (ST) Co191P4_bin18 (HQ 99.07|4.21) Phylotype 1, OQ730153, Cornitermes pugnax, higher termite, 1488
MAG Co333_bin.48, OQ730138, Cornitermes sp., higher termite, 1488
ArC_D04, JQ322471, higher termite, 843
Bathycorpusculum hydrogenotrophicum (ST) Lab288P3_bin169 (HQ 98.6|2.8) Phylotype 9 , OR140533, Labiotermes labralis, higher termite, 648
Lab288P4_bin61 (HQ 99.07|3.74) Phylotype 9, OR140529, Labiotermes labralis, higher termite, 593
Lx239_Otu00038, OQ724661, Labiotermes labralis, higher termite, 1334
En525_Otu00038_lumen, OQ724666, Embiratermes neotenicus, higher termite, 1334
Clone Cren21-16, AF293597, Cubitermes orthognathus, higher termite, 730
Clone P4b-Ar-13, AF293485, Cubitermes orthognathus, higher termite, 807
Cu592_P4_Otu025, OP852088, Cubitermes sp., higher termite, 1334
Cu592_M_Otu019, OP852082, Cubitermes sp., higher termite, 1334
Bathyarcheaum termitum (ST) Cus372_bin.2, OQ730139, Cubitermes c.f. sulcifrons, higher termite, 1265
Jx469_Otu00038, OQ724657, Jugositermes sp., higher termite, 1334
En335_Otu00038 Bathycorpusculum acetigenerans (phylotype 8), OQ724653, Embiratermes neotenicus, higher termite, 1334
MAG Nt197P3_bin.11 (LQ) (Bathycorpusculum), OQ730149, Neocapritermes taracua, higher termite, 1487
Metagenomic sequence, OR140535, Coatitermes kartaboensis, higher termite, 1488
TD121_P3_16, OP714053, Amitermes sp, higher termite, 768
TD121_P4_75, OP714063, Amitermes sp, higher termite, 768
Clone Cren21-15, AF293596, Cubitermes orthognathus, higher termite, 730
Clone P3-Ar-5, AF293576, Cubitermes orthognathus, higher termite, 806
Clone P4b-Ar-12, AF293484, Cubitermes orthognathus, higher termite, 806
TD110_P4_05, OP713928, Cubitermes ugandensis, higher termite, 1335
Cu592_P3_Otu090, OP852087, Cubitermes sp., higher termite, 1336
Cu547_Otu00059, OQ724671, Cubitermes fungifaber, higher termite, 1335
Cu547_Otu03924, OQ724672, Cubitermes fungifaber, higher termite, 1336
TD79_C_28, OP713985, Ophiotermes sp, higher termite, 769
Cu122P4_bin.1 (LQ), OR359879, Cubitermes ugandensis, higher termite, 779
Cu122P1_bin20 (MQ 90.03|8.88) (phylotype 5), OR140531, Cubitermes ugandensis, higher termite, 496
Metagenomic sequence, OR140537, Furculitermes sp., higher termite, 1394
En525_Otu00059_gut_wall, OQ724665, Embiratermes neotenicus, higher termite, 1336
En525_Otu00197_lumen, OQ724667, Embiratermes neotenicus, higher termite, 1334
MAG Ast373_bin.25 (MQ), OQ730136, Astalotermes quietus, higher termite, 1488
TD117_22, OP714013, Alyscotermes trestus, higher termite, 769
Ne540_Otu00059, OQ724658, Nasutitermes cf. ephratae, higher termite, 1335
Bathyarcheaum fermentans (ST) Nc150P4_bin1 (HQ 98.13|4.67) (Phylotype 6), OQ730148, Nasutitermes corniger, termite, 1487
Metagenomic sequence, OR140538, Nasutitermes ephratae, higher termite, 1358
Metagenomic sequence, OR140536, Cylindrotermes parvignathus, higher termite, 1488
Nct323_bin.19, OR359881, Neocapritermes taracua, higher termite, 195
Nct323_bin.12, OR359880, Neocapritermes taracua, higher termite, 656
Clone Cren21-40, AF293608, Cubitermes orthognathus, higher termite, 731
TD110_P5_31, OP713984, Cubitermes ugandensis, higher termite, 769
MAG Nl494_bin.6 (MQ), OQ730143, Nasutitermes lujae, higher termite, 1488
Nl494_Otu00059, OQ724655, Nasutitermes cf. lujae, higher termite, 1335
Clone NaT74, KC920725, Nasutitermes sp., higher termite, 806
Jx469_Otu00059, OQ724656, Jugositermes sp., higher termite, 1335
TD79_P4_14, OP713989, Ophiotermes sp, higher termite, 769
uncultured archaeon, AB284283, Nasutitermes takasagoensis, higher termite, 502
ccs_Arch_Se139, OR354373, Salganea esakii, cockroach, 1300
uncultured archaeon, AB284301, Nasutitermes takasagoensis, higher termite, 502
uncultured archaeon, AB284268, Nasutitermes takasagoensis, higher termite, 502
BC1090_Sa631, OR354372, Salpidobolus sp., millipede, 1302
uncultured archaeon, AB284294, Nasutitermes takasagoensis, higher termite, 503
Clone from manure pit anaerobic sludge, EU662668, 1458
Nt197P4_bin22 (HQ 99.07|4.67) (phylotype 7), OR140530, Neocapritermes taracua, higher termite, 313
Clone from anaerobic digestor, U81774, 1423
Clone from hot spring sediment, KC682076, 1418
Clone from river sediment (spring pit), JN397849, 1420
Clone from outfall sediment, JF304137, 1457
Clone from Evry municipal wastewater treatment plant, CT573562, 1364
Clone from anaerobic sludge (manure pit), EU662680, 1457
Candidatus Bathyarchaeota archaeon, GCA_011367965.1, 1487
Clone from freshwater sediment, FJ705130, 1456
Clone from salt marsh sediment, FJ655589, 1417
Clone from salt marsh sediment, FJ655739, 1415
Clone from groundwater aquifer, AB294256, 1421
Clone from salt marsh sediment, EU280188, 1421
Clone from salt marsh sediment, FJ655622, 1421
Crenarchaeota archaeon, GCA_012511995.1, 1487
uncultured (cow manure inoculated digestor), JF980412, ruminant, 1437
Clone from anaerobic sludge, KF564600, 1462
Clone from salt marsh sediment, FJ655643, 1420
Clone from municipal wastewater sludge, AF424768, 1467
Clone from anaerobic digester, CU917078, 1330
Clone from landfill leachate, AJ576215, 1421
Clone from spring pit, JN397858, 1421
Clone from anaerobic sludge, KF564592, 1463
Clone from anaerobic sludge, CU916928, 1331
Candidatus Bathyarchaeota archaeon, GCA_003133625.1, 1494
Clone from sediment (11 cm), GU135492, 1364
Clone from peat soil, AB364888, 1423
Clone from acidic fen soil, GU127871, 1424
Clone from boreal peat soil, AB364890, 1425
Clone from acidic fen soil, GU127870, 1423
Clone from acidic fen soil, GU127874, 1424
Clone from rich minerotrophic fen, EU155994, 1466
Clone from wetland soil, KU519585, 1465
Clone from acidic fen soil, GU127873, 1421
Candidatus Bathyarchaeota archaeon, GCA_903884205.1, 1492
Candidatus Bathyarchaeota archaeon, GCA_903901015.1, 1492
Candidatus Bathyarchaeota archaeon, GCA_003151735.1, 1492
Clone from rich minerotrophic fen, EU155990, 1465
Clone from freshwater sediment (groundwater-fed sinkhole), EU910615, 1463
Clone from river sediment (spring pit), JN397860, 1461
Clone from rich minerotrophic fen, EU155991, 1465
PALSA-986 sp003589585, GCF_003589585.1, 1318
Clone from outfall sediment, JF304136, 1463
Clone from Huixian Wetland, MK123366, 1463
Clone from freshwater sediment (microbial mat), AB600452, 1369
Clone from outfall sediment, JF304135, 1464
Clone from estuarine sediment, EU559699, 1466
Clone from rich minerotrophic fen, EU155996, 1463
Clone from municipal wastewater sludge, AF424775, 1469
Clone from river bank, JN397648, 1422
Clone from hot spring, HQ395713, 1423
Clone from freshwater sediment (alkaline crater lake), KT715016, 1349
Clone from landfill leachate, AJ576209, 1425
Clone from Lei-Gong-Huo mud volcano, JQ407234, 1367
Clone from salt marsh sediment, FJ655692, 1424
Clone from Lei-Gong-Huo mud volcano, JX648556, 1370
Clone from estuarine sediment (Kao-Mei wetland), EU420682, 1478
Clone from marine sediment, AB801112, 1425
Clone from marine sediment (subseafloor sediment at the KP-9), JQ989626, 1443
Clone from estuarine sediment, JN605044, 1424
Clone from hot spring sediment, KC682084, 1423
Clone from lake sediment , AJ937877, 1523
Clone from marine sediment, AB801160, 1426
Clone from hot spring sediment, HQ395712, 1424
Clone from hot spring sediment, FJ821636, 1460
Clone from peat soil, AB364906, 1426
Clone from lake sediment, MK076061, 1461
Clone from lake sediment, MK076065, 1461
uncultured archaeon, JQ817906, 1430
uncultured archaeon, JN229609, 1427
uncultured archaeon, AB364908, 1433
uncultured archaeon, AB801162, 1429
uncultured archaeon, JX648590, 1371
uncultured archaeon, KP091069, 1469
uncultured archaeon, AB260058, 1429
uncultured archaeon, KP091048, 1471
uncultured archaeon, AB801155, 1431
uncultured archaeon, KP091051, 1470
uncultured archaeon, KP784731, 1473
Clone from geothermal well, DQ300337, 1343
Clone from hydrothermal seep, KP091066, 1469
Clone from hydrothermal seep, KP090981, 1459
Candidatus Bathyarchaeota archaeon, GCA_011364805.1, 1270
Clone from produced fluid from Niiboli oilfield, HM041916, 1464
Clone from PCR-derived sequence from subsurface geothermal water, AB113634, 1424
Clone from lake, JF262235, 1467
Clone from Obsidian Pool, DQ243757, 1377
Candidatus Bathyarchaeota archaeon, GCA_011373445.1, 1494
Clone from hot spring sediment at temperature of 80 degrees C, EU924227, 1335
Candidatus Bathyarchaeota archaeon, GCA_009889605.1, 1493
Clone from subseafloor sediment at the KP-9, JQ989689, 1442
Clone from subseafloor sediment at the Tainan Ridge, JN123702, 1421
Clone from subseafloor sediment at Porcupine, JN229432, 1460
Clone from marine sediment, HQ700670, 1468
Clone from Amsterdam Mud Volcano sapropel sediment, East Mediterranean Sea, FJ649522, 1463
Clone from subseafloor sediment at the Tainan Ridge, JN123708, 1410
Clone from marine sediment, HQ700680, 1461
Clone from South China Sea marine sediment, HQ214612, 1460
Clone from marine sediment, AB801147, 1425
Clone from clay from marine sediments 31 meters below sea floor depth collected from drilling site U, JREQ01002636, 1466
Clone from subseafloor sediment at the 96 Ridge, JQ989568, 1425
Clone from marine sediment, KX581159, 1496
Candidatus Bathyarchaeota archaeon, GCA_011053435.1, 1494
Clone from saturated zone of the Hanford Site 300 Area subsurface, HM187485, 1337
Clone from peat soil, AB364895, 1428
Clone from peat soil, AB364893, 1425
Clone from Huixian Wetland, MK123376, 1460
Clone from freshwater iron-rich microbial mat, AB722184, 1359
Clone from Huixian Wetland, MK123373, 1460
Clone from peat soil, AB364904, 1423
Clone from saturated zone of the Hanford Site 300 Area subsurface, HM187510, 1358
Clone from peat soil, AB364903, 1426
Clone from peat soil, AB364900, 1425
Clone from hot spring sediment, KC682074, 1423
Clone from lake sediment, AM076830, 1438
TB1b (17)
Bathycorpusculum acetaminoxidans (3)
Bathycorpusculum hydrogenotrophicum (4)
Bathycorpusculum termitum (4)
TB2 (13)
Cubitermitinae_cluster (8)
TB1a (27)
Bathycorpusculum_fermentans (3)
Cluster_M (8)
Bathycorpusculum (69)
Bathycorpusculaceae (119)
freshwater sediment (7)
salt marsh sediment (5)
anaerobic digestor (2)
anaerobic digestor (6)
anaerobic digestor (2)
Sediment_anaerobic_digester (22)
Peat_soil_freshwater_sediment (9)
Peat_soil_freshwater_sediment (4)
Wetland soil and freshwater sediment (5)
Freshwater_sediment (5)
Marine_sediment (3)
Marine_estuarine_sediment (11)
Hot_spring_sediment (2)
Peat soil and freshwater sediment (3)
Subgroup_13 (11)
g__DSZD01 (2)
g__DRVV01 (5)
Subgroup_1 (12)
Subgroup_5b (8)
Subgroup_5c (2)
0.10

Figure S8

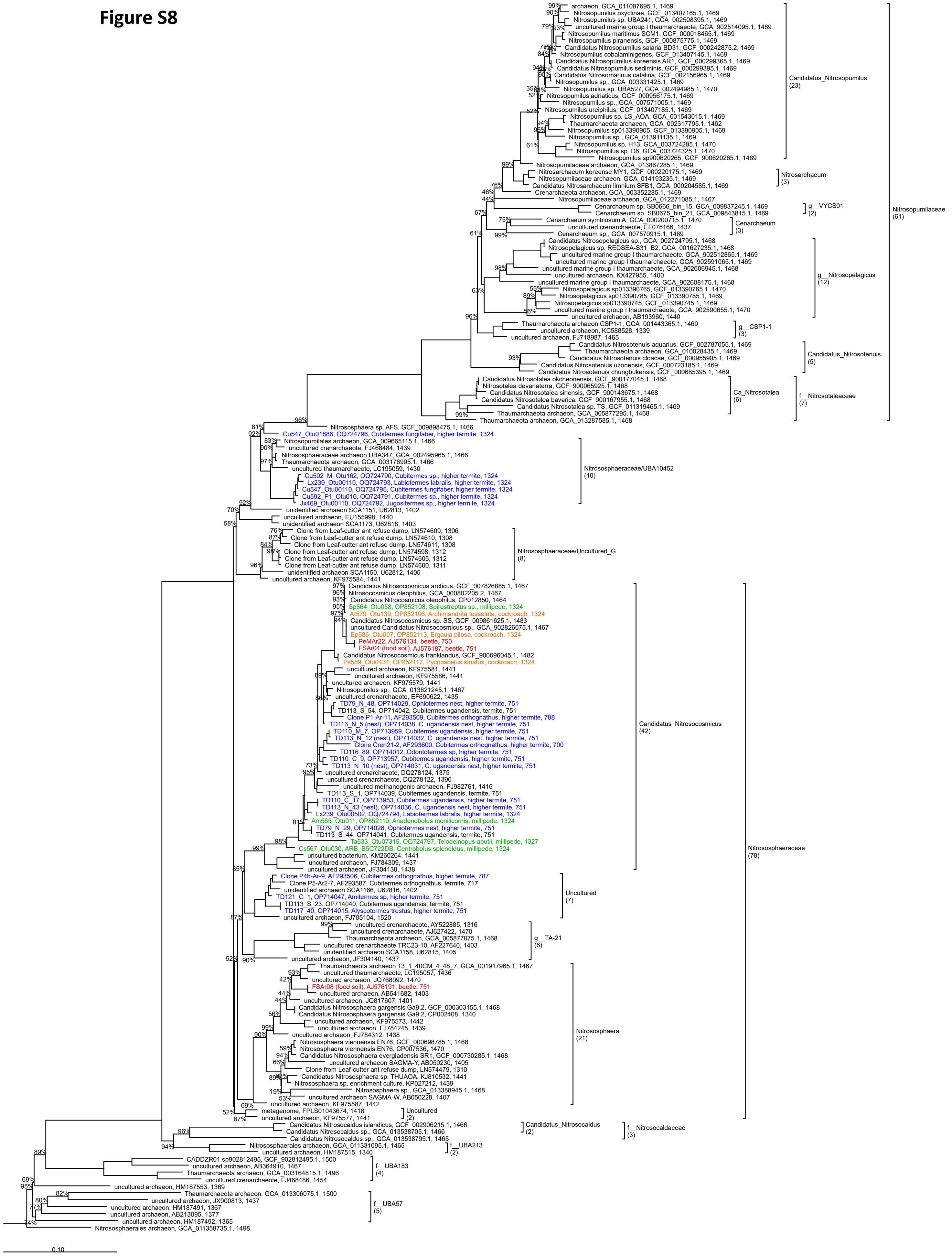

archaeon, GCA_011087695.1, 1469
Nitrosopumilus oxyclinae, GCF_013407165.1, 1469
Nitrosopumilus sp. UBA241, GCA_002508395.1, 1469
uncultured marine group I thaumarchaeote, GCA_902514095.1, 1469
Nitrosopumilus maritimus SCM1, GCF_000018465.1, 1469
Nitrosopumilus piranensis, GCF_000875775.1, 1469
Candidatus Nitrosopumilus salaria BD31, GCF_000242875.2, 1469
Nitrosopumilus cobalaminigenes, GCF_013407145.1, 1469
Candidatus Nitrosopumilus koreensis AR1, GCF_000299365.1, 1469
Candidatus Nitrosopumilus sediminis, GCF_000299395.1, 1469
Candidatus Nitrosomarinus catalina, GCF_002156965.1, 1469
Nitrosopumilus sp., GCA_003331425.1, 1469
Nitrosopumilus sp. UBA527, GCA_002494985.1, 1470
Nitrosopumilus adriaticus, GCF_000956175.1, 1469
Nitrosopumilus sp., GCA_007571005.1, 1469
Nitrosopumilus ureiphilus, GCF_013407185.1, 1469
Nitrosopumilus sp. LS_AOA, GCA_001543015.1, 1469
Thaumarchaeota archaeon, GCA_002317795.1, 1462
Nitrosopumilus sp013390905, GCF_013390905.1, 1469
Nitrosopumilus sp., GCA_013911135.1, 1469
Nitrosopumilus sp. H13, GCA_003724285.1, 1470
Nitrosopumilus sp. D6, GCA_003724325.1, 1470
Nitrosopumilus sp900620265, GCF_900620265.1, 1469
Candidatus_Nitrosopumilus (23)
Nitrosopumilaceae archaeon, GCA_013867285.1, 1469
Nitrosarchaeum koreense MY1, GCF_000220175.1, 1469
Nitrosopumilaceae archaeon, GCA_014193235.1, 1469
Candidatus Nitrosarchaeum limnium SFB1, GCA_000204585.1, 1469
Nitrosarchaeum (3)
Crenarchaeota archaeon, GCA_003352285.1, 1469
Nitrosopumilaceae archaeon, GCA_012271085.1, 1467
Cenarchaeum sp. SB0666_bin_15, GCA_009837245.1, 1469
Cenarchaeum sp. SB0675_bin_21, GCA_009843815.1, 1469
g__VYCS01 (2)
Cenarchaeum symbiosum A, GCA_000200715.1, 1470
uncultured crenarchaeote, EF076166, 1437
Cenarchaeum sp., GCA_007570915.1, 1469
Cenarchaeum (3)
Candidatus Nitrosopelagicus sp., GCA_002724795.1, 1468
Nitrosopelagicus sp. REDSEA-S31_B2, GCA_001627235.1, 1468
uncultured marine group I thaumarchaeote, GCA_902512865.1, 1469
uncultured marine group I thaumarchaeote, GCA_902591065.1, 1469
uncultured marine group I thaumarchaeote, GCA_902606945.1, 1468
uncultured archaeon, KX427955, 1400
uncultured marine group I thaumarchaeote, GCA_902608175.1, 1468
Nitrosopelagicus sp013390765, GCF_013390765.1, 1470
Nitrosopelagicus sp013390785, GCF_013390785.1, 1469
Nitrosopelagicus sp013390745, GCF_013390745.1, 1469
uncultured marine group I thaumarchaeote, GCA_902590655.1, 1470
uncultured archaeon, AB193960, 1440
g__Nitrosopelagicus (12)
Thaumarchaeota archaeon CSP1-1, GCA_001443365.1, 1469
uncultured archaeon, KC588528, 1339
uncultured archaeon, FJ718987, 1465
g__CSP1-1 (3)
Candidatus Nitrosotenuis aquarius, GCF_002787055.1, 1469
Thaumarchaeota archaeon, GCA_010028435.1, 1469
Candidatus Nitrosotenuis cloacae, GCF_000955905.1, 1469
Candidatus Nitrosotenuis uzonensis, GCF_000723185.1, 1469
Candidatus Nitrosotenuis chungbukensis, GCF_000685395.1, 1469
Candidatus_Nitrosotenuis (5)
Candidatus Nitrosotalea okcheonensis, GCF_900177045.1, 1468
Nitrosotalea devanaterra, GCF_900065925.1, 1468
Candidatus Nitrosotalea sinensis, GCF_900143675.1, 1468
Candidatus Nitrosotalea bavarica, GCF_900167955.1, 1468
Candidatus Nitrosotalea sp. TS, GCF_011319465.1, 1469
Thaumarchaeota archaeon, GCA_005877295.1, 1468
Thaumarchaeota archaeon, GCA_013287585.1, 1468
Ca_Nitrosotalea (6)
f__Nitrosotaleaceae (7)
Nitrosopumilaceae (61)
Nitrososphaera sp. AFS, GCF_009898475.1, 1466
Cu547_Otu01886, OQ724796, Cubitermes fungifaber, higher termite, 1324
Nitrosopumilales archaeon, GCA_009665115.1, 1466
uncultured crenarchaeote, FJ468484, 1439
Nitrososphaeraceae archaeon UBA347, GCA_002495965.1, 1466
Thaumarchaeota archaeon, GCA_003176995.1, 1466
uncultured thaumarchaeote, LC195059, 1430
Cu592_M_Otu162, OQ724790, Cubitermes sp., higher termite, 1324
Lx239_Otu00110, OQ724793, Labiotermes labralis, higher termite, 1324
Cu547_Otu00110, OQ724795, Cubitermes fungifaber, higher termite, 1324
Cu592_P1_Otu016, OQ724791, Cubitermes sp., higher termite, 1324
Jx469_Otu00110, OQ724792, Jugositermes sp., higher termite, 1324
Nitrososphaeraceae/UBA10452 (10)
unidentified archaeon SCA1151, U62813, 1402
uncultured archaeon, EU155998, 1440
unidentified archaeon SCA1173, U62818, 1403
Clone from Leaf-cutter ant refuse dump, LN574609, 1306
Clone from Leaf-cutter ant refuse dump, LN574610, 1308
Clone from Leaf-cutter ant refuse dump, LN574611, 1308
Clone from Leaf-cutter ant refuse dump, LN574598, 1312
Clone from Leaf-cutter ant refuse dump, LN574605, 1312
Clone from Leaf-cutter ant refuse dump, LN574600, 1311
unidentified archaeon SCA1150, U62812, 1405
uncultured archaeon, KF975584, 1441
Nitrososphaeraceae/Uncultured_G (8)
Candidatus Nitrosocosmicus arcticus, GCF_007826885.1, 1467
Nitrosocosmicus oleophilus, GCA_000802205.2, 1467
Candidatus Nitrocosmicus oleophilus, CP012850, 1464
Sp564_Otu058, OP852108, Spirostreptus sp., millipede, 1324
At579_Otu139, OP852106, Archimandrita tesselata, cockroach, 1324
Candidatus Nitrosocosmicus sp. SS, GCF_009861625.1, 1483
uncultured Candidatus Nitrosocosmicus sp., GCA_902826075.1, 1467
Ep586_Otu007, OP852113, Ergaula pilosa, cockroach, 1324
PeMAr22, AJ576134, beetle, 750
FSAr04 (food soil), AJ576187, beetle, 751
Candidatus Nitrosocosmicus franklandus, GCF_900696045.1, 1482
Ps589_Otu0431, OP852117, Pycnoscelus striatus, cockroach, 1324
uncultured archaeon, KF975581, 1441
uncultured archaeon, KF975586, 1441
uncultured archaeon, KF975579, 1441
Nitrosopumilus sp., GCA_013821245.1, 1467
uncultured crenarchaeote, EF690622, 1435
TD79_N_48, OP714029, Ophiotermes nest, higher termite, 751
TD113_S_54, OP714042, Cubitermes ugandensis, termite, 751
Clone P1-Ar-11, AF293509, Cubitermes orthognathus, higher termite, 788
TD113_N_5 (nest), OP714038, C. ugandensis nest, higher termite, 751
TD110_M_7, OP713959, Cubitermes ugandensis, higher termite, 751
TD113_N_12 (nest), OP714032, C. ugandensis nest, higher termite, 751
Clone Cren21-2, AF293600, Cubitermes orthognathus, higher termite, 700
TD116_89, OP714012, Odontotermes sp, higher termite, 751
TD110_C_9, OP713957, Cubitermes ugandensis, higher termite, 751
TD113_N_10 (nest), OP714031, C. ugandensis nest, higher termite, 751
uncultured crenarchaeote, DQ278124, 1375
uncultured crenarchaeote, DQ278122, 1390
uncultured methanogenic archaeon, FJ982761, 1416
TD113_S_1, OP714039, Cubitermes ugandensis, termite, 751
TD110_C_17, OP713953, Cubitermes ugandensis, higher termite, 751
TD113_N_43 (nest), OP714036, C. ugandensis nest, higher termite, 751
Lx239_Otu00502, OQ724794, Labiotermes labralis, higher termite, 1324
Am565_Otu011, OP852110, Anadenobolus monilicornis, millipede, 1324
TD79_N_29, OP714028, Ophiotermes nest, higher termite, 751
TD113_S_44, OP714041, Cubitermes ugandensis, termite, 751
Ta633_Otu07315, OQ724797, Telodeinopus aoutii, millipede, 1327
Cs567_Otu030, ARB_B5C722DB, Centrobolus splendidus, millipede, 1324
uncultured bacterium, KM260264, 1441
uncultured archaeon, FJ784309, 1437
uncultured archaeon, JF304138, 1438
Candidatus_Nitrosocosmicus (42)
Clone P4b-Ar-9, AF293506, Cubitermes orthognathus, higher termite, 787
Clone P5-Ar2-7, AF293587, Cubitermes orthognathus, termite, 717
unidentified archaeon SCA1166, U62816, 1402
TD121_C_1, OP714047, Amitermes sp, higher termite, 751
TD113_S_23, OP714040, Cubitermes ugandensis, termite, 751
TD117_40, OP714015, Alyscotermes trestus, higher termite, 751
uncultured archaeon, FJ705104, 1520
Uncultured (7)
uncultured crenarchaeote, AY522885, 1316
uncultured crenarchaeote, AJ627422, 1470
Thaumarchaeota archaeon, GCA_005877075.1, 1468
uncultured crenarchaeote TRC23-10, AF227640, 1403
unidentified archaeon SCA1158, U62815, 1405
uncultured archaeon, JF304140, 1437
g__TA-21 (6)
Thaumarchaeota archaeon 13_1_40CM_4_48_7, GCA_001917965.1, 1467
uncultured thaumarchaeote, LC195057, 1436
uncultured archaeon, JQ768092, 1470
FSAr08 (food soil), AJ576191, beetle, 751
uncultured archaeon, AB541682, 1403
uncultured archaeon, JQ817607, 1401
Candidatus Nitrososphaera gargensis Ga9.2, GCF_000303155.1, 1468
Candidatus Nitrososphaera gargensis Ga9.2, CP002408, 1340
uncultured archaeon, KF975573, 1442
uncultured archaeon, FJ784245, 1439
uncultured archaeon, FJ784312, 1438
Nitrososphaera viennensis EN76, GCF_000698785.1, 1468
Nitrososphaera viennensis EN76, CP007536, 1470
Candidatus Nitrososphaera evergladensis SR1, GCF_000730285.1, 1468
uncultured archaeon SAGMA-Y, AB050230, 1405
Clone from Leaf-cutter ant refuse dump, LN574479, 1310
Candidatus Nitrososphaera sp. THUAOA, KJ810532, 1441
Nitrososphaera sp. enrichment culture, KP027212, 1439
Nitrososphaera sp., GCA_013388945.1, 1468
uncultured archaeon SAGMA-W, AB050228, 1407
uncultured archaeon, KF975587, 1442
Nitrososphaera (21)
metagenome, FPLS01043674, 1418
uncultured archaeon, KF975577, 1441
Uncultured (2)
Nitrososphaeraceae (78)
Candidatus Nitrosocaldus islandicus, GCF_002906215.1, 1466
Candidatus Nitrosocaldus sp., GCA_013538705.1, 1466
Candidatus Nitrosocaldus sp., GCA_013538795.1, 1465
Candidatus_Nitrosocaldus (2)
f__Nitrosocaldaceae (3)
Nitrososphaerales archaeon, GCA_011331095.1, 1465
uncultured archaeon, HM187515, 1340
f__UBA213 (2)
CADDZR01 sp902812495, GCF_902812495.1, 1500
uncultured archaeon, AB364910, 1467
Thaumarchaeota archaeon, GCA_003164815.1, 1496
uncultured crenarchaeote, FJ468486, 1454
f__UBA183 (4)
uncultured archaeon, HM187553, 1369
Thaumarchaeota archaeon, GCA_013306075.1, 1500
uncultured archaeon, JX000813, 1437
uncultured archaeon, HM187491, 1367
uncultured archaeon, AB213095, 1377
uncultured archaeon, HM187492, 1365
Nitrososphaerales archaeon, GCA_011358735.1, 1498
f__UBA57 (5)
0.10
